# Supplementary material for: Acquisition of chromosome instability is a mechanism to evade oncogene addiction
Source: EMBO Mol Med. 2020 Feb 6;12(3):e10941. doi: 10.15252/emmm.201910941 (PMC7059010; doi:10.15252/emmm.201910941)
Supplement: Supplementary file 1 — Appendix [file EMMM-12-e10941-s001.pdf]

Acquisition of chromosome instability is a mechanism to evade oncogene addiction

Lorena Salgueiro, Christopher Buccitelli, Konstantina Rowald, Kalman Somogyi, Sridhar Kandala, Jan O. Korbel and Rocio Sotillo

Appendix

Appendix Table S1
Appendix Table S2
Appendix Table S3
Appendix Table S4

Appendix Table S1

**Figure 1B**	P value	
K primaryT vs. K non-Regr	****	0.0001
K primaryT vs. KM primaryT	*	0.0290
K non-Regr vs. KM non-Regr	n.s	0.5209

Appendix Table S2

**Figure 4B**	Significant?	P value
Day 1	No	0.846833
Day 2	No	0.365634
Day 3	No	0.844099
Day 4	No	0.520593
Day 5	No	0.053524
Day 6	No	0.20104
Day 7	No	0.45213

Appendix Table S3

**Figure 5B**	p value	mean difference	p value
no MET vehicle vs. cMET ampl vehicle	ns	0.9519	
no MET vehicle vs. no MET tepotinib	ns	0.2133	
cMET ampl vehicle vs. cMET ampl tepotinib	**	-1.764	0.024

Appendix Table S4

**Figure 5D**	p value	mean difference
no cMet Ctl vs. no cMet Tepotinib	ns	0,2698
cMet Ampl.Ctl vs. no cMet Ctl	****	17,38
cMet Ampl.Ctl vs. cMet Ampl.Tepotinib	****	16,59

**Figure 5E**		
no cMet Ctl vs. no cMet Tepotinib	ns	-0,8844
cMet Ampl.Ctl vs. no cMet Ctl	ns	3,547
cMet Ampl.Ctl vs. cMet Ampl.Tepotinib	*	-7,03
