# Supplementary material for: Acquisition of chromosome instability is a mechanism to evade oncogene addiction
Source: EMBO Mol Med. 2020 Feb 6;12(3):e10941. doi: 10.15252/emmm.201910941 (PMC7059010; doi:10.15252/emmm.201910941)
Supplement: Supplementary file 2 — Expanded View Figures PDF [file EMMM-12-e10941-s002.pdf]

Expanded View Figures

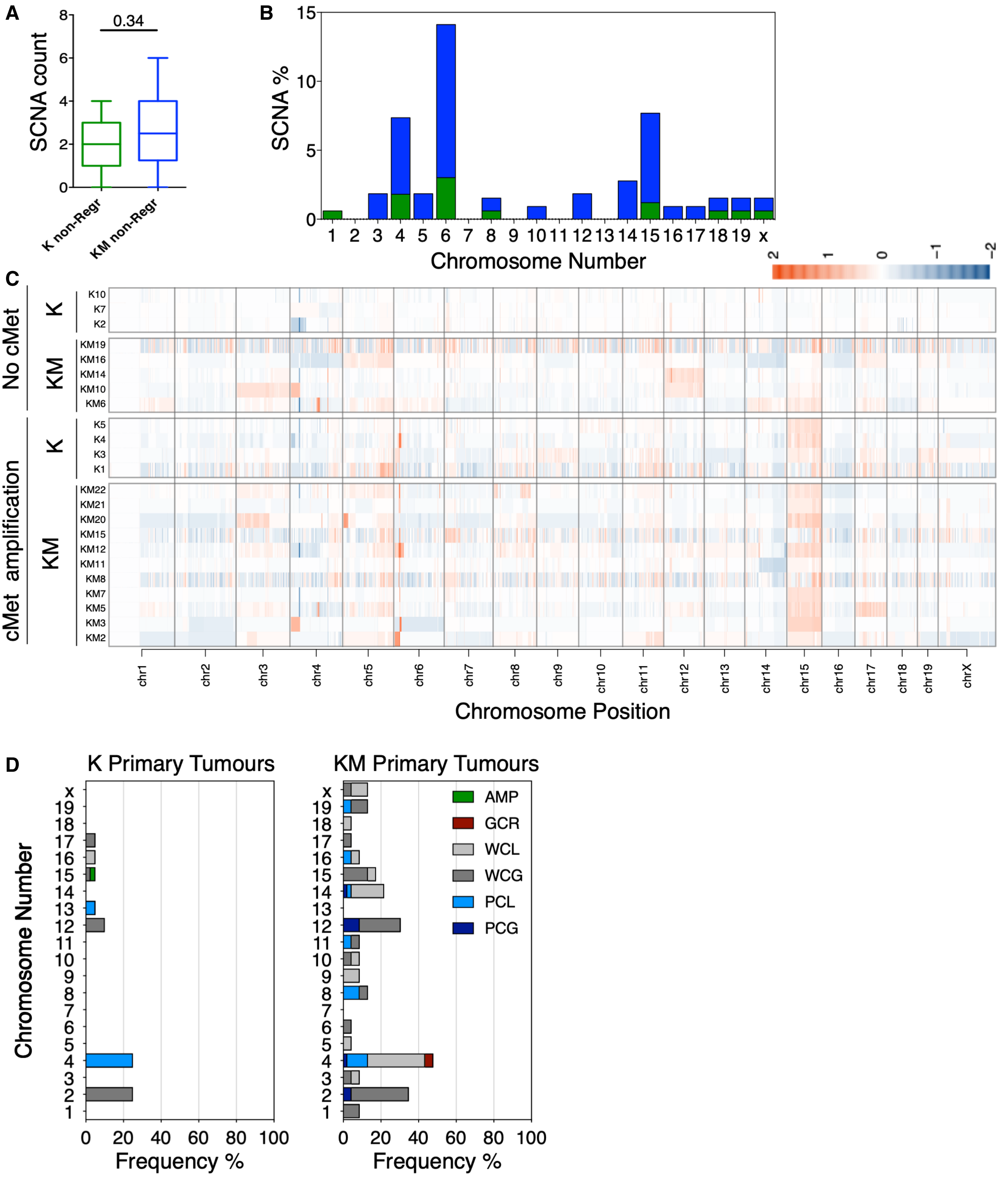

Figure EV1.

 © 2020 The Authors

Figure EV1. Related to Fig 2. Somatic copy number alterations in K and KM primary and non-regressed tumours.

A Average of somatic copy number alteration (SCNAs) in K and KM non-regressed tumours. Unpaired *t*-test, $P = 0.34$. Boxes and whiskers represent min to max values. The central line represents the median.
B Percentage of SCNAs per chromosome in K (green) and KM (blue) non-regressed tumours.
C Heatmap of all chromosomes in the non-regressed tumours.
D Frequency of somatic copy number alterations per chromosome in Kras and Kras/Mad2 primary tumours. Focal amplification (AMP, dark green), whole chromosome gain (WCG, dark grey) and loss (WCL, light grey), partial chromosome gain (PCG, dark blue) and loss (PCL, light blue) and gross chromosomal rearrangement (GCR, red).

Source data are available online for this figure.

Figure EV2. Related to Fig 2. cMet analysis in non-regressed tumours.

A Genome-wide $\log_2$ of chromosome 6 of control mammary gland and $\log_2$-ratio plots of K and KM non-regressed tumour biopsies showing no amplification in the control (upper panels) and a small amplification in the non-regressed tumours (middle and bottom panels).
B Quantitative RT–PCR analysis of cMet in 10 K and 22 KM non-regressed tumours.
C Correlation analysis of cMet mRNA and gene amplification in K (green) and KM (blue) non-regressed breast tumours.

Source data are available online for this figure.

© 2020 The Authors

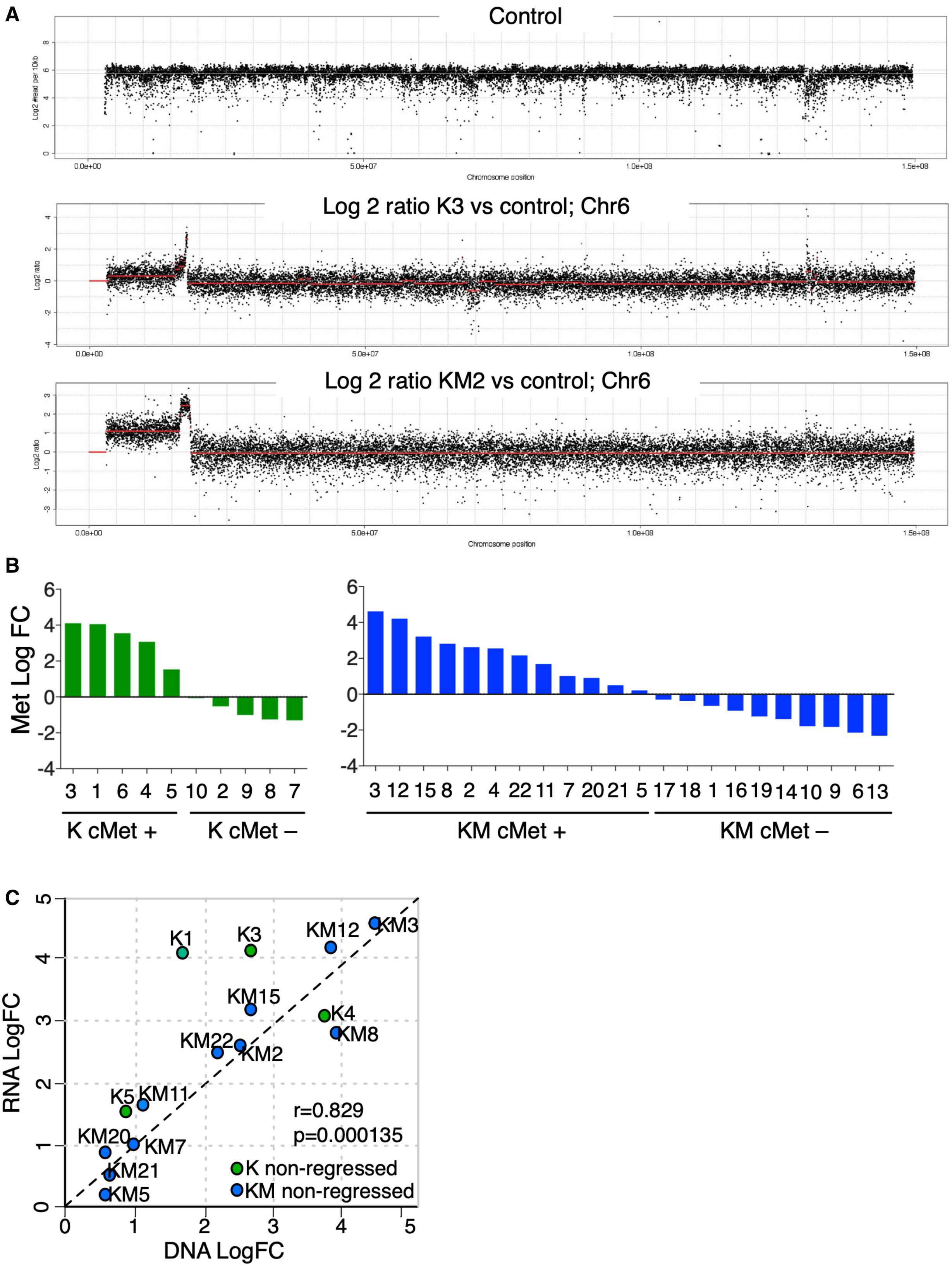

Figure EV2.

 © 2020 The Authors

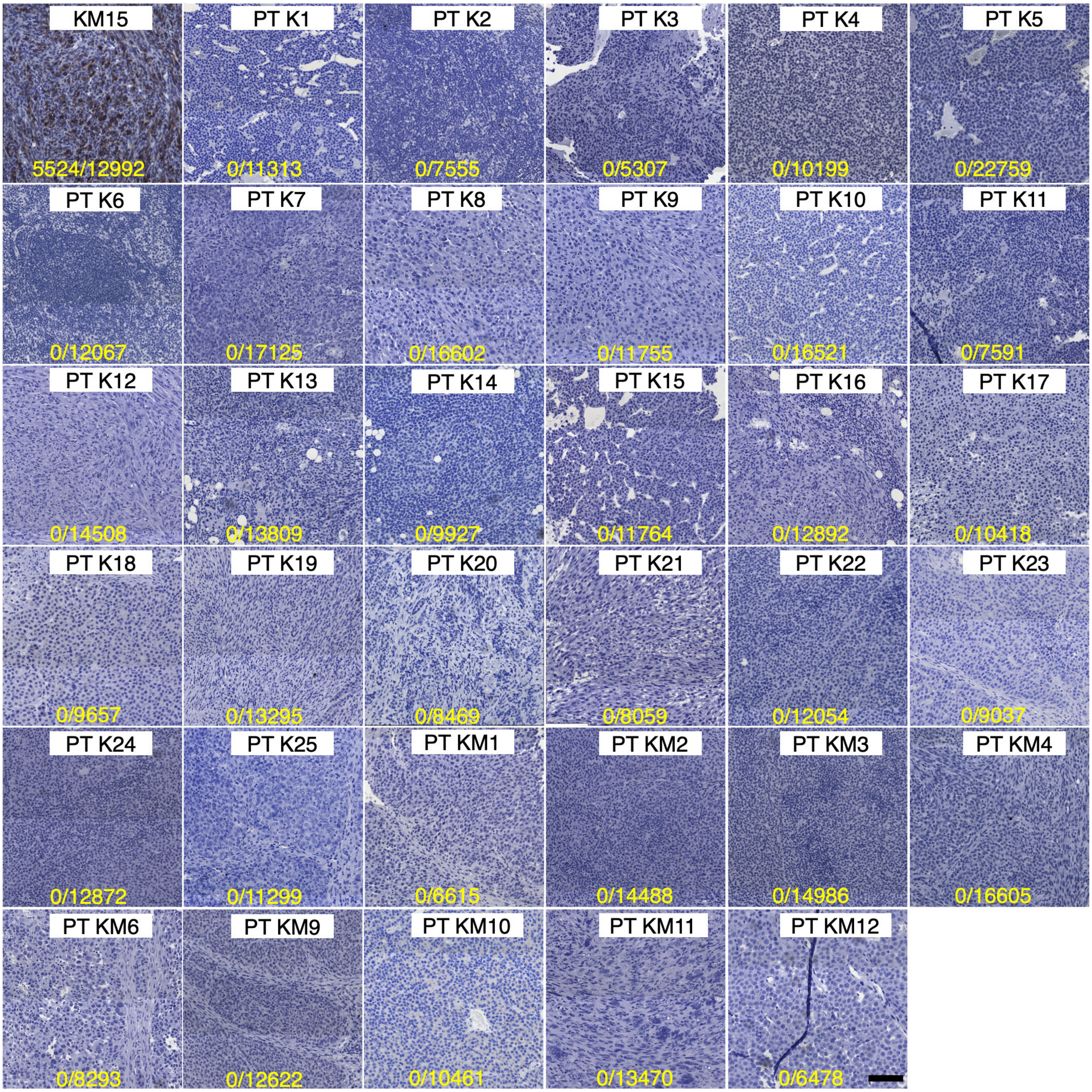

Figure EV3. Related to Fig 3. cMet staining in primary tumours.

Immunostaining of phospho-cMet in one non-regressed tumour (KM15) as a positive control (as shown in Fig 2C) and 34 primary tumours (PT). Yellow numbers indicate the total number of cMet-positive cells/total number of cells counted. Scale bar 100 μm.

Source data are available online for this figure.

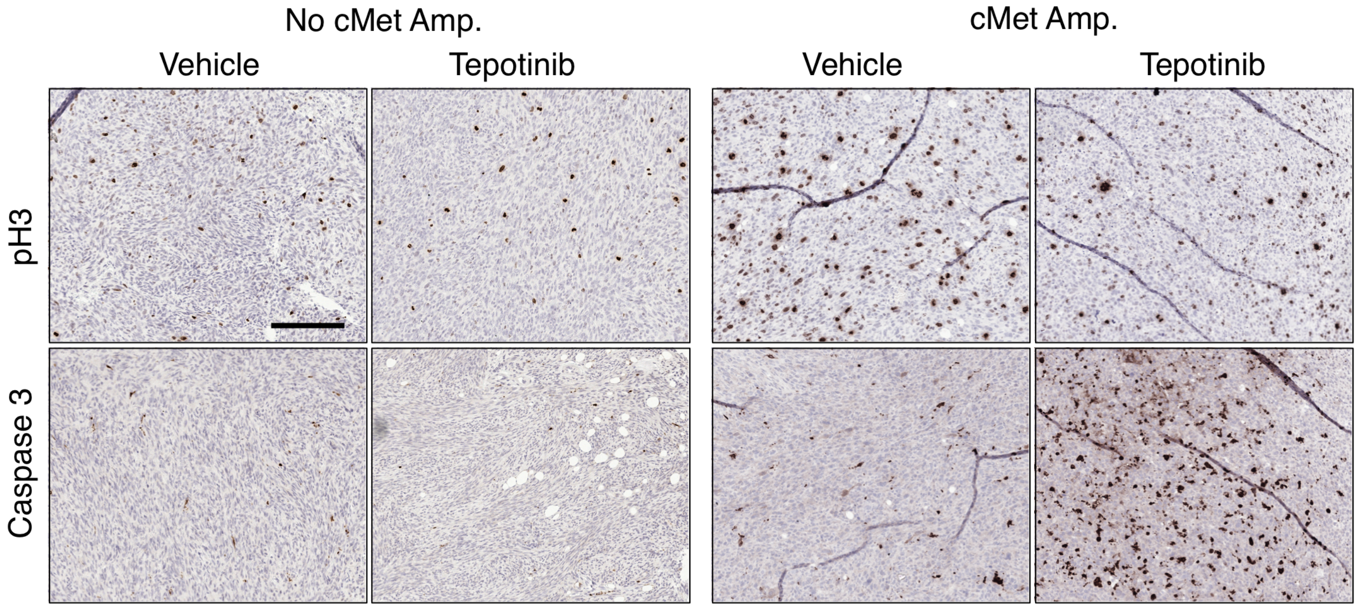

Figure EV4. Related to Fig 5. Proliferation and apoptosis in non-regressed tumours.

Immunostaining of pH3 and caspase 3 in tumours with and without cMet amplification after treatment with tepotinib or vehicle control. Scale bar 200 µm.
Source data are available online for this figure.

 © 2020 The Authors
