# Supplementary material for: Acquisition of chromosome instability is a mechanism to evade oncogene addiction
Source: EMBO Mol Med. 2020 Feb 6;12(3):e10941. doi: 10.15252/emmm.201910941 (PMC7059010; doi:10.15252/emmm.201910941)
Supplement: Supplementary file 3 — Source Data for Expanded View and Appendix [file EMMM-12-e10941-s010.zip › Appendix.docx]

Acquisition of chromosome instability is a mechanism to evade oncogene addiction

Lorena Salgueiro^1^, Christopher Buccitelli^2#^, Konstantina Rowald^1^, Kalman Somogyi^1^, Sridhar Kandala^1^, Jan O. Korbel^2^ and Rocio Sotillo^1,3*^

Key words: chromosome instability, resistance, breast cancer, mouse models, cMet

Table of content

**Supplemental Tables**

**Table S1:** Statistical analysis of the percentage of mitotic errors in Fig 1B.

**Table S2**: Statistical analysis of relative volume in Fig 4B.

**Table S3:** Statistical analysis of relative volume in Fig 5B.

**Table S4:** Statistical analysis of the percentage of pH3 positive cells in Fig 5D.

**Table S5**: Statistical analysis of the percentage of caspase 3 positive cells in Fig 5E.

**Table S6**: Statistical analysis of the number of SCNA in Fig EV1 A.

**Supplemental Tables**

**Table S1:** Statistical analysis of the percentage of mitotic errors in Fig 1B.

**Table S2**: Statistical analysis of relative volume in Fig 4B.

**Table S3:** Statistical analysis of relative volume in Fig 5B.

**Table S4:** Statistical analysis of the percentage of pH3 positive cells in Fig 5D.

**Table S5**: Statistical analysis of the percentage of caspase 3 positive cells in Fig 5E.

**Table S6**: Statistical analysis of the number of SCNA in Fig EV1 A.
