# Supplementary figures and images for: Acquisition of chromosome instability is a mechanism to evade oncogene addiction (part 1 of 2)
Source: EMBO Mol Med. 2020 Feb 6;12(3):e10941. doi: 10.15252/emmm.201910941 (PMC7059010; doi:10.15252/emmm.201910941)

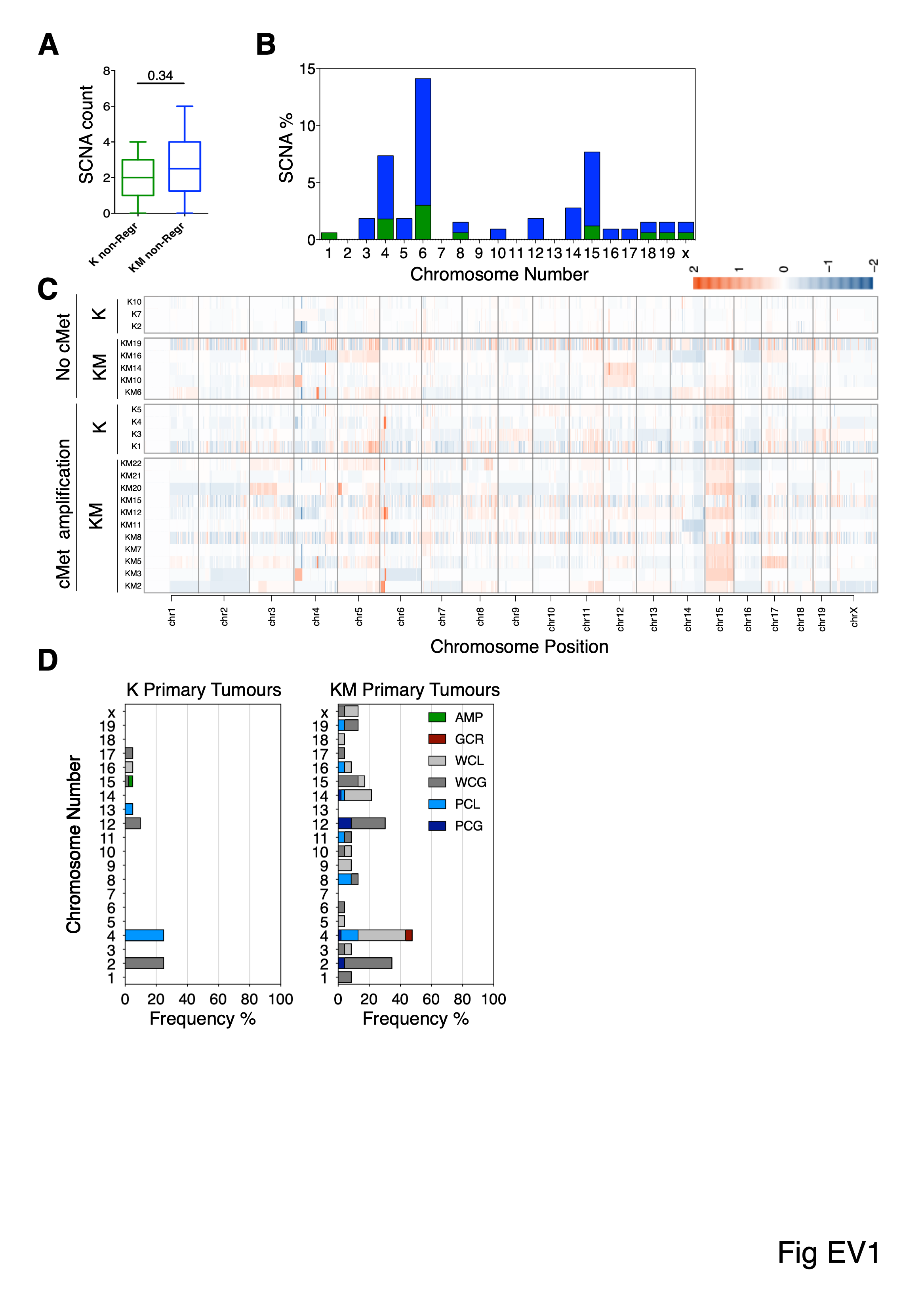

Supplement: Supplementary file 3 — Source Data for Expanded View and Appendix [file EMMM-12-e10941-s010.zip › Fig EV1/Fig EV1.tiff]

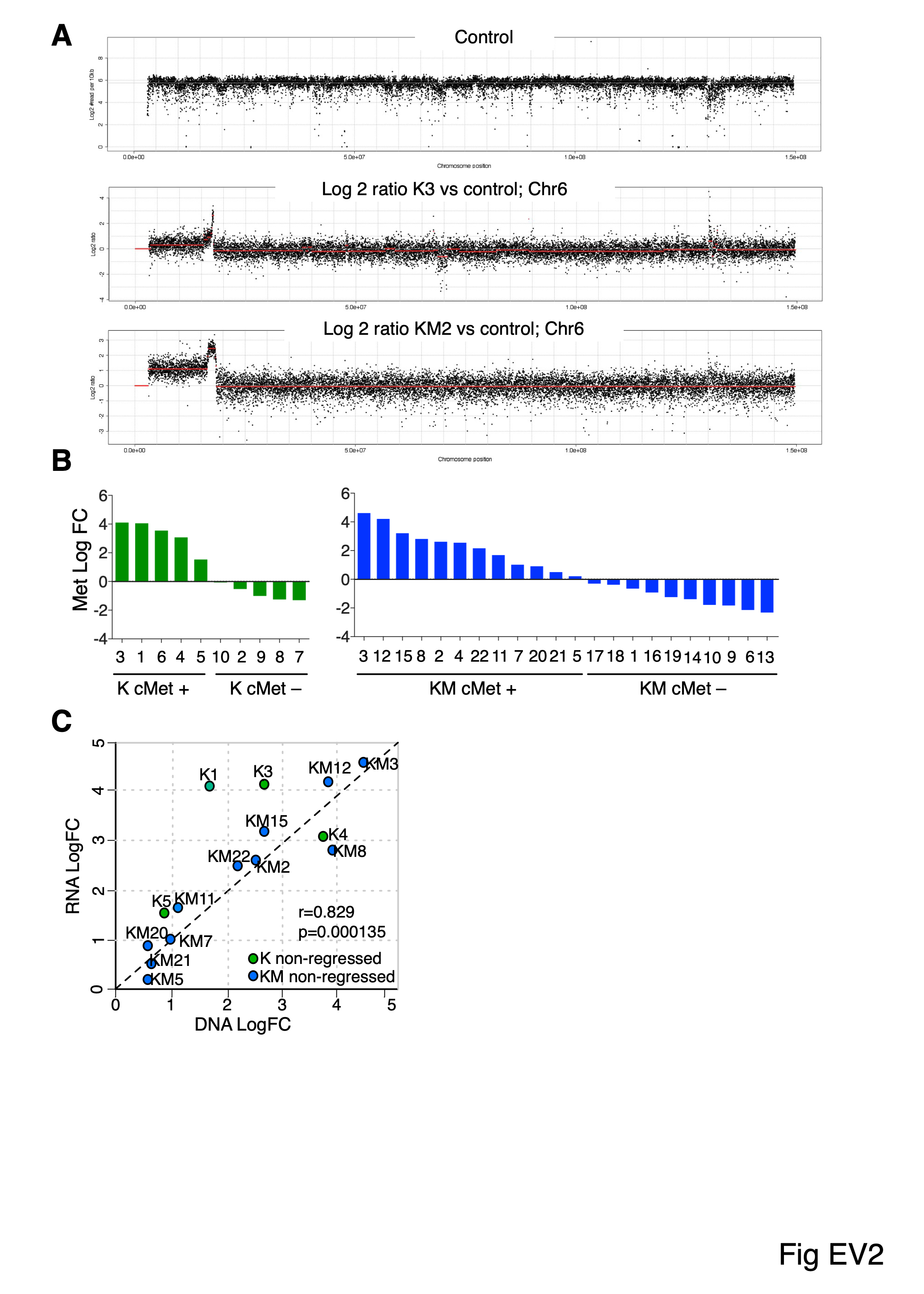

Supplement: Supplementary file 3 — Source Data for Expanded View and Appendix [file EMMM-12-e10941-s010.zip › Fig EV2/Fig EV2.tiff]

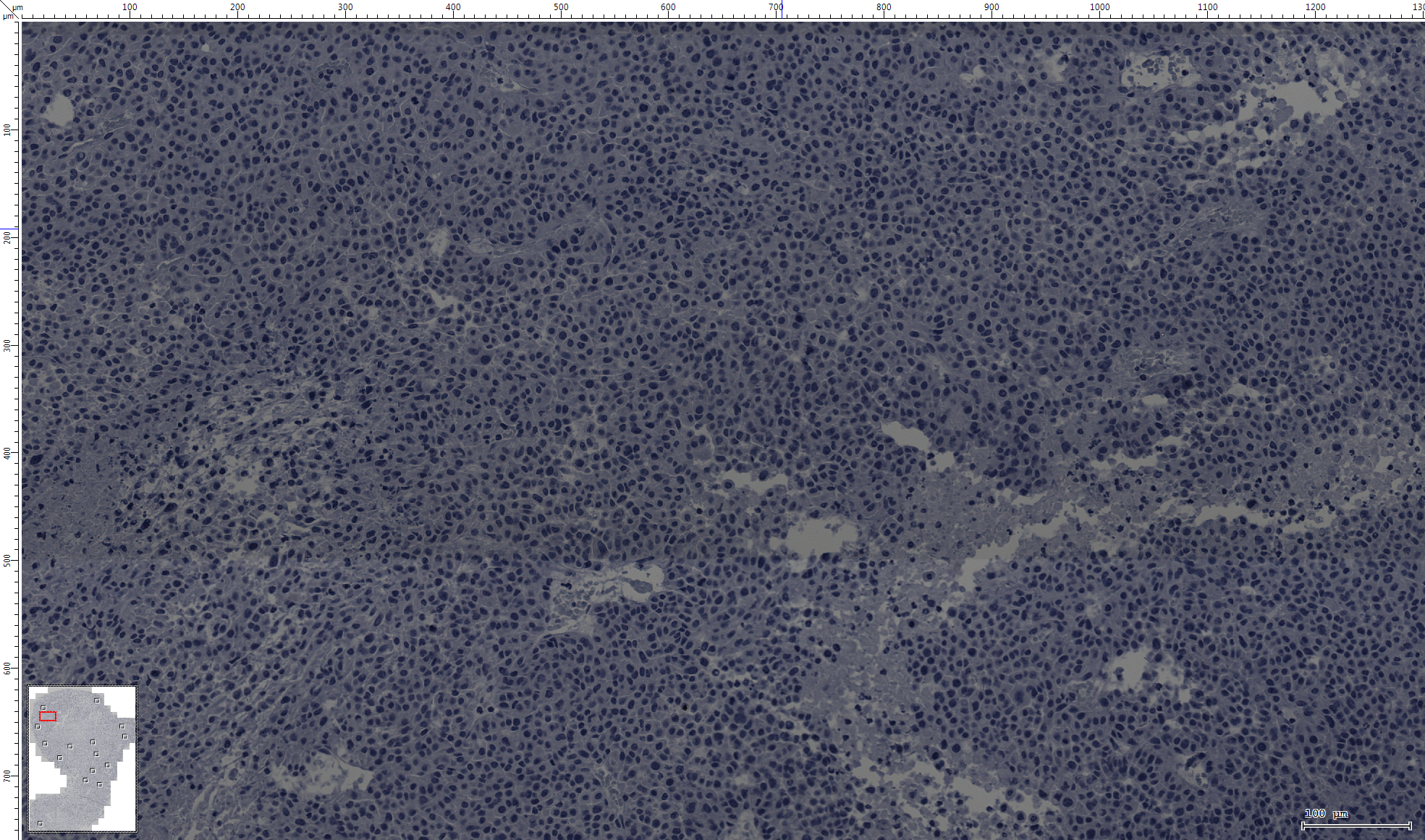

Supplement: Supplementary file 3 — Source Data for Expanded View and Appendix [file EMMM-12-e10941-s010.zip › Fig EV3/PT K17.TIFF]

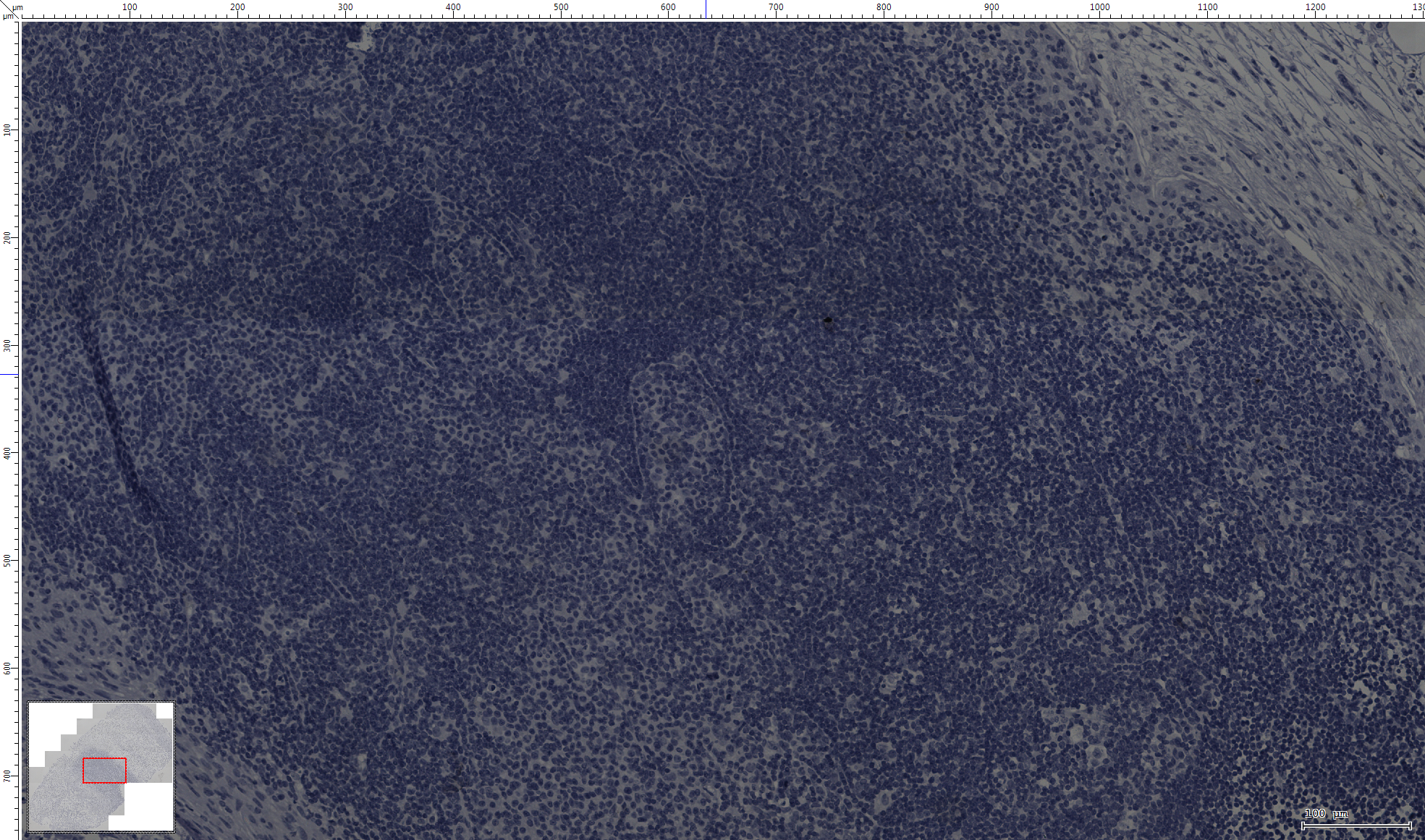

Supplement: Supplementary file 3 — Source Data for Expanded View and Appendix [file EMMM-12-e10941-s010.zip › Fig EV3/PT K2.TIFF]

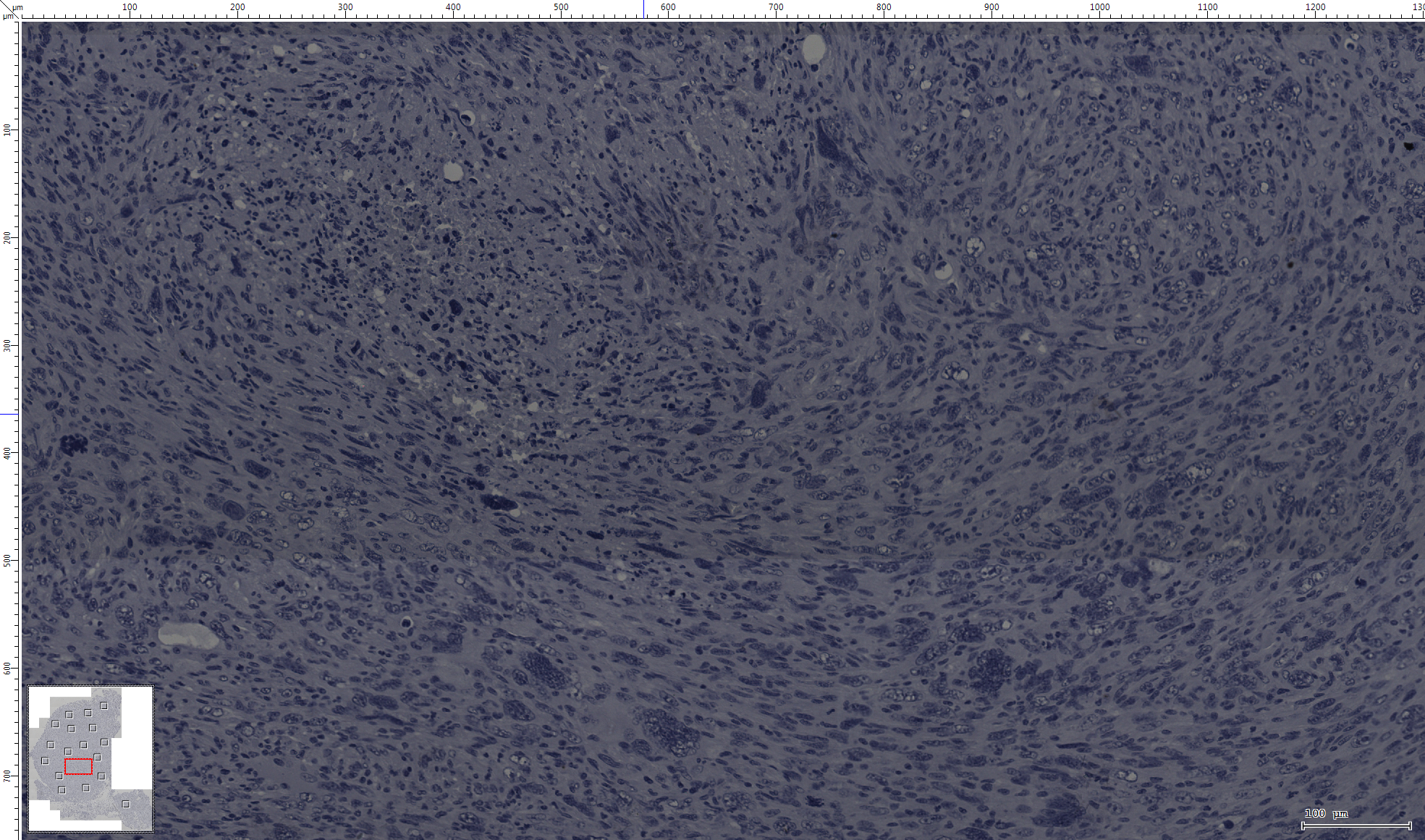

Supplement: Supplementary file 3 — Source Data for Expanded View and Appendix [file EMMM-12-e10941-s010.zip › Fig EV3/PT KM11.TIFF]

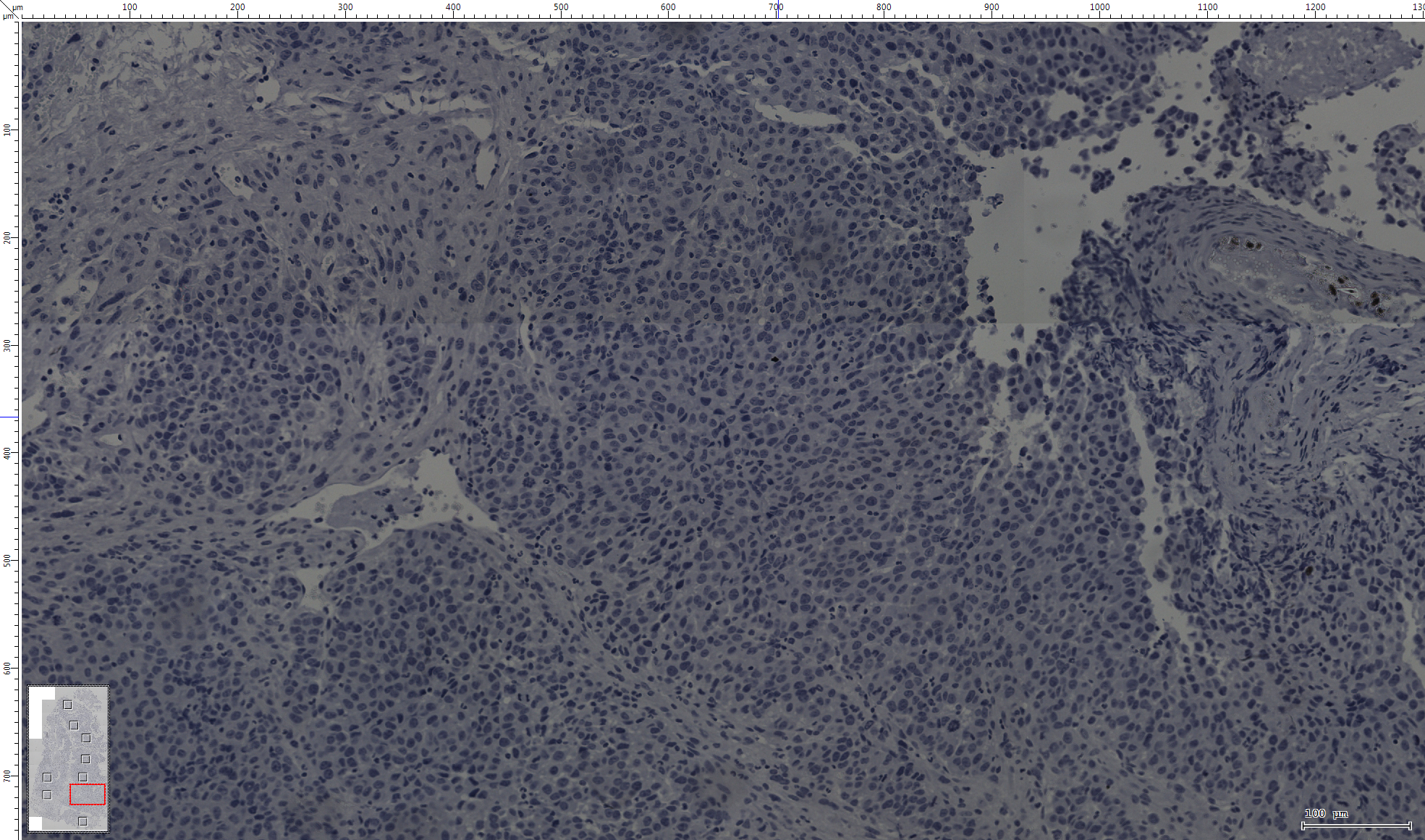

Supplement: Supplementary file 3 — Source Data for Expanded View and Appendix [file EMMM-12-e10941-s010.zip › Fig EV3/PT KM1.TIFF]

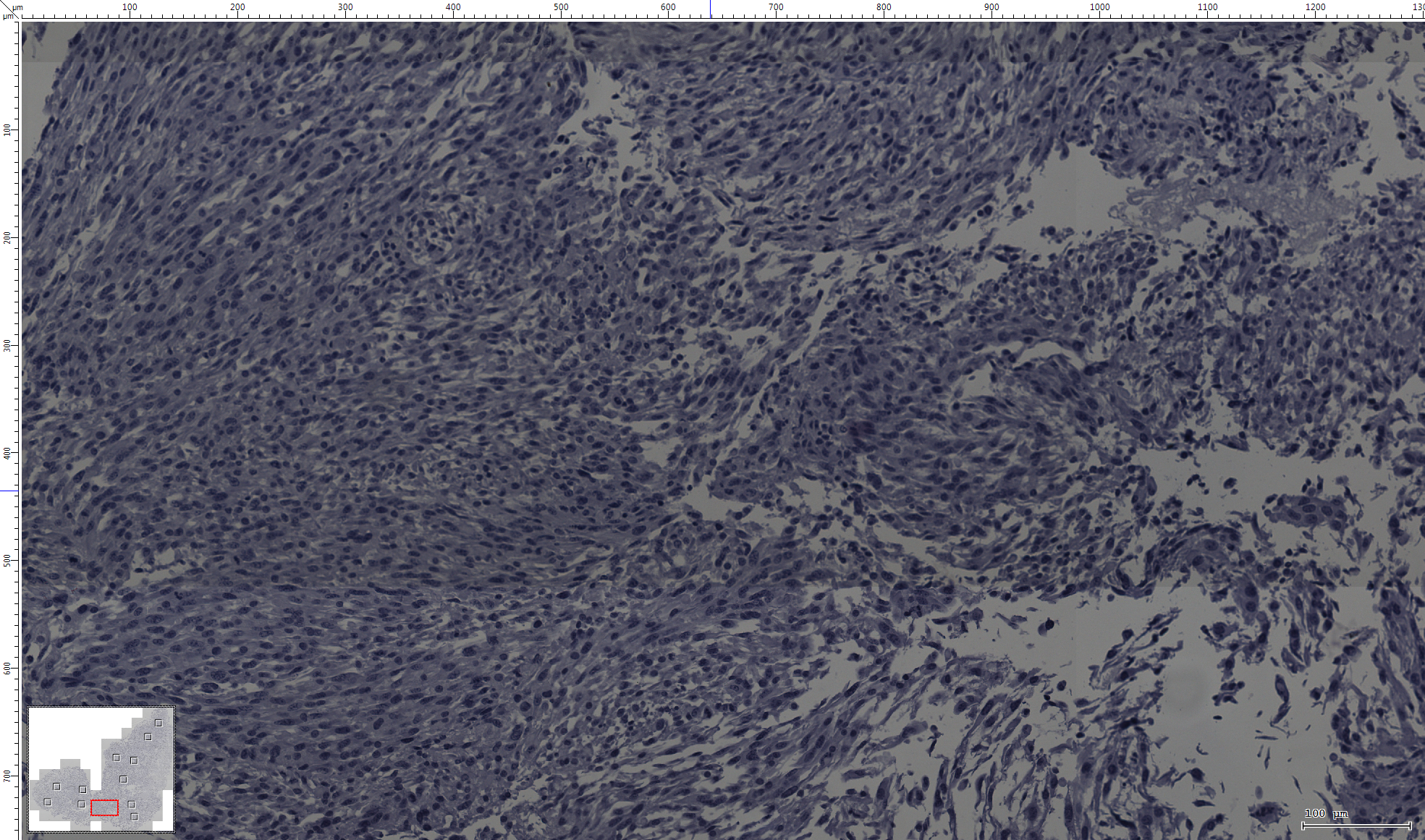

Supplement: Supplementary file 3 — Source Data for Expanded View and Appendix [file EMMM-12-e10941-s010.zip › Fig EV3/PT K21.TIFF]

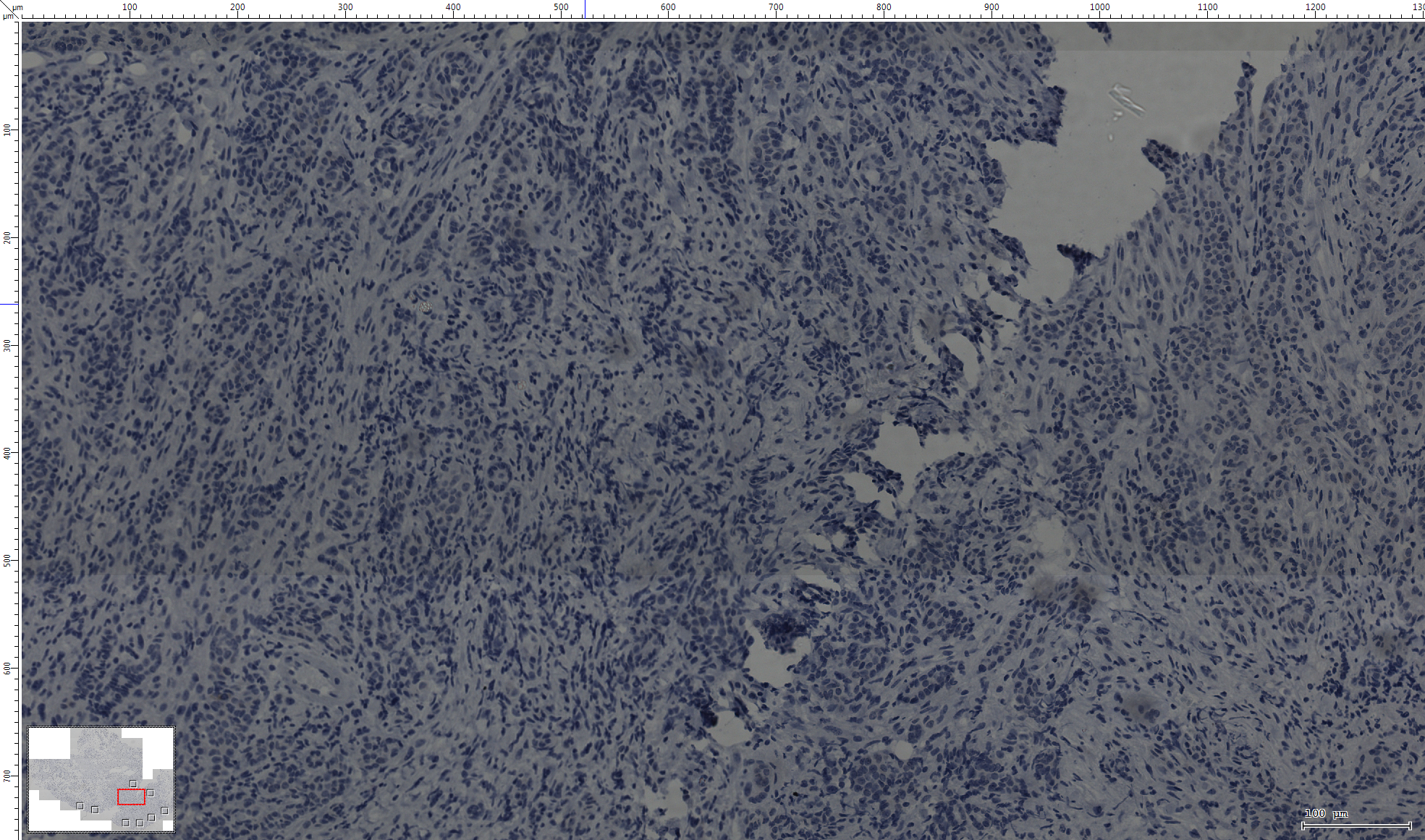

Supplement: Supplementary file 3 — Source Data for Expanded View and Appendix [file EMMM-12-e10941-s010.zip › Fig EV3/PT K20.TIFF]

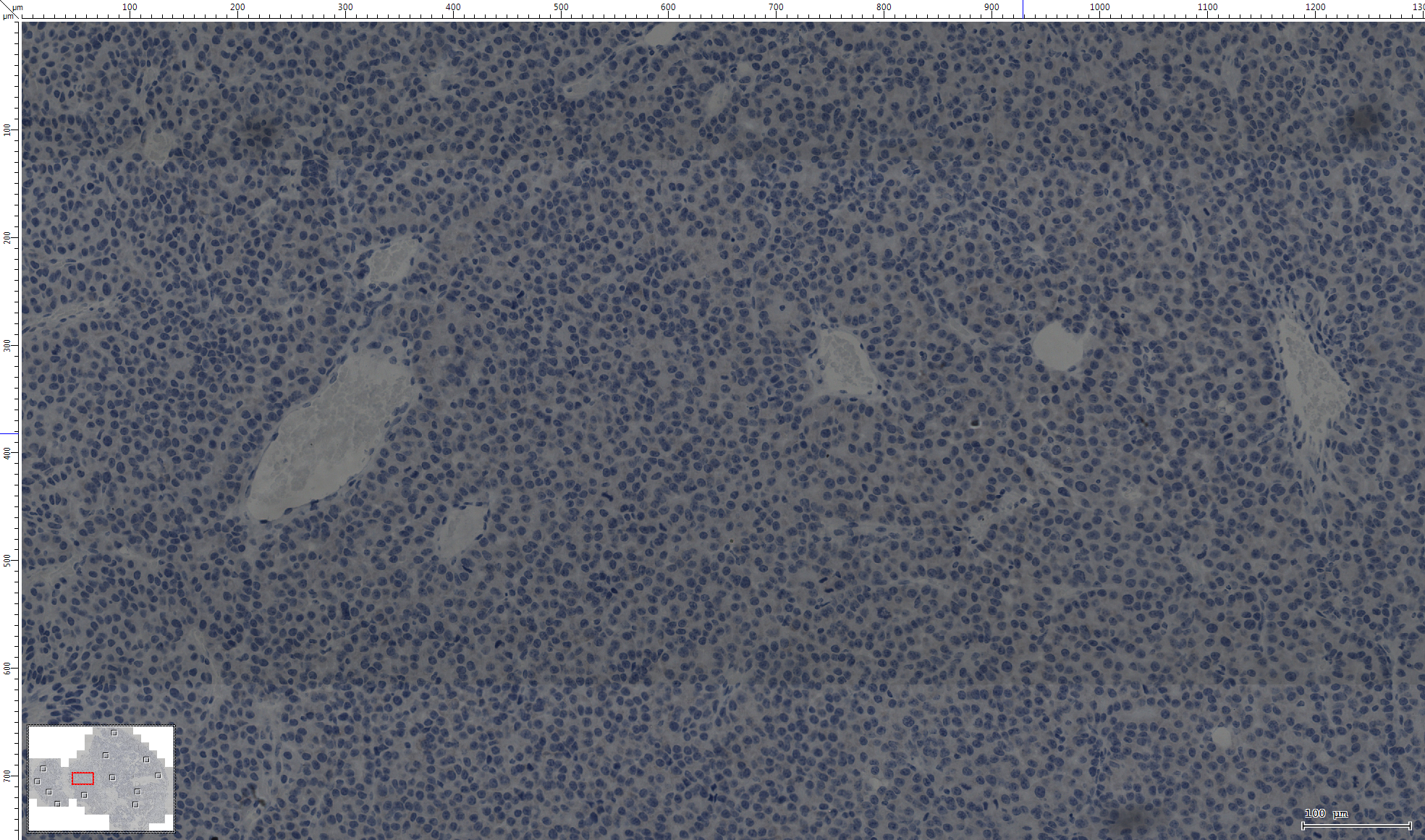

Supplement: Supplementary file 3 — Source Data for Expanded View and Appendix [file EMMM-12-e10941-s010.zip › Fig EV3/PT KM10.TIFF]

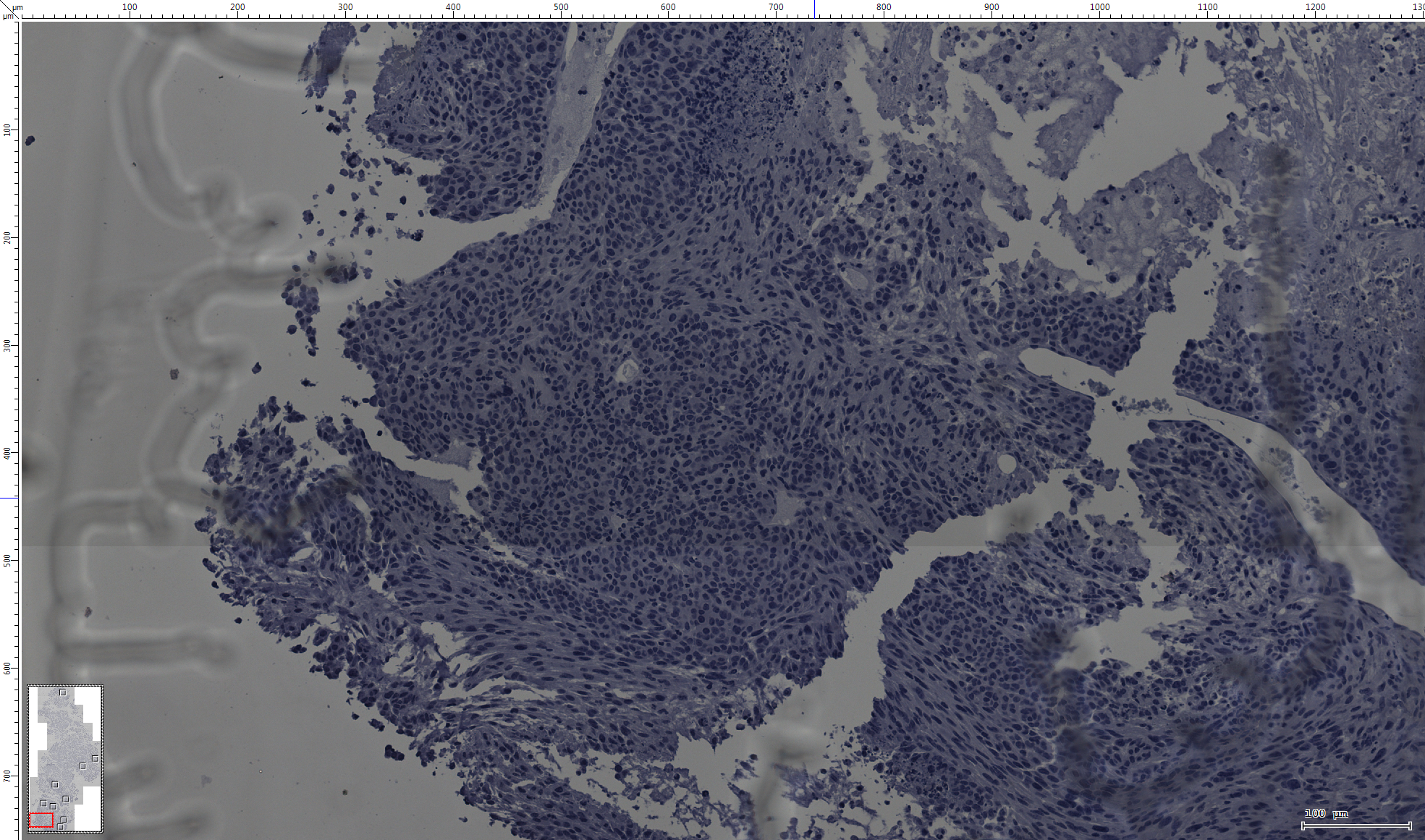

Supplement: Supplementary file 3 — Source Data for Expanded View and Appendix [file EMMM-12-e10941-s010.zip › Fig EV3/PT K3.TIFF]

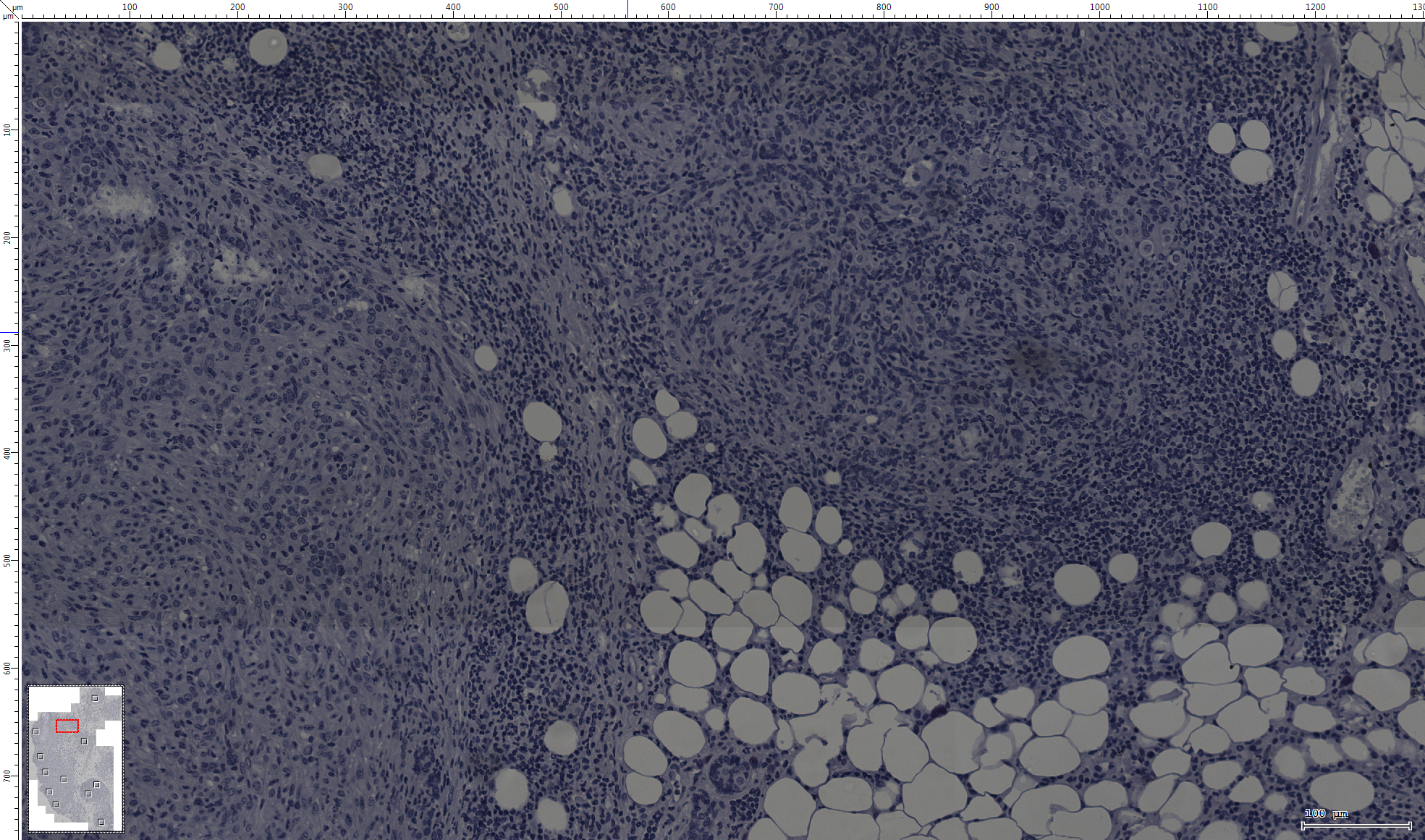

Supplement: Supplementary file 3 — Source Data for Expanded View and Appendix [file EMMM-12-e10941-s010.zip › Fig EV3/PT K16.TIFF]

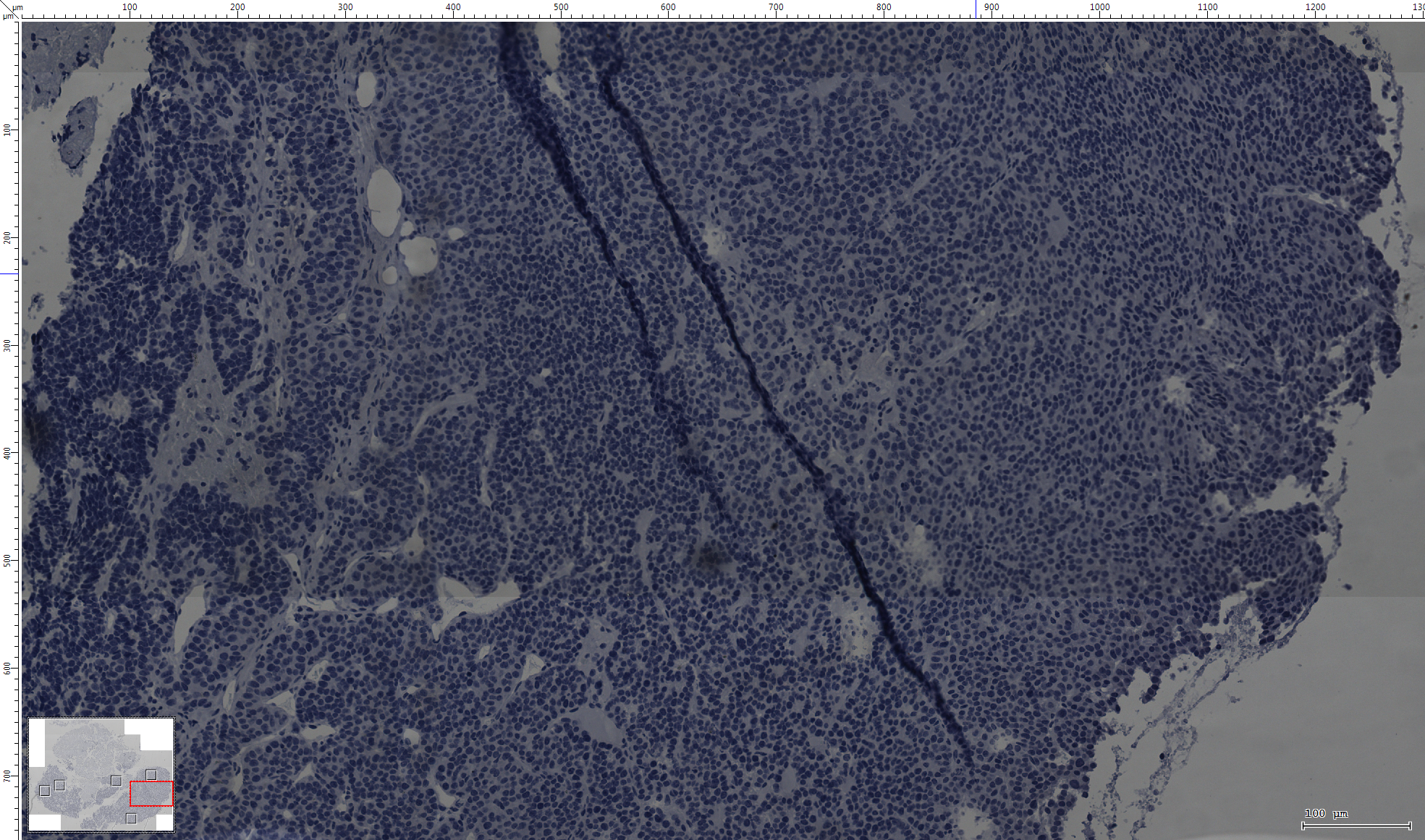

Supplement: Supplementary file 3 — Source Data for Expanded View and Appendix [file EMMM-12-e10941-s010.zip › Fig EV3/PT K11.TIFF]

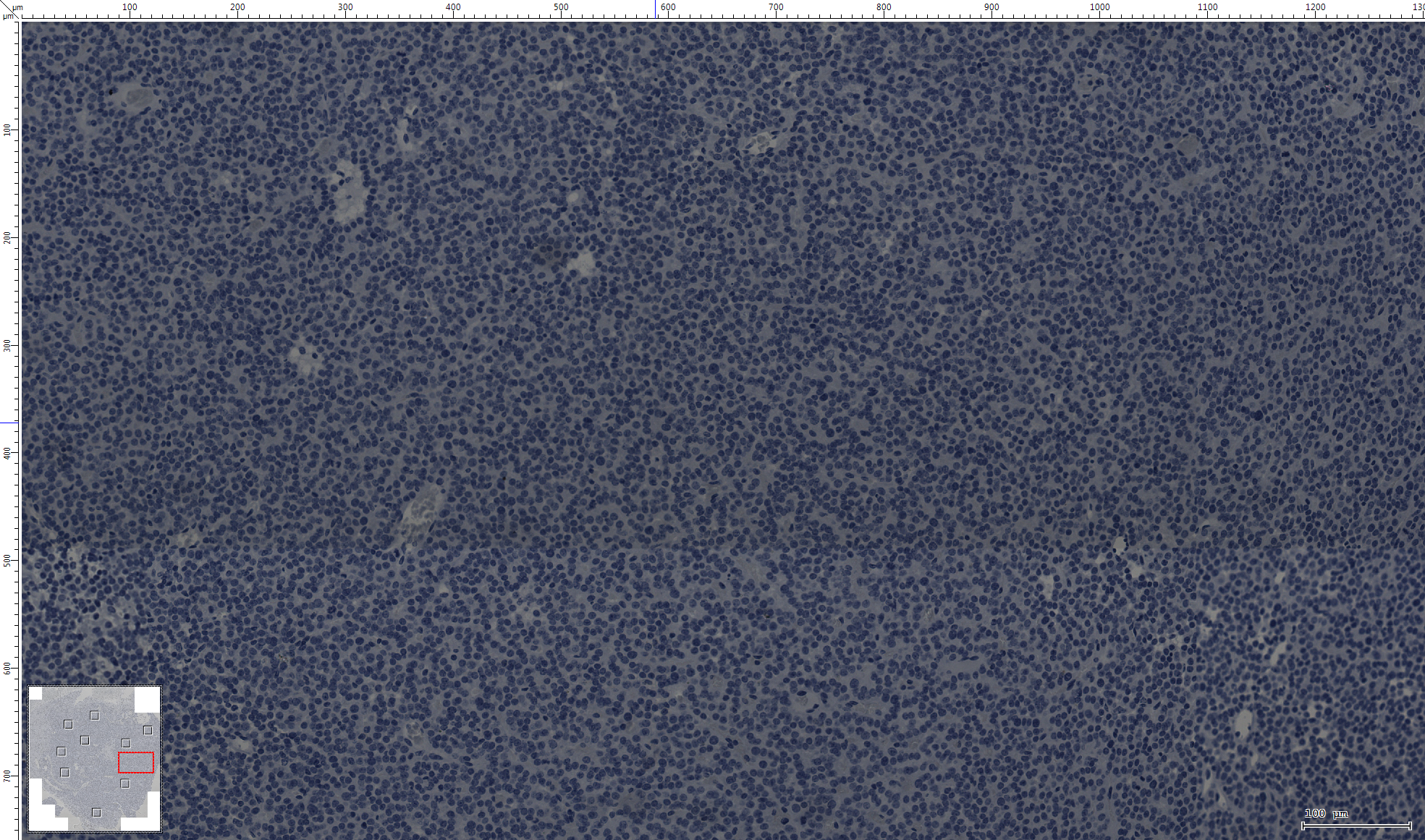

Supplement: Supplementary file 3 — Source Data for Expanded View and Appendix [file EMMM-12-e10941-s010.zip › Fig EV3/PT K4.TIFF]

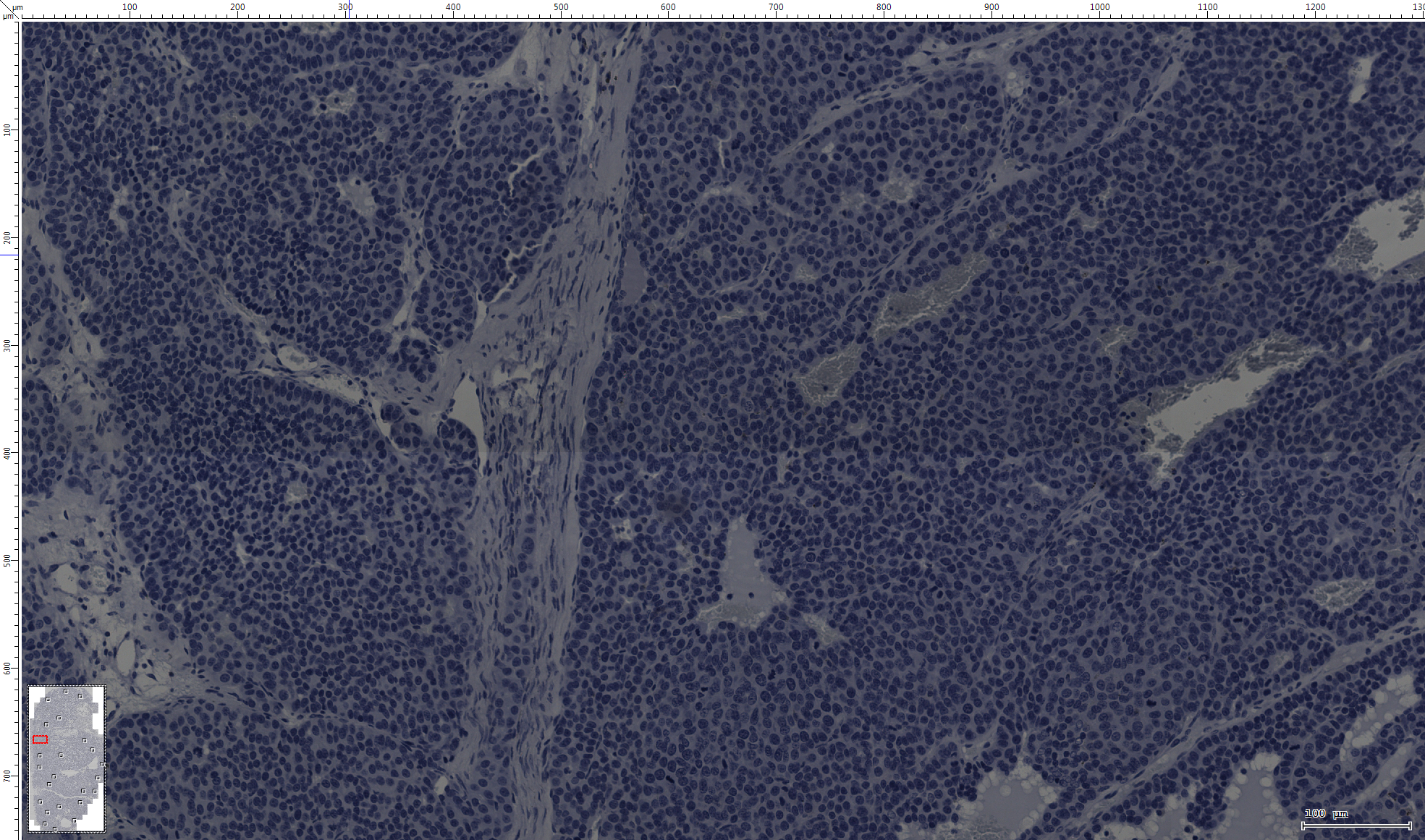

Supplement: Supplementary file 3 — Source Data for Expanded View and Appendix [file EMMM-12-e10941-s010.zip › Fig EV3/PT K5.TIFF]

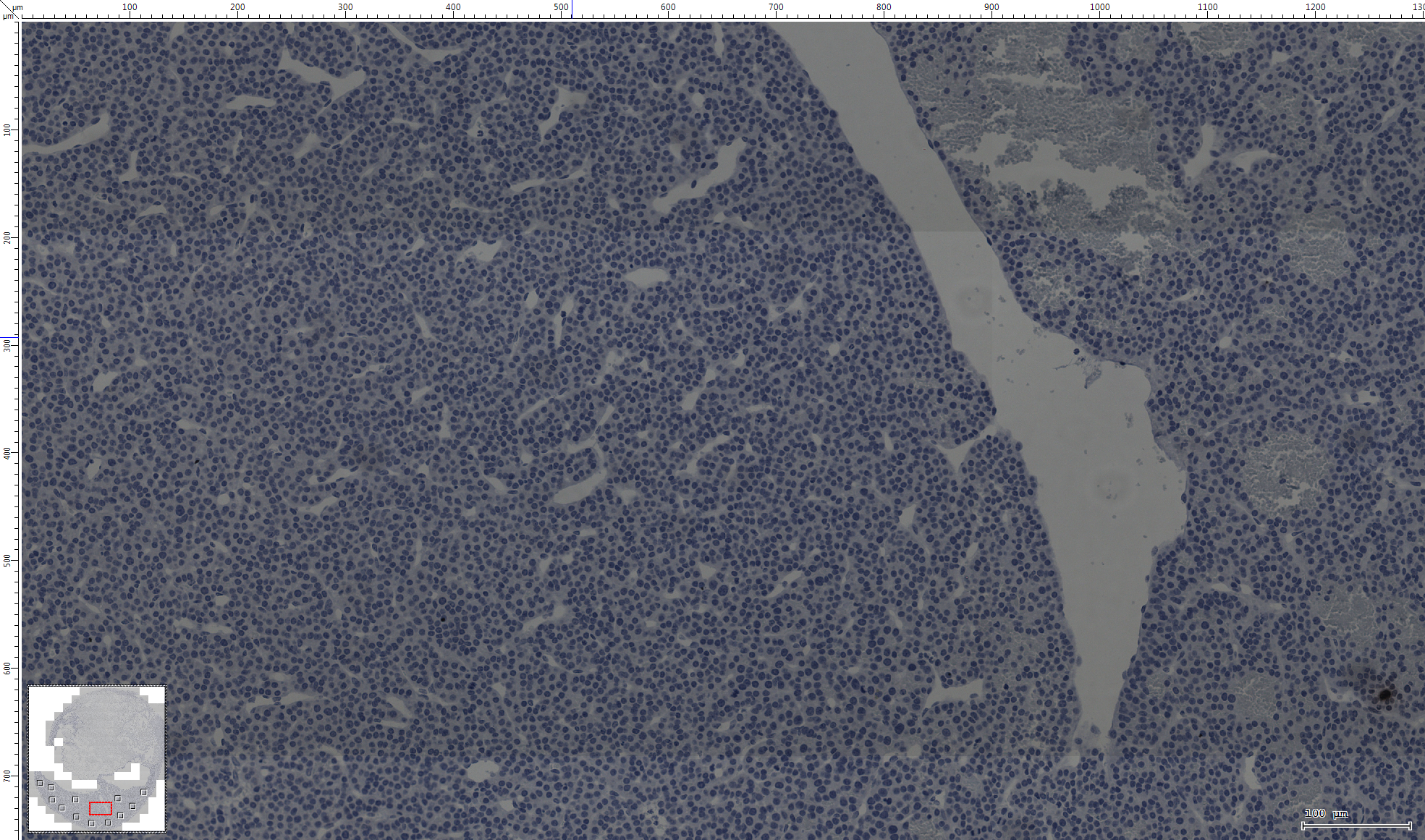

Supplement: Supplementary file 3 — Source Data for Expanded View and Appendix [file EMMM-12-e10941-s010.zip › Fig EV3/PT K10.TIFF]

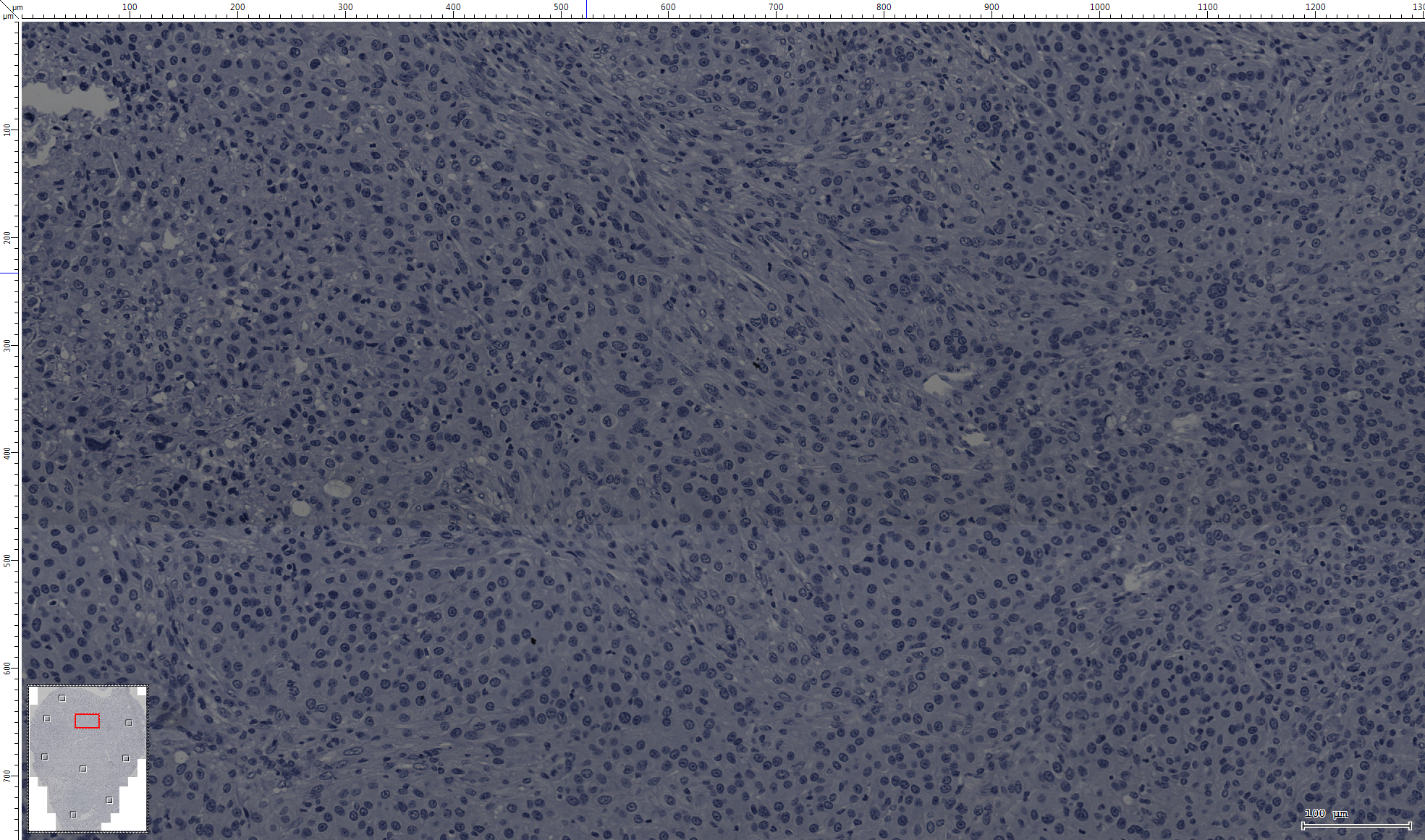

Supplement: Supplementary file 3 — Source Data for Expanded View and Appendix [file EMMM-12-e10941-s010.zip › Fig EV3/PT K9.TIFF]

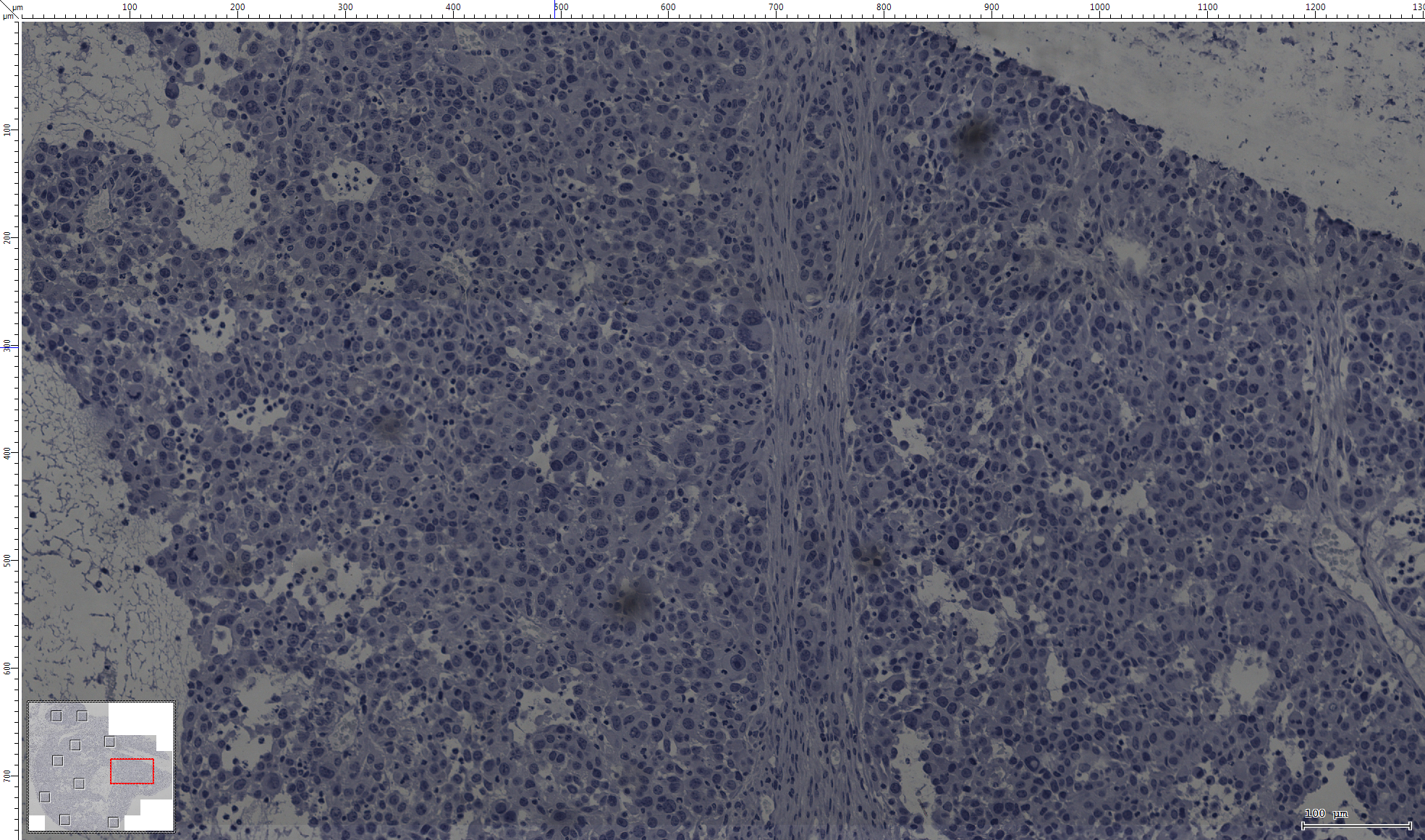

Supplement: Supplementary file 3 — Source Data for Expanded View and Appendix [file EMMM-12-e10941-s010.zip › Fig EV3/PT KM6.TIFF]

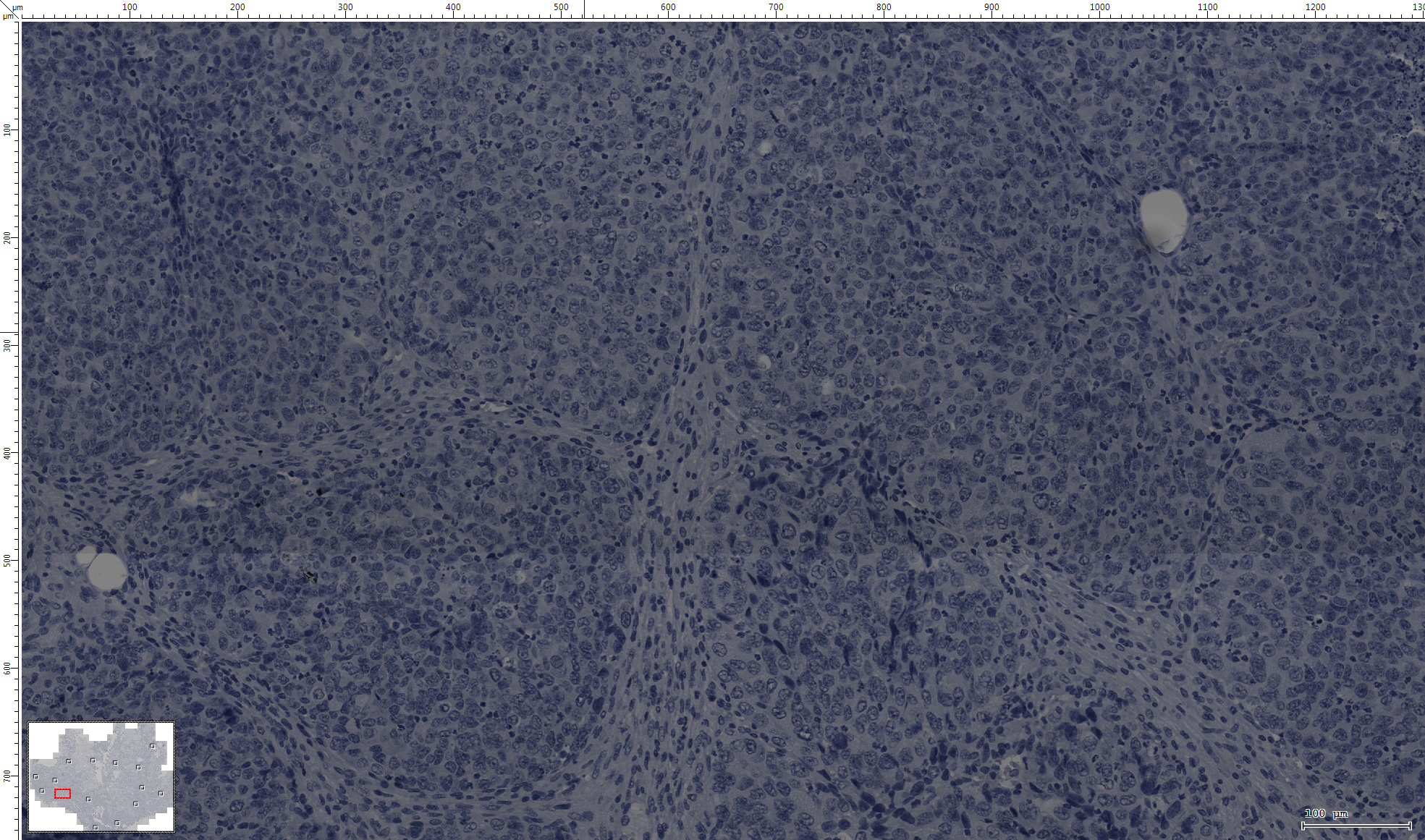

Supplement: Supplementary file 3 — Source Data for Expanded View and Appendix [file EMMM-12-e10941-s010.zip › Fig EV3/PT K25.TIFF]

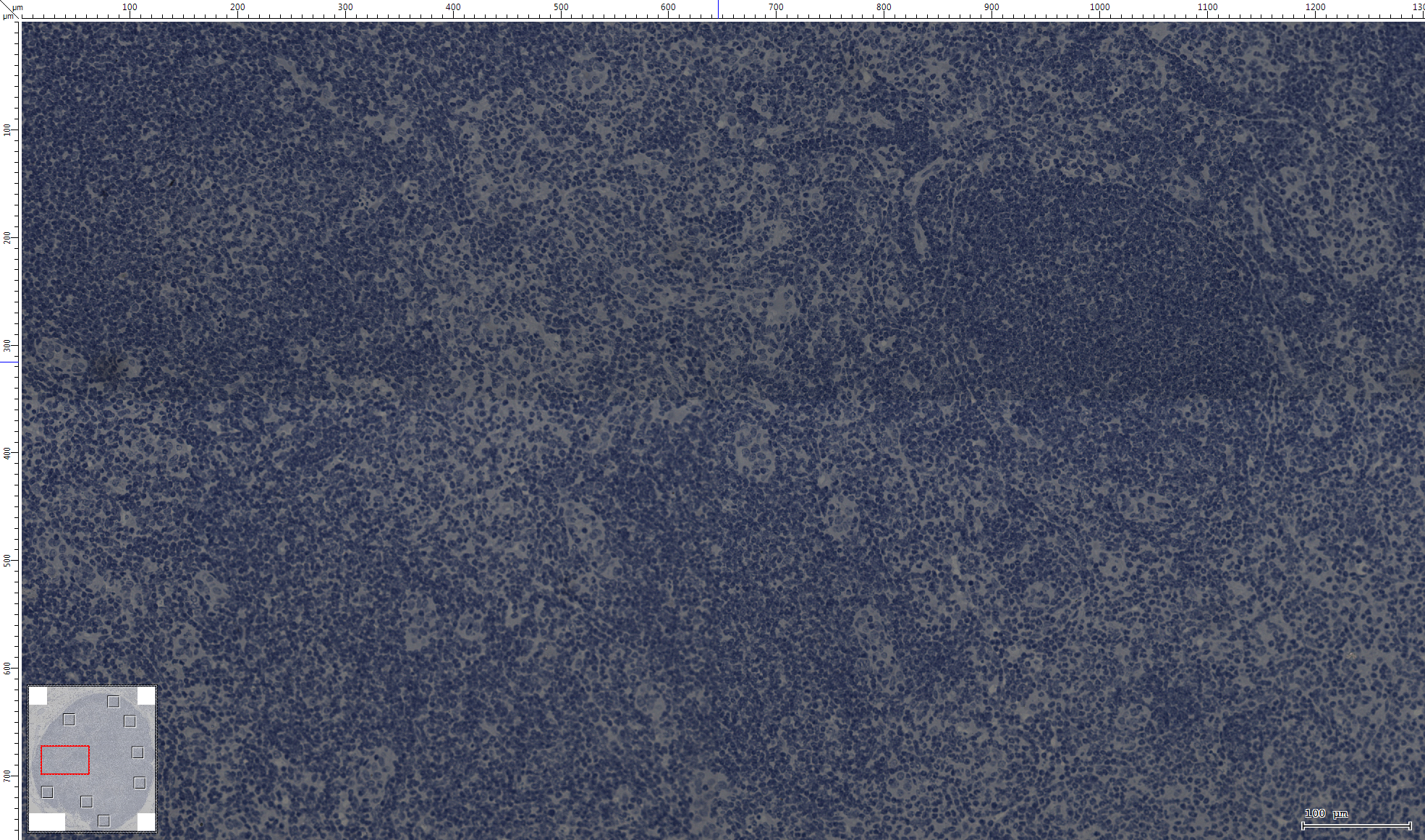

Supplement: Supplementary file 3 — Source Data for Expanded View and Appendix [file EMMM-12-e10941-s010.zip › Fig EV3/PT K6.TIFF]

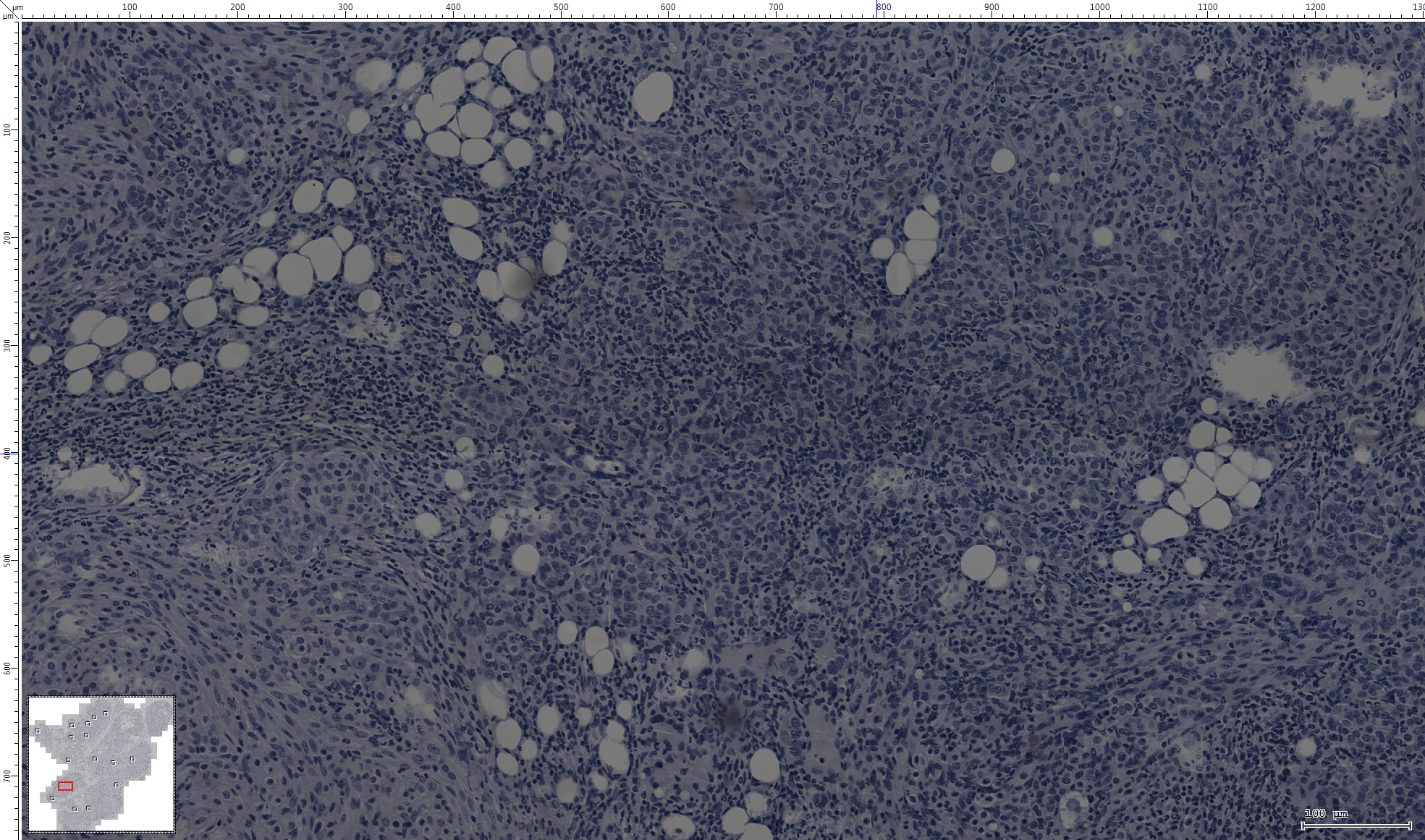

Supplement: Supplementary file 3 — Source Data for Expanded View and Appendix [file EMMM-12-e10941-s010.zip › Fig EV3/PT K13.TIFF]

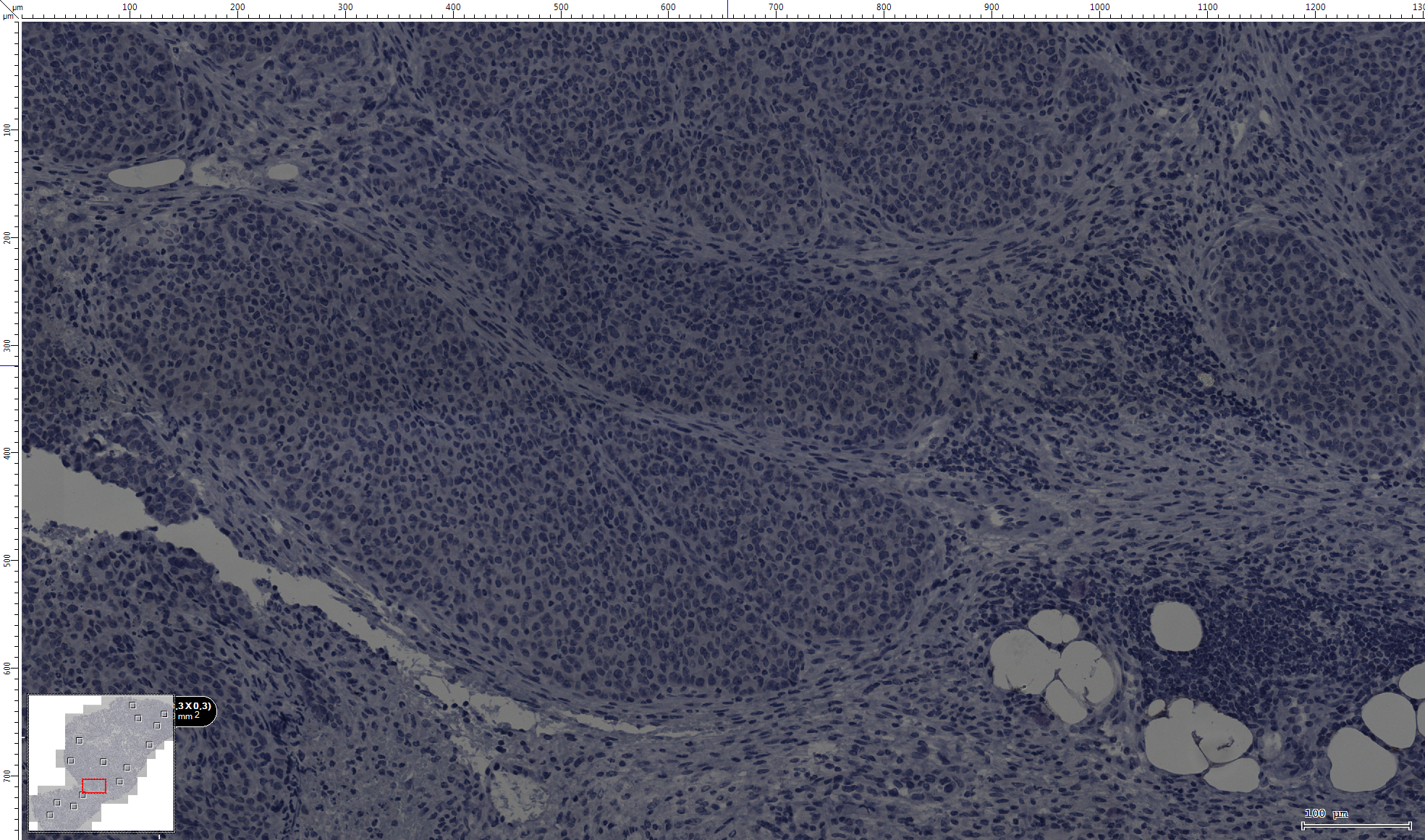

Supplement: Supplementary file 3 — Source Data for Expanded View and Appendix [file EMMM-12-e10941-s010.zip › Fig EV3/PT KM9.TIFF]

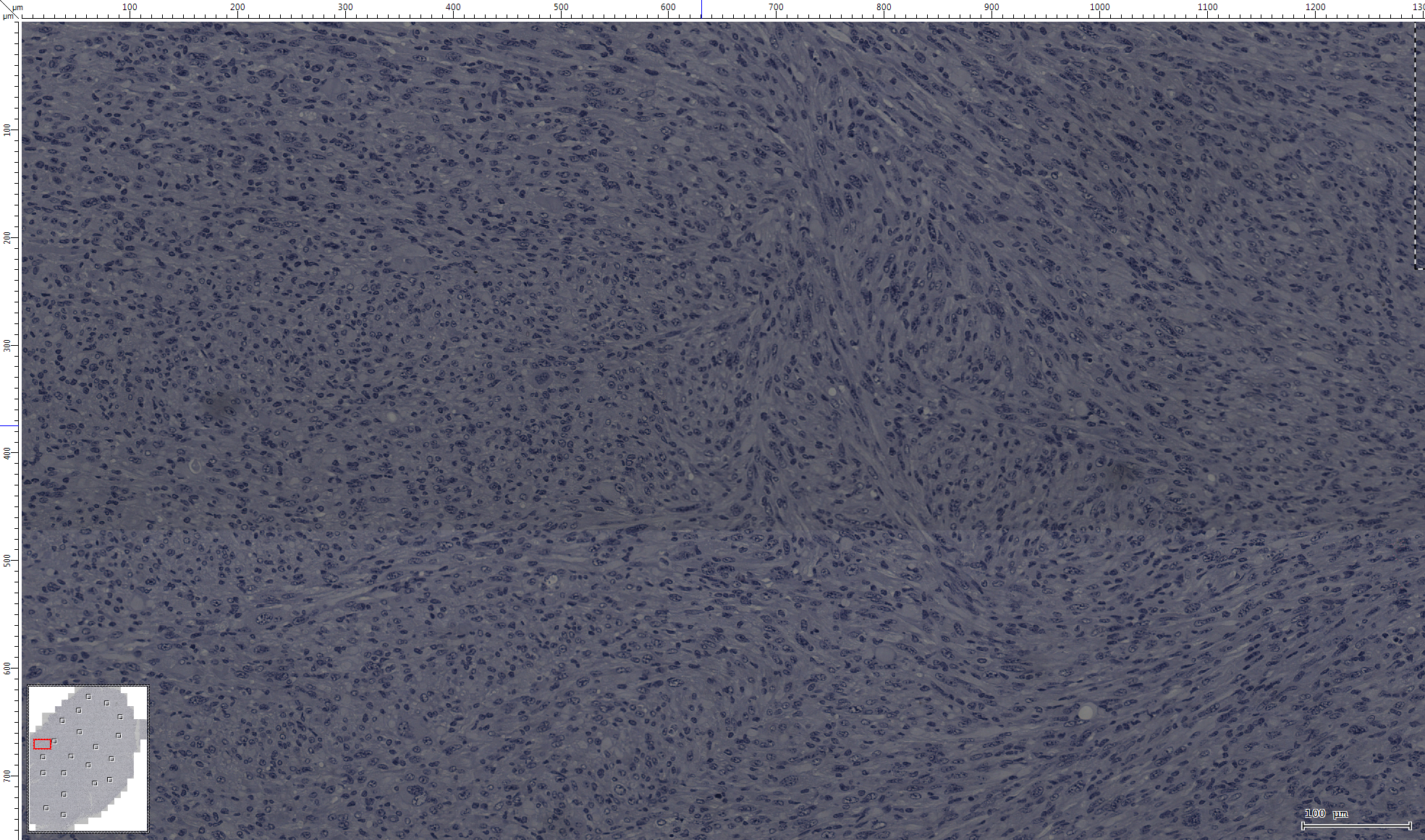

Supplement: Supplementary file 3 — Source Data for Expanded View and Appendix [file EMMM-12-e10941-s010.zip › Fig EV3/PT K12.TIFF]

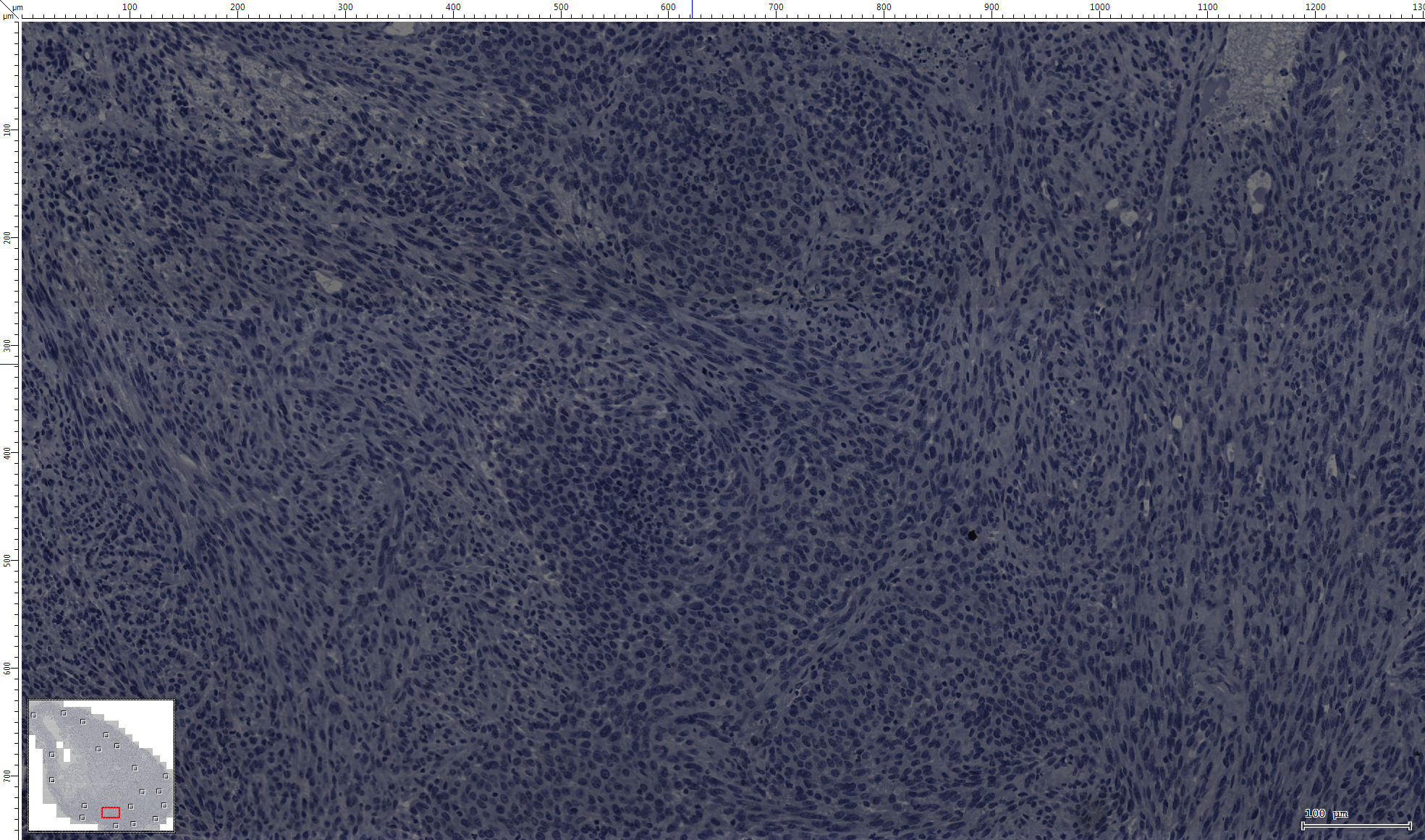

Supplement: Supplementary file 3 — Source Data for Expanded View and Appendix [file EMMM-12-e10941-s010.zip › Fig EV3/PT KM4.TIFF]

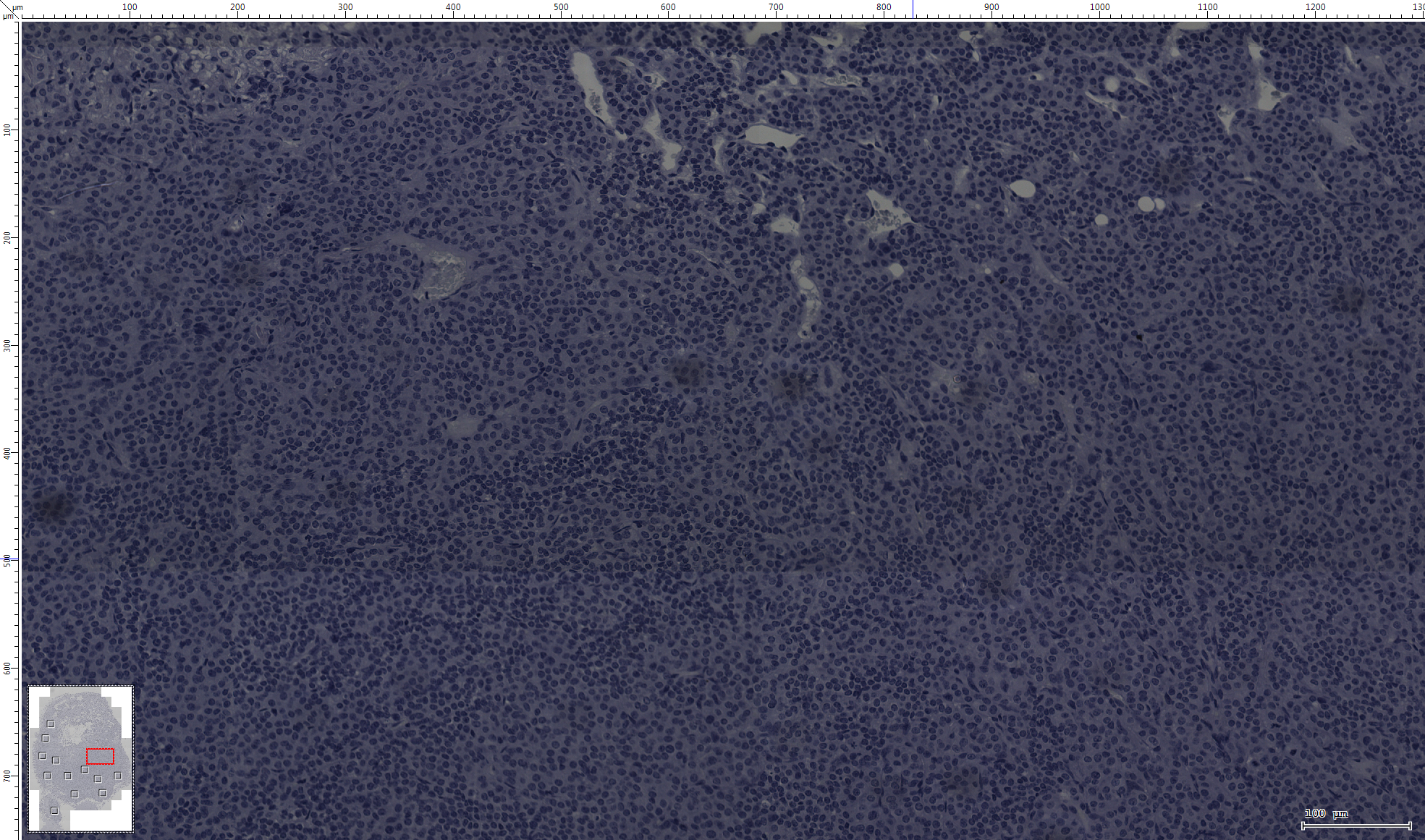

Supplement: Supplementary file 3 — Source Data for Expanded View and Appendix [file EMMM-12-e10941-s010.zip › Fig EV3/PT K24.TIFF]

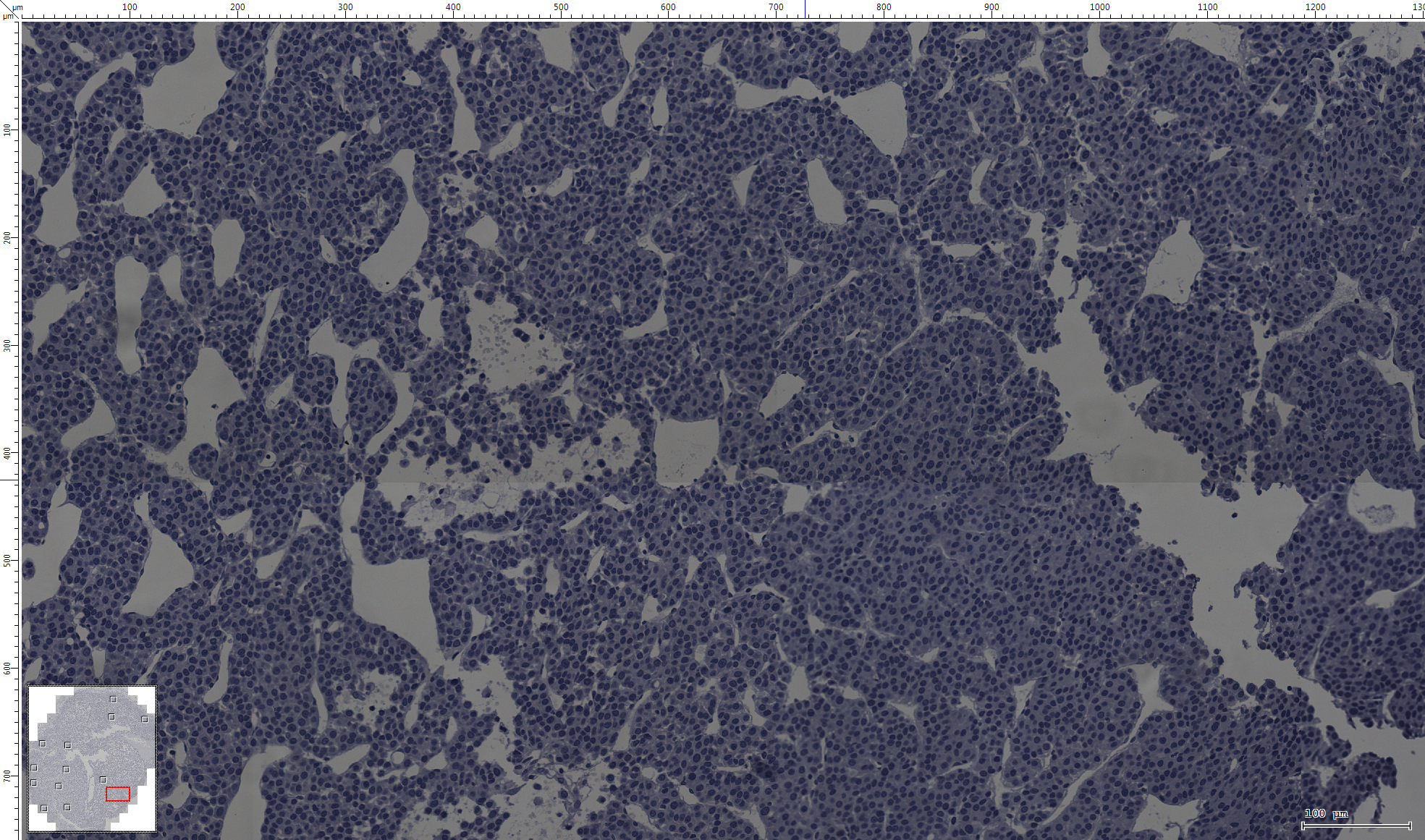

Supplement: Supplementary file 3 — Source Data for Expanded View and Appendix [file EMMM-12-e10941-s010.zip › Fig EV3/PT K15.TIFF]

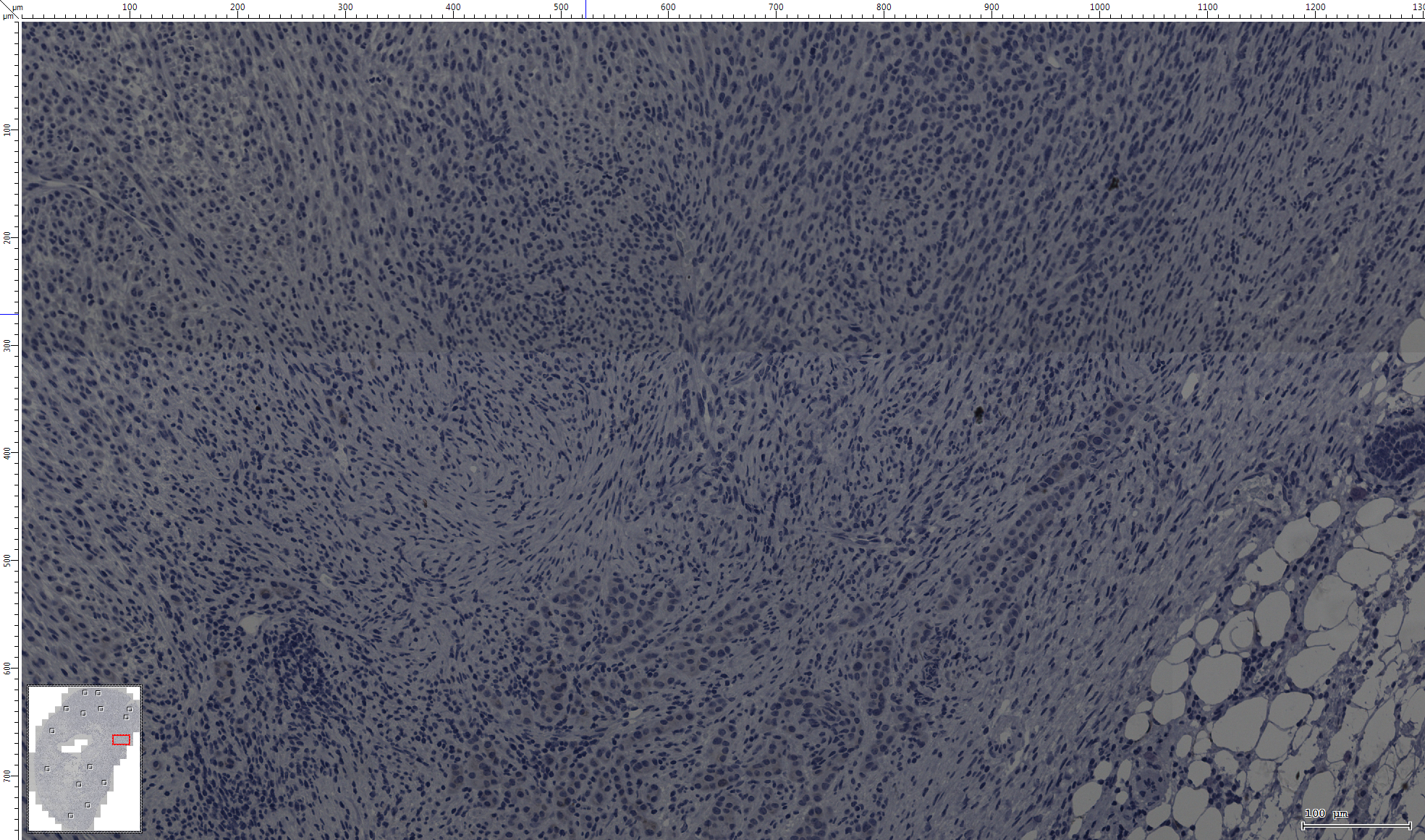

Supplement: Supplementary file 3 — Source Data for Expanded View and Appendix [file EMMM-12-e10941-s010.zip › Fig EV3/PT K19.TIFF]

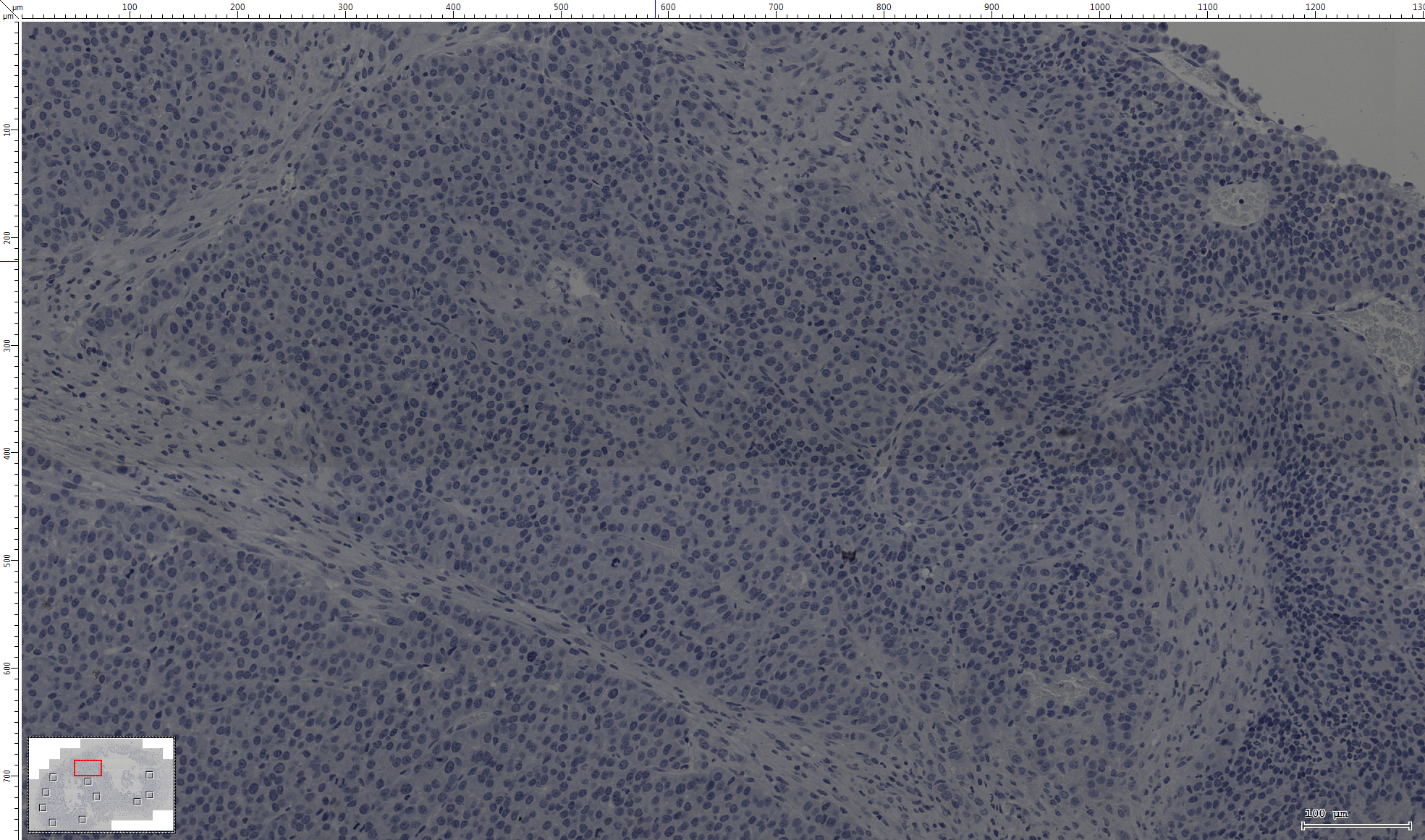

Supplement: Supplementary file 3 — Source Data for Expanded View and Appendix [file EMMM-12-e10941-s010.zip › Fig EV3/PT K23.TIFF]

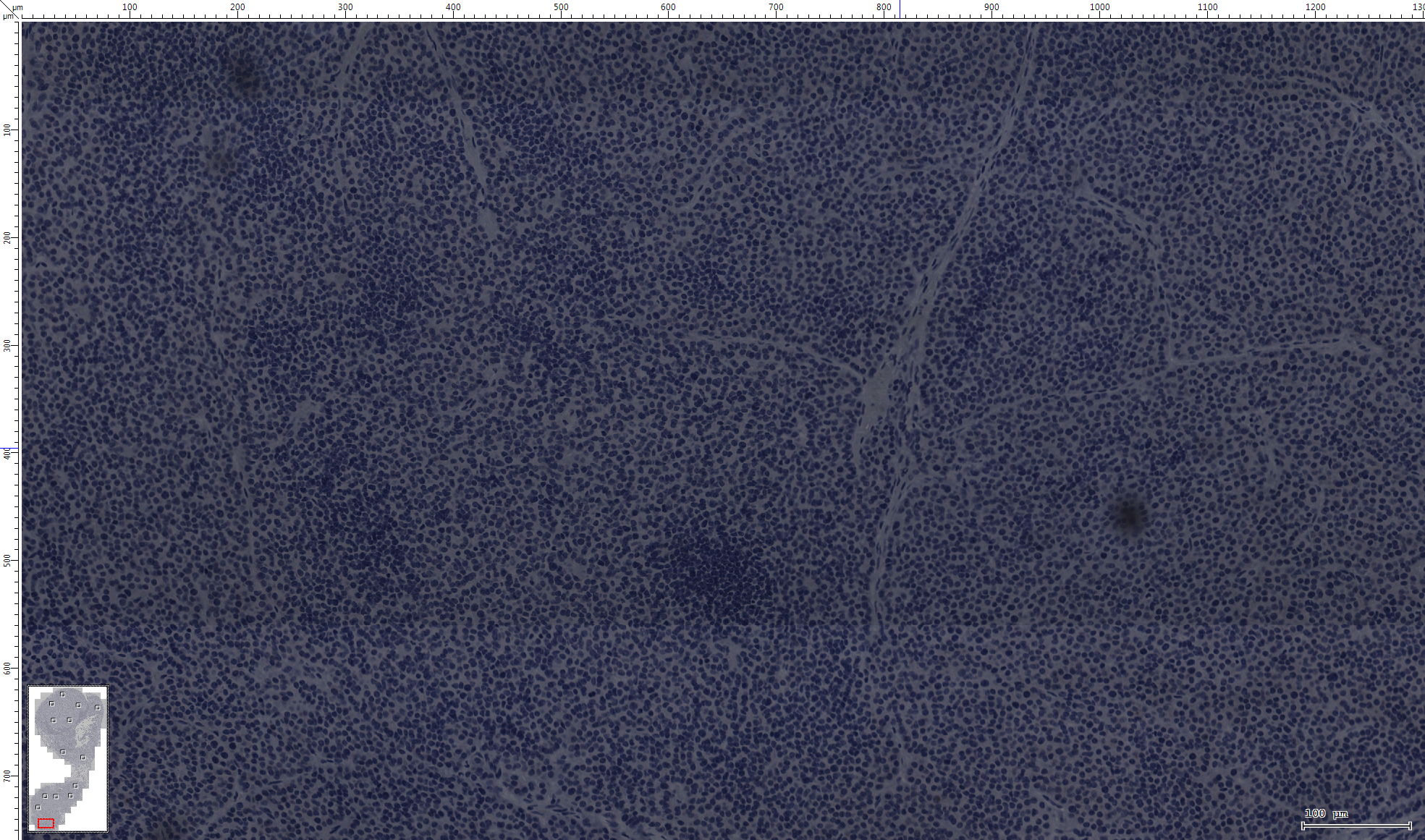

Supplement: Supplementary file 3 — Source Data for Expanded View and Appendix [file EMMM-12-e10941-s010.zip › Fig EV3/PT KM3.TIFF]

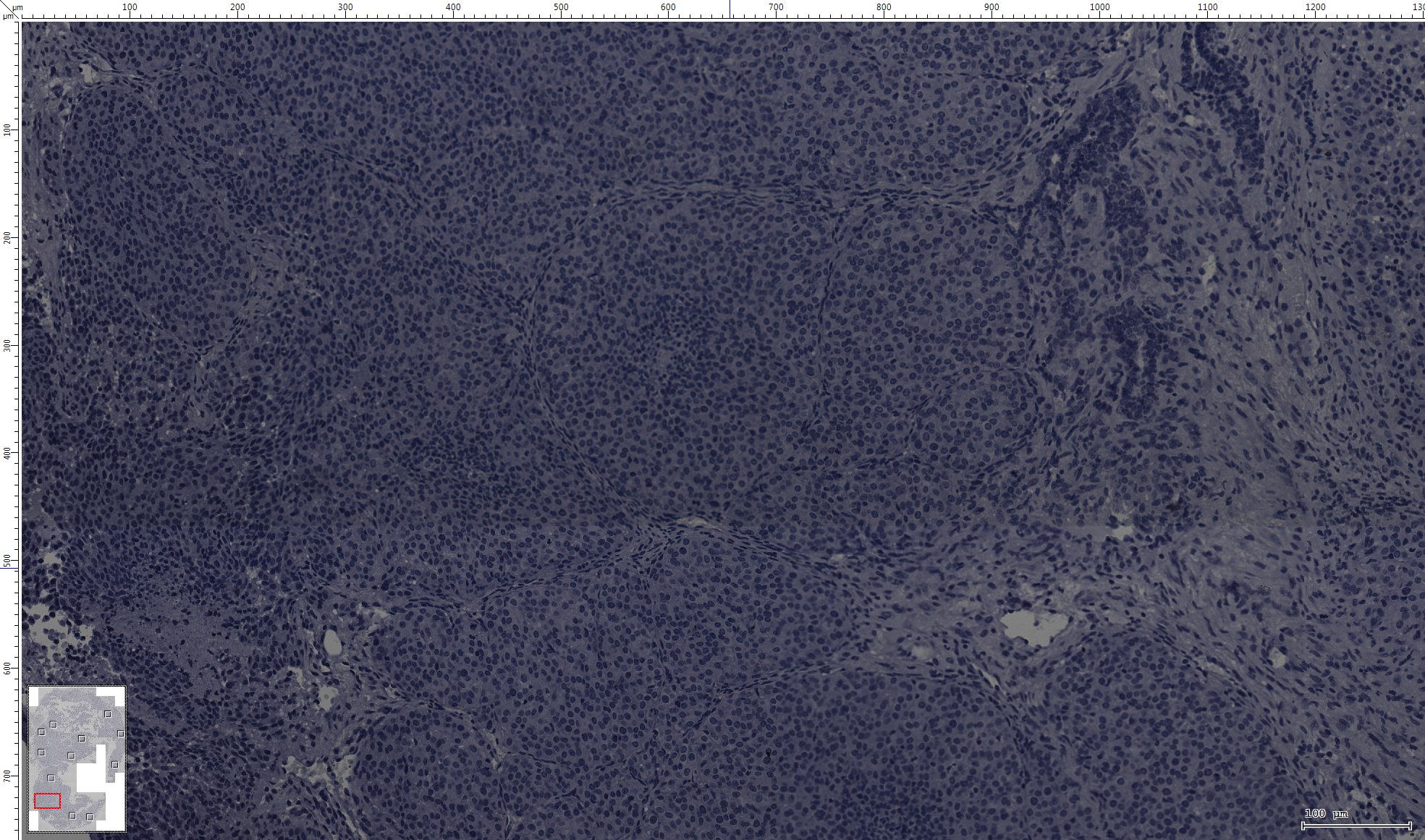

Supplement: Supplementary file 3 — Source Data for Expanded View and Appendix [file EMMM-12-e10941-s010.zip › Fig EV3/PT KM2.TIFF]

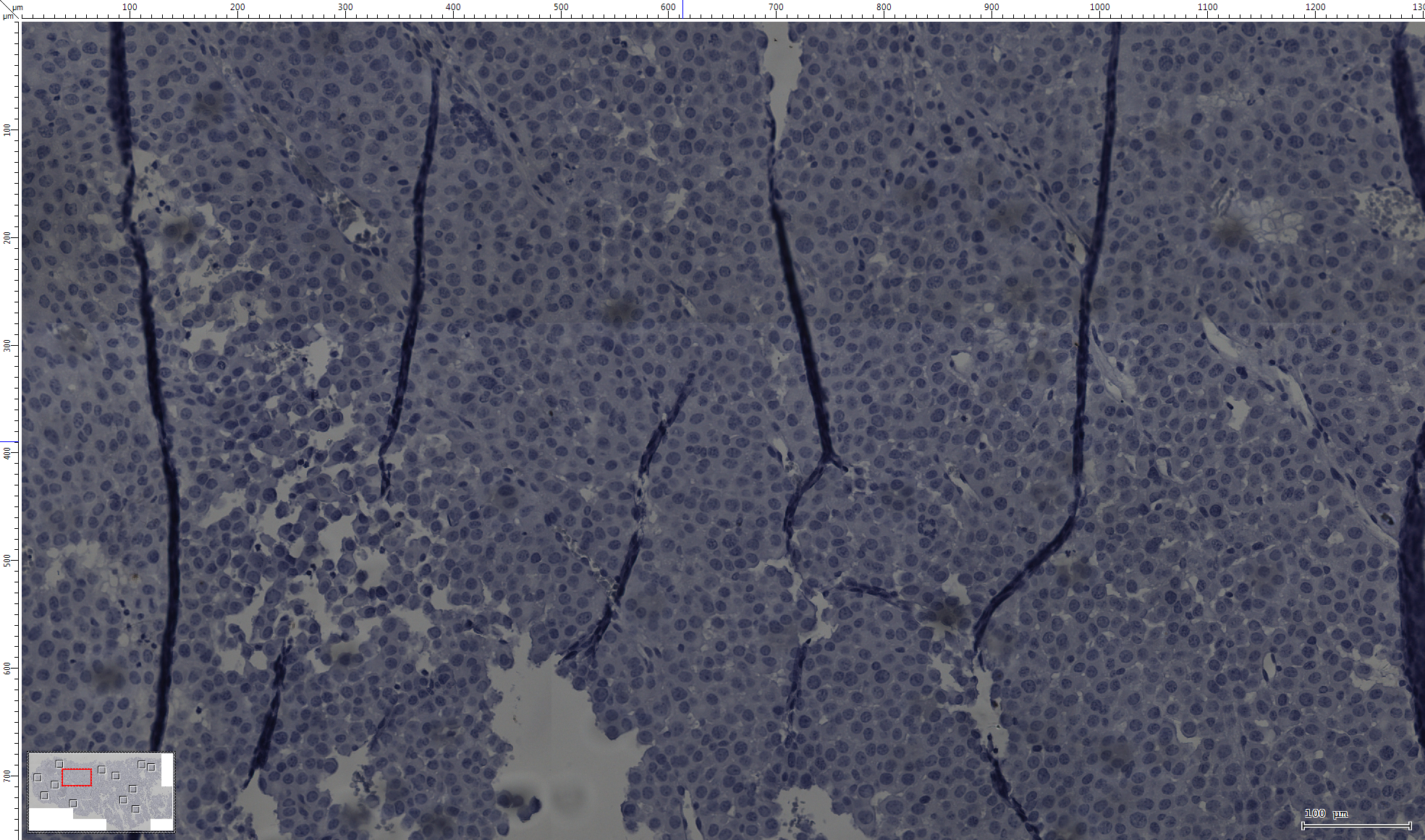

Supplement: Supplementary file 3 — Source Data for Expanded View and Appendix [file EMMM-12-e10941-s010.zip › Fig EV3/PT KM12.TIFF]

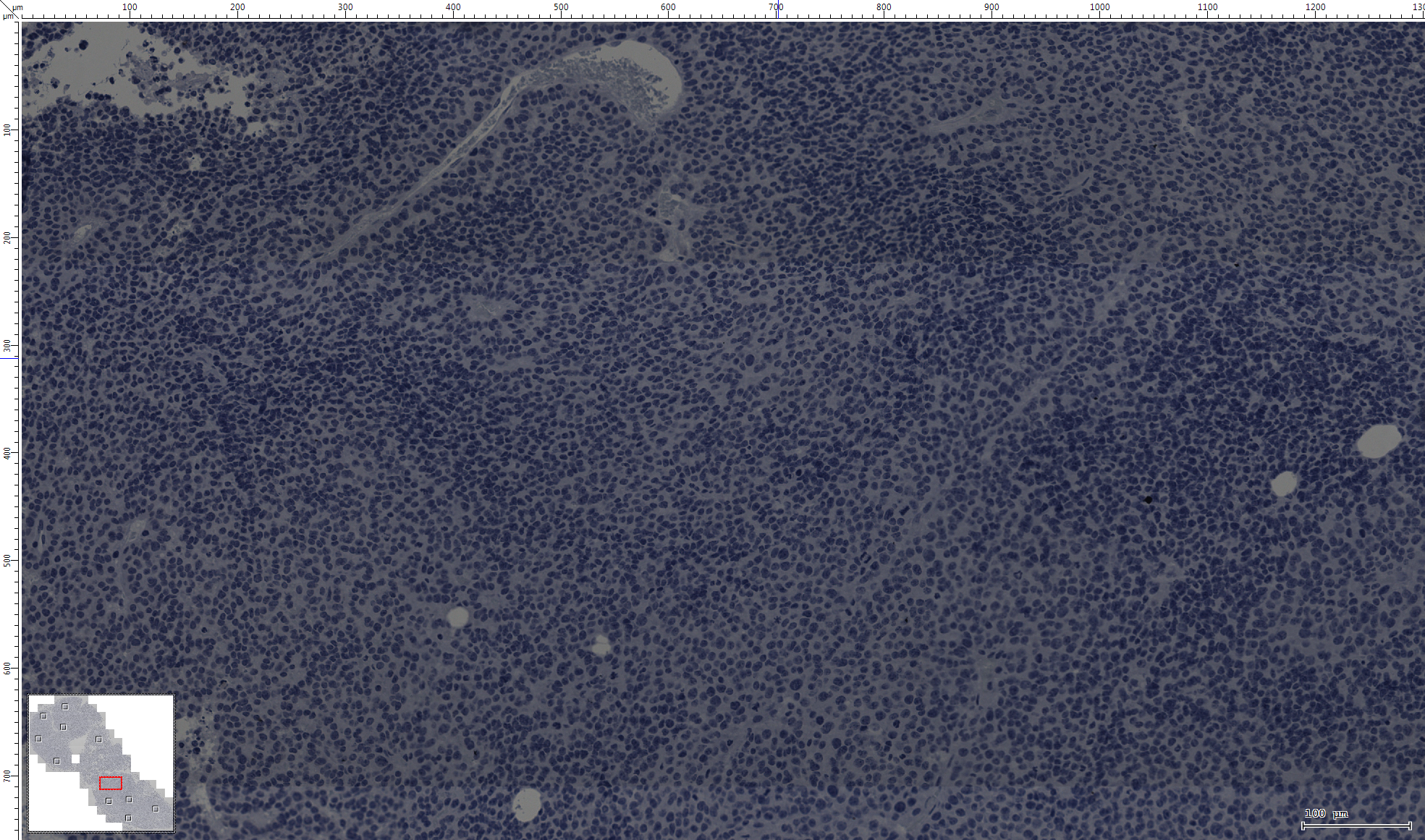

Supplement: Supplementary file 3 — Source Data for Expanded View and Appendix [file EMMM-12-e10941-s010.zip › Fig EV3/PT K22.TIFF]

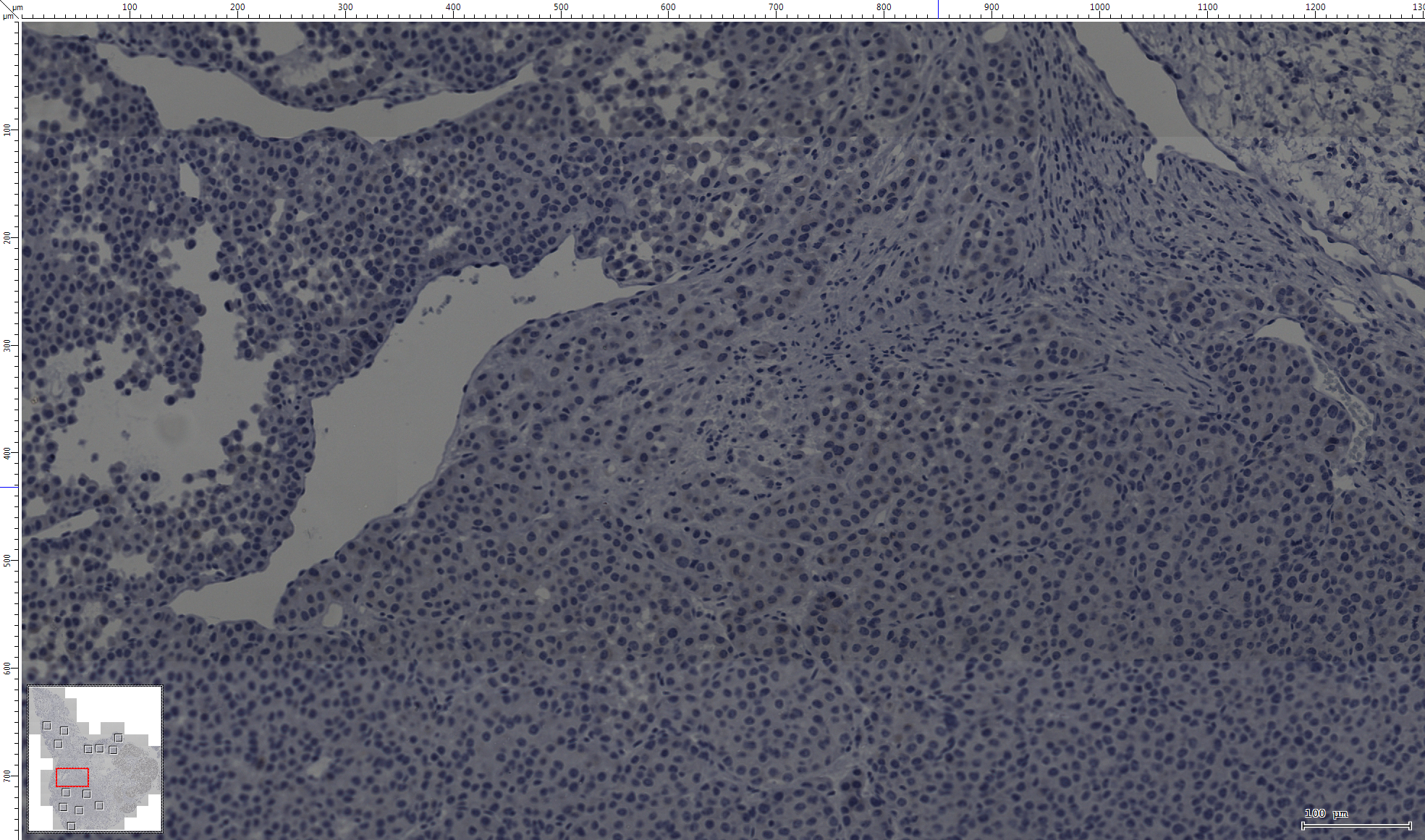

Supplement: Supplementary file 3 — Source Data for Expanded View and Appendix [file EMMM-12-e10941-s010.zip › Fig EV3/PT K18.TIFF]

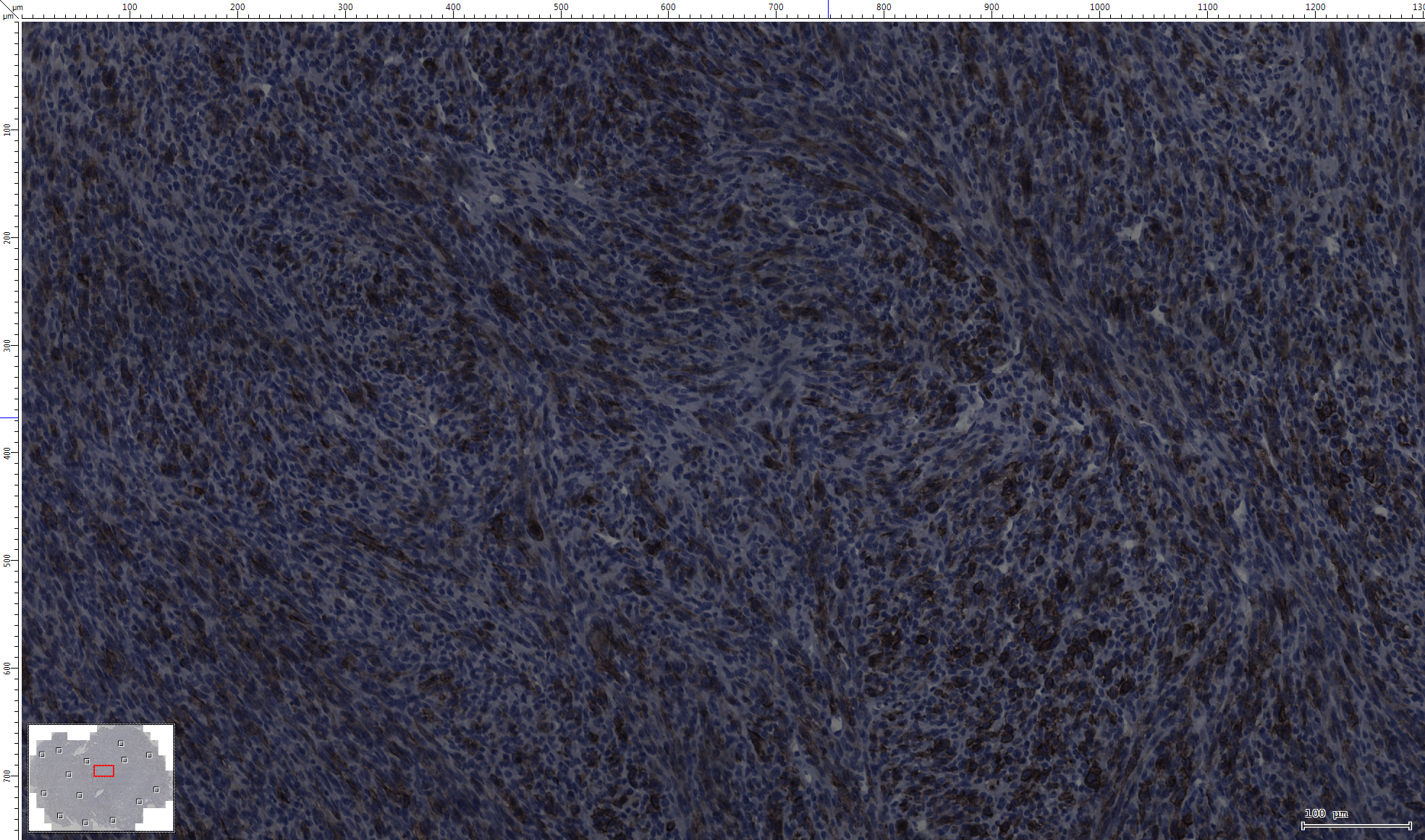

Supplement: Supplementary file 3 — Source Data for Expanded View and Appendix [file EMMM-12-e10941-s010.zip › Fig EV3/KM15.TIFF]

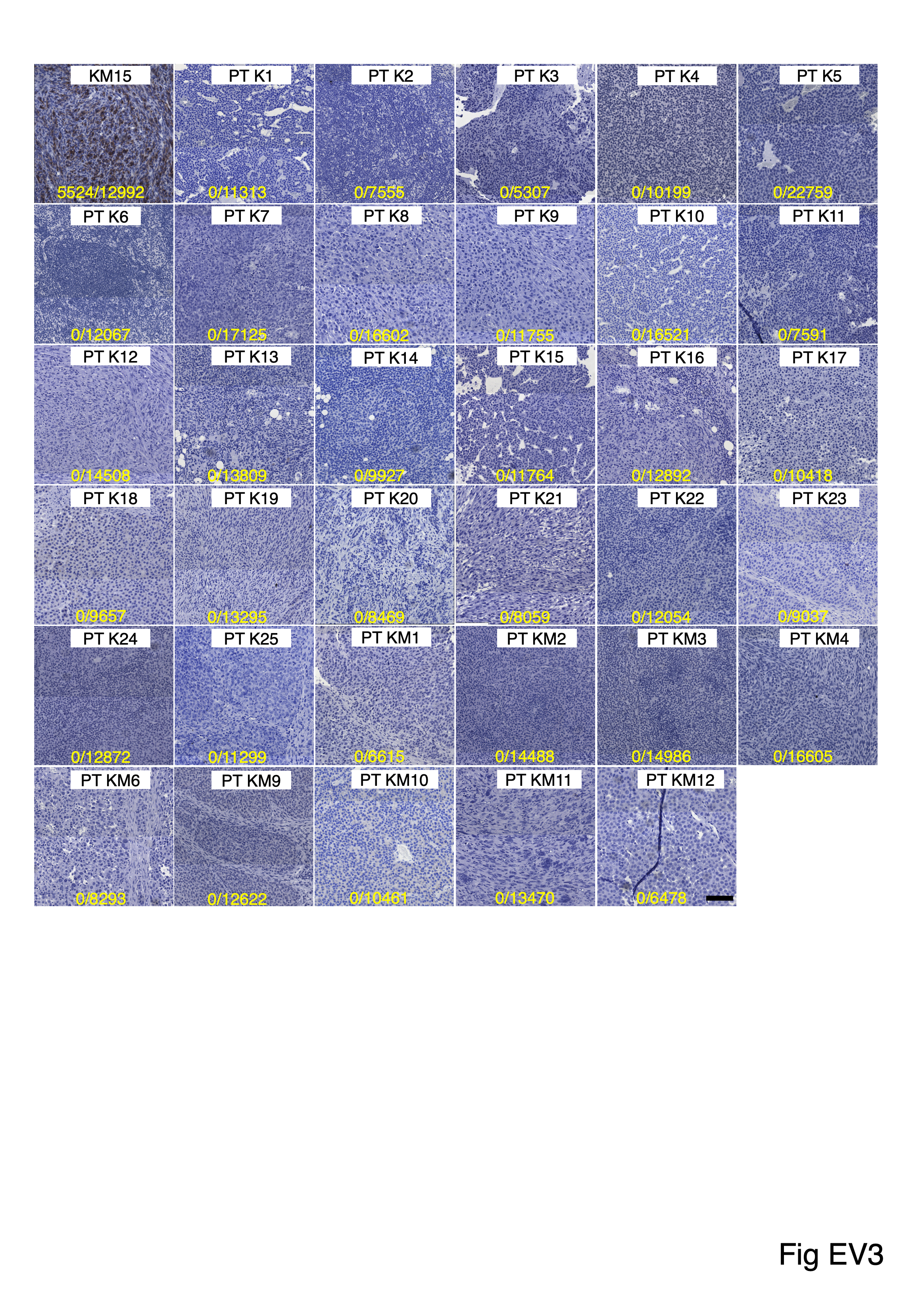

Supplement: Supplementary file 3 — Source Data for Expanded View and Appendix [file EMMM-12-e10941-s010.zip › Fig EV3/Fig EV3.tiff]

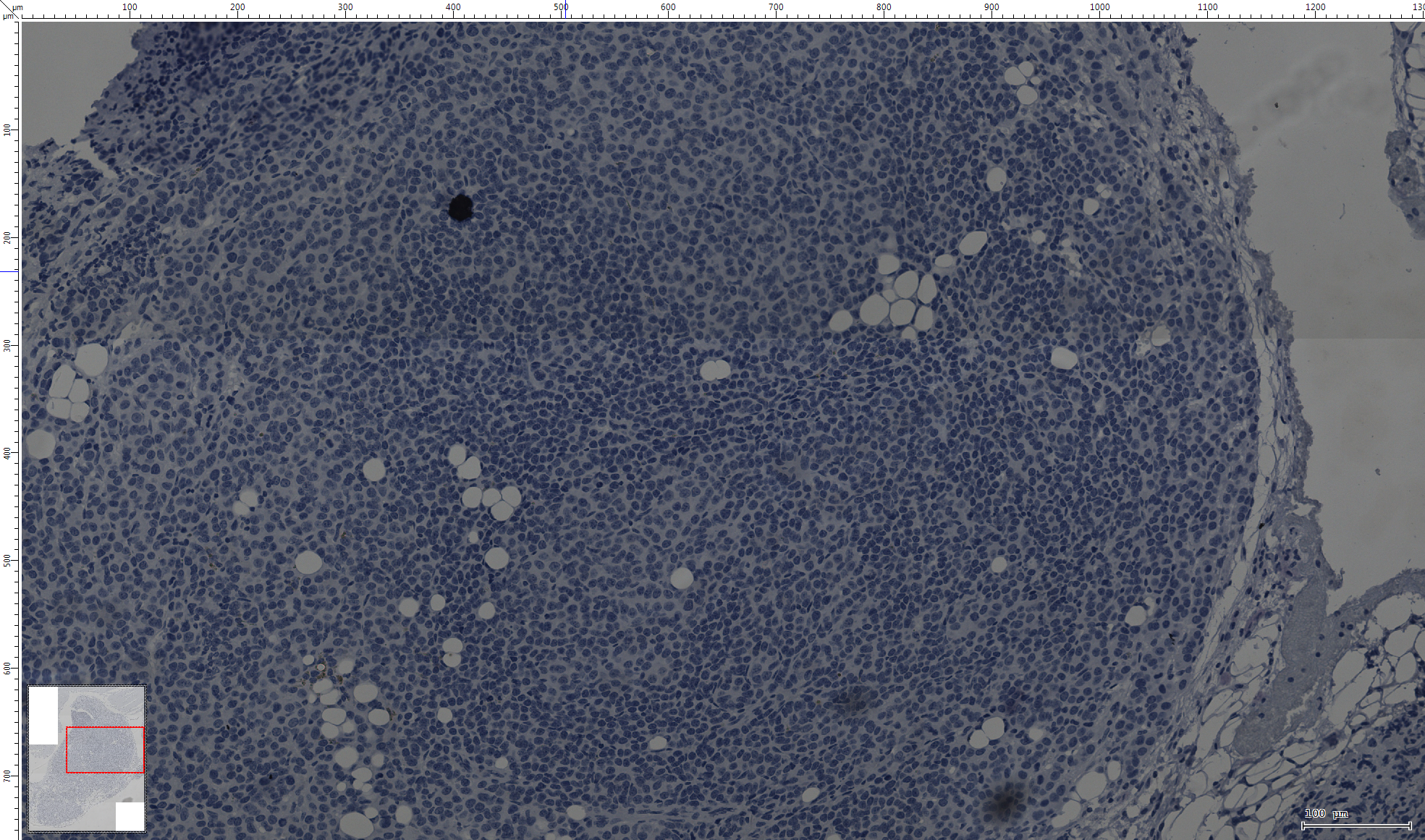

Supplement: Supplementary file 3 — Source Data for Expanded View and Appendix [file EMMM-12-e10941-s010.zip › Fig EV3/PT K14.TIFF]

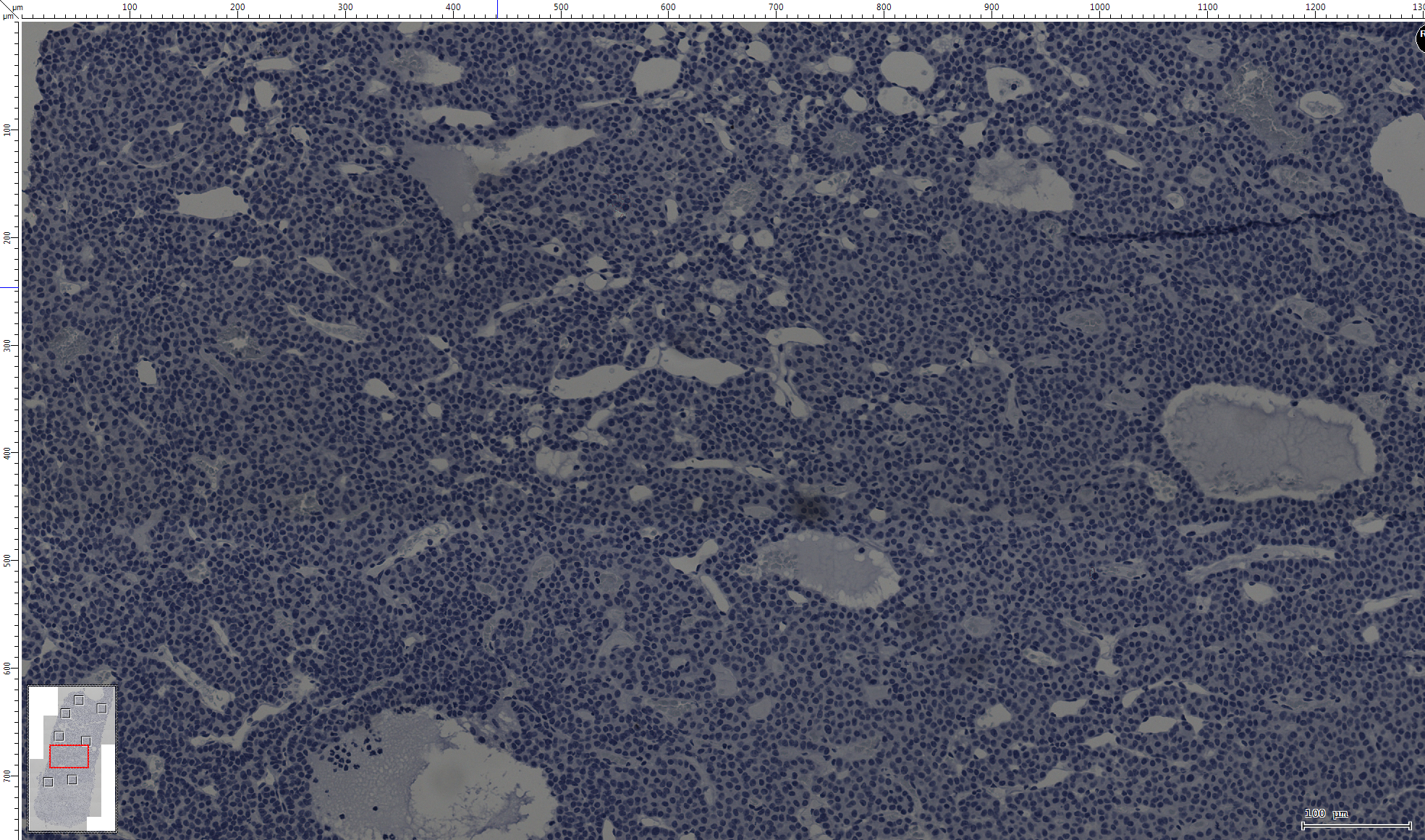

Supplement: Supplementary file 3 — Source Data for Expanded View and Appendix [file EMMM-12-e10941-s010.zip › Fig EV3/PT K1.TIFF]

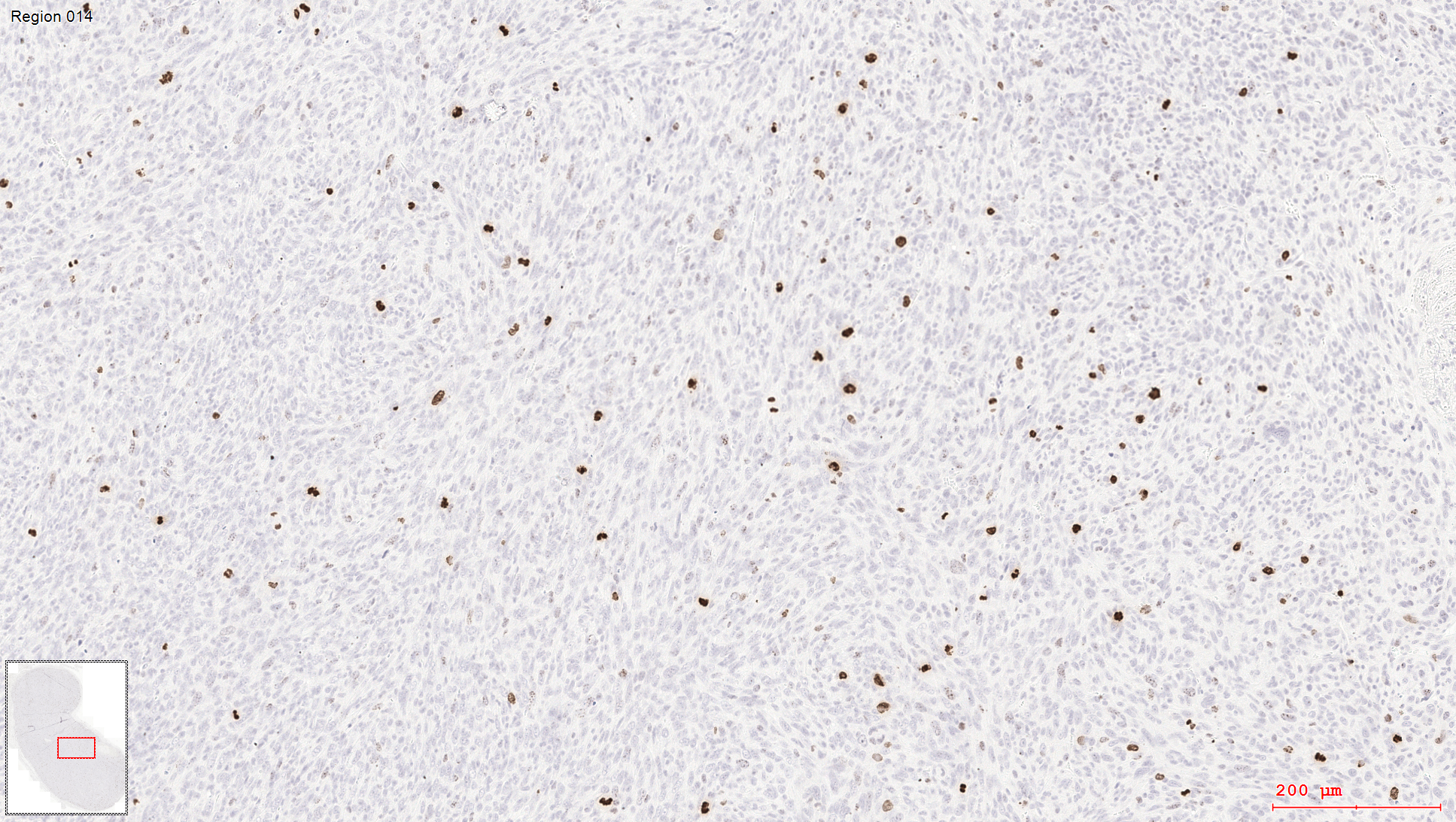

Supplement: Supplementary file 3 — Source Data for Expanded View and Appendix [file EMMM-12-e10941-s010.zip › Fig EV4/No cMet Amp Tepotinib. anti-pH3.TIFF]

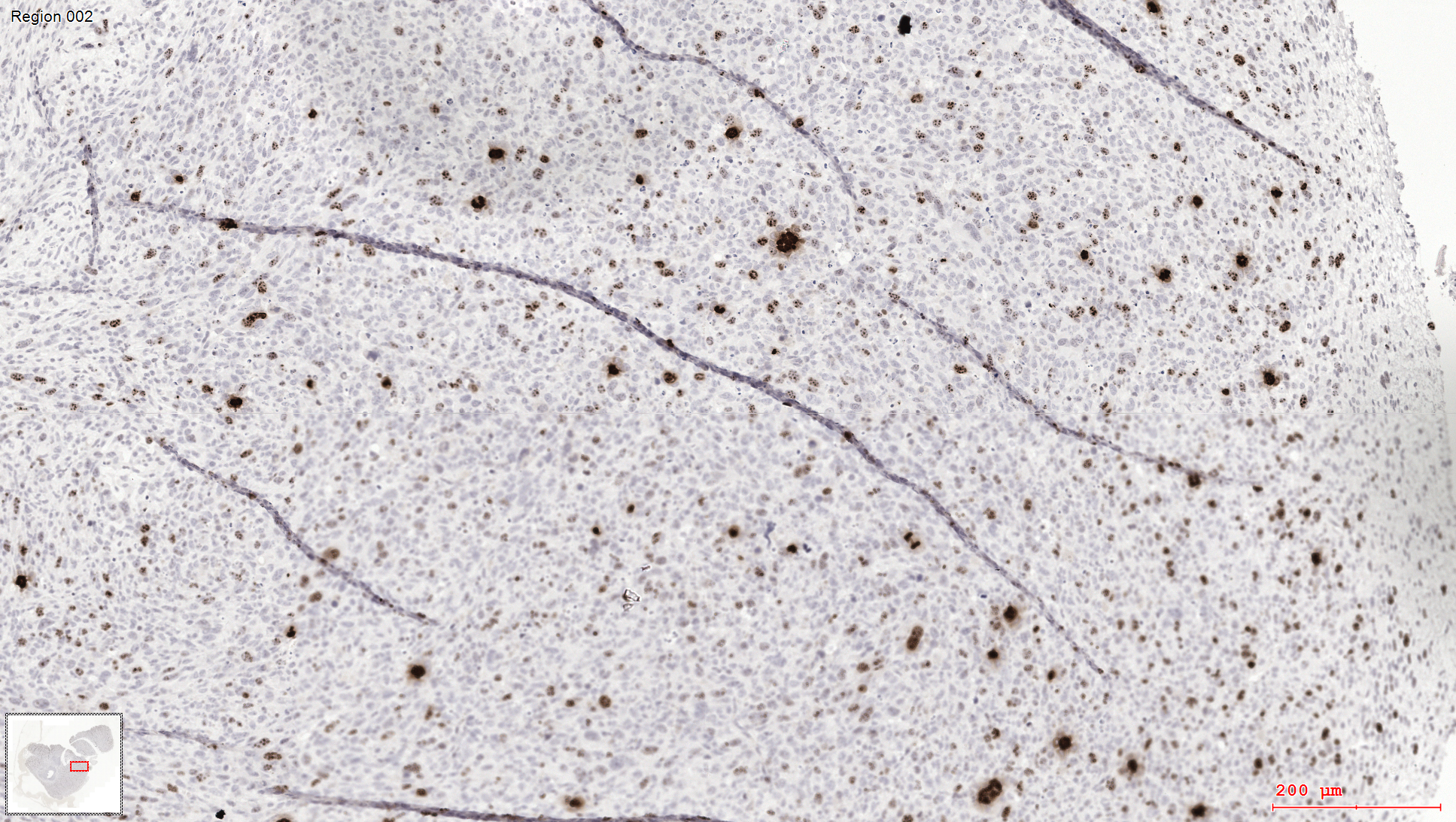

Supplement: Supplementary file 3 — Source Data for Expanded View and Appendix [file EMMM-12-e10941-s010.zip › Fig EV4/cMet Amp. Tepotinib. anti-pH3.TIFF]

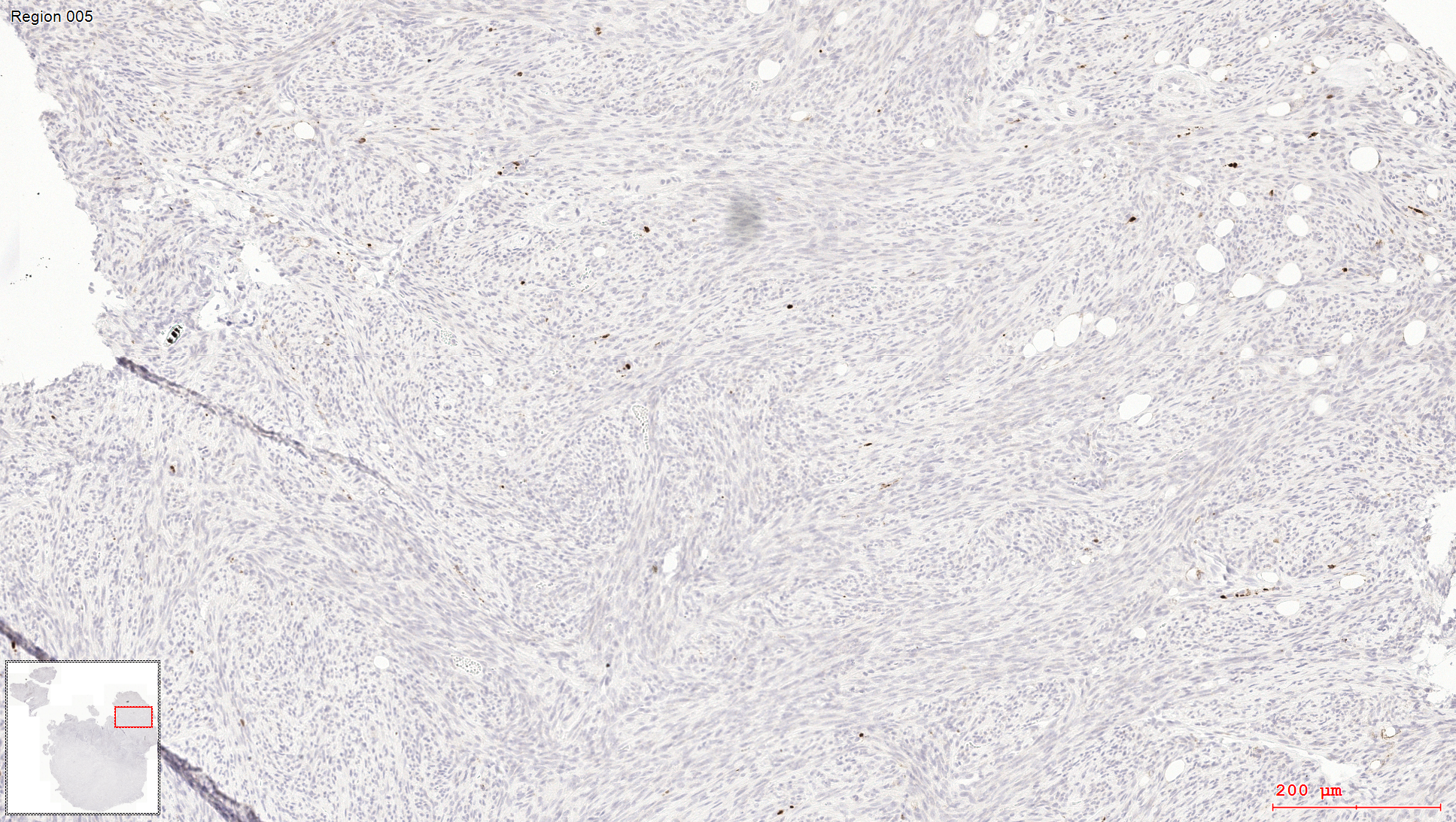

Supplement: Supplementary file 3 — Source Data for Expanded View and Appendix [file EMMM-12-e10941-s010.zip › Fig EV4/No cMet Amp Tepotinib. anti-Caspase3.TIFF]

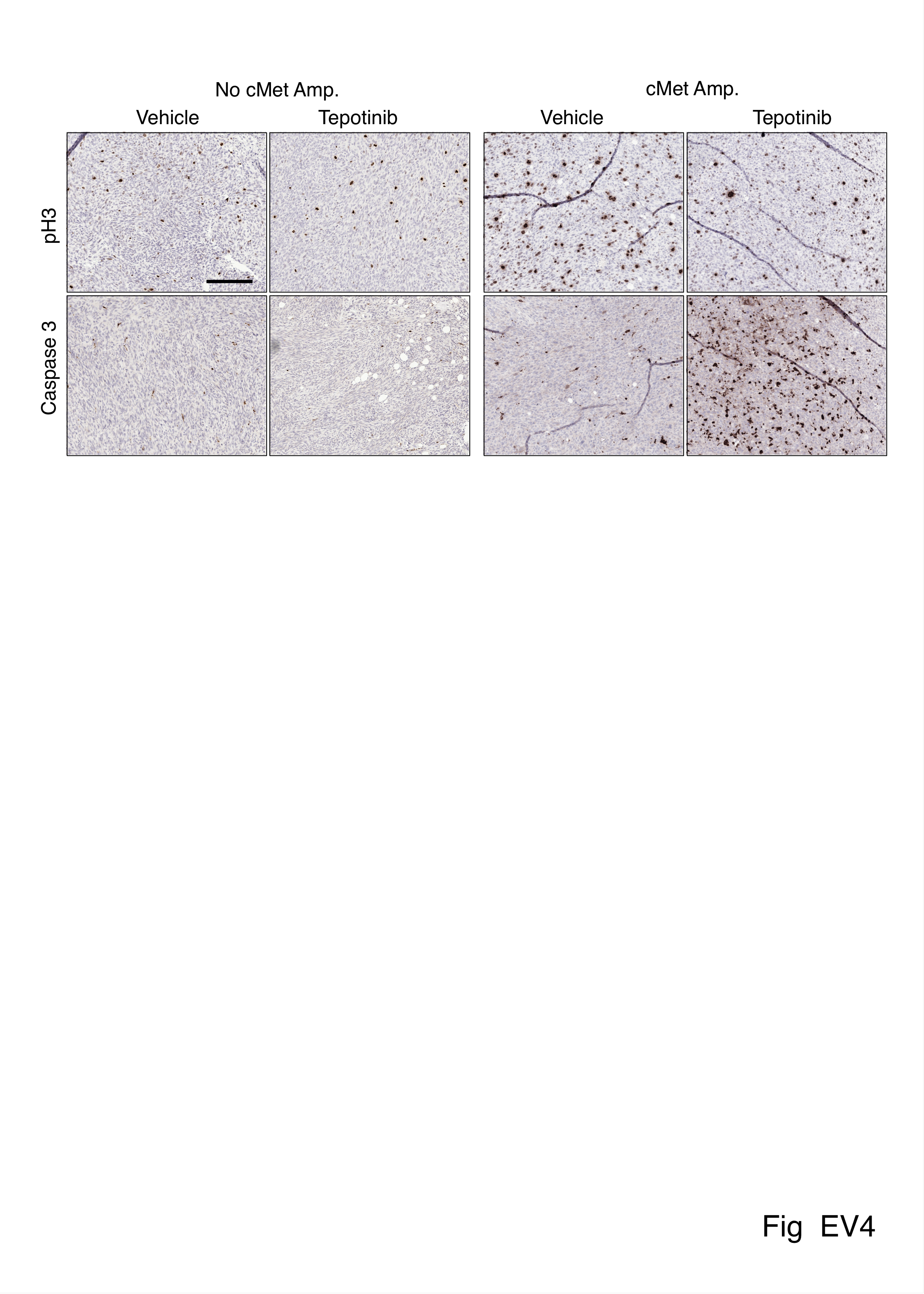

Supplement: Supplementary file 3 — Source Data for Expanded View and Appendix [file EMMM-12-e10941-s010.zip › Fig EV4/Fig EV4.tiff]

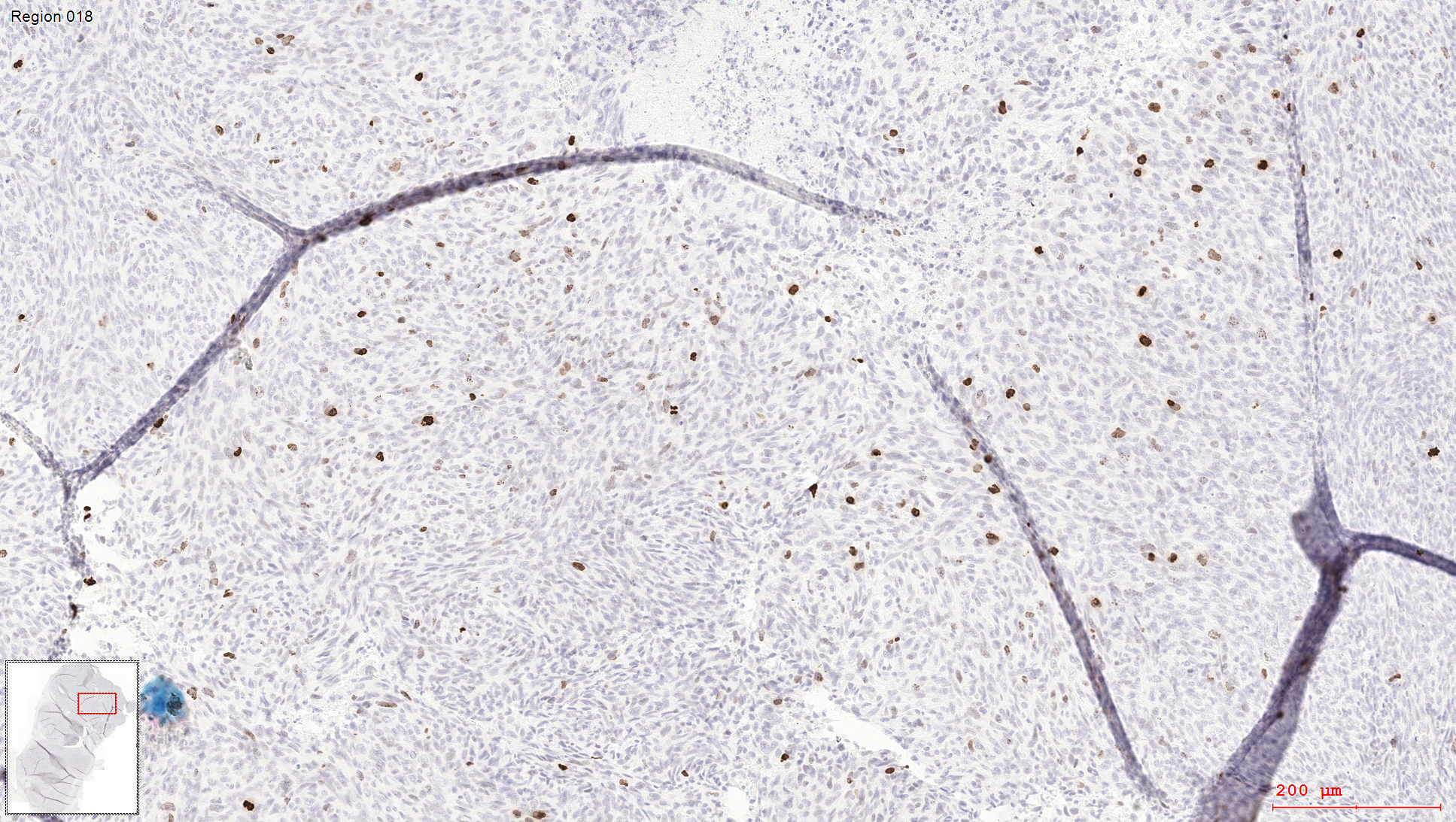

Supplement: Supplementary file 3 — Source Data for Expanded View and Appendix [file EMMM-12-e10941-s010.zip › Fig EV4/No cMet Amp Control. anti-pH3.TIFF]

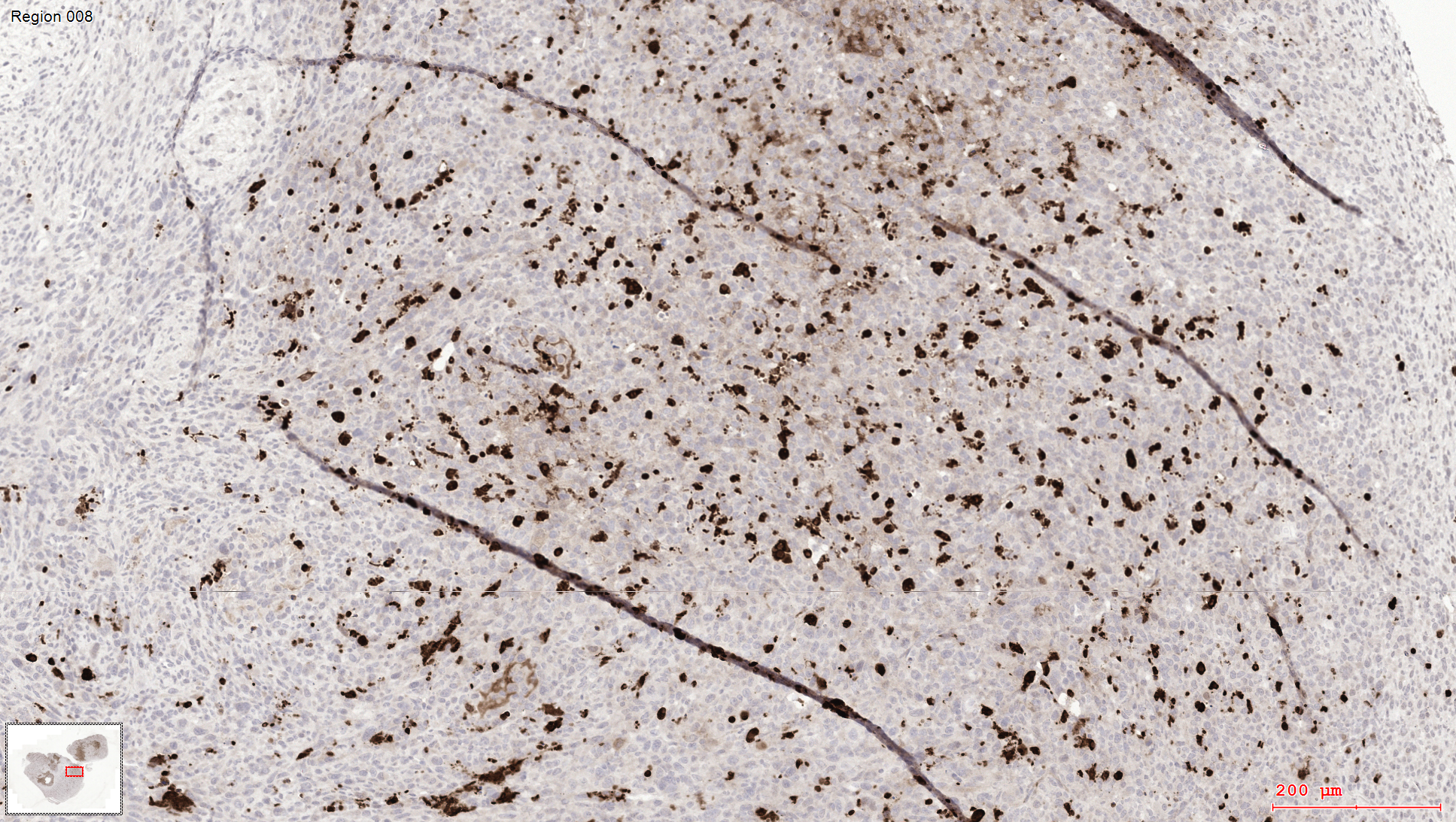

Supplement: Supplementary file 3 — Source Data for Expanded View and Appendix [file EMMM-12-e10941-s010.zip › Fig EV4/cMet Amp. Tepotinib. anti-Caspase3.TIFF]

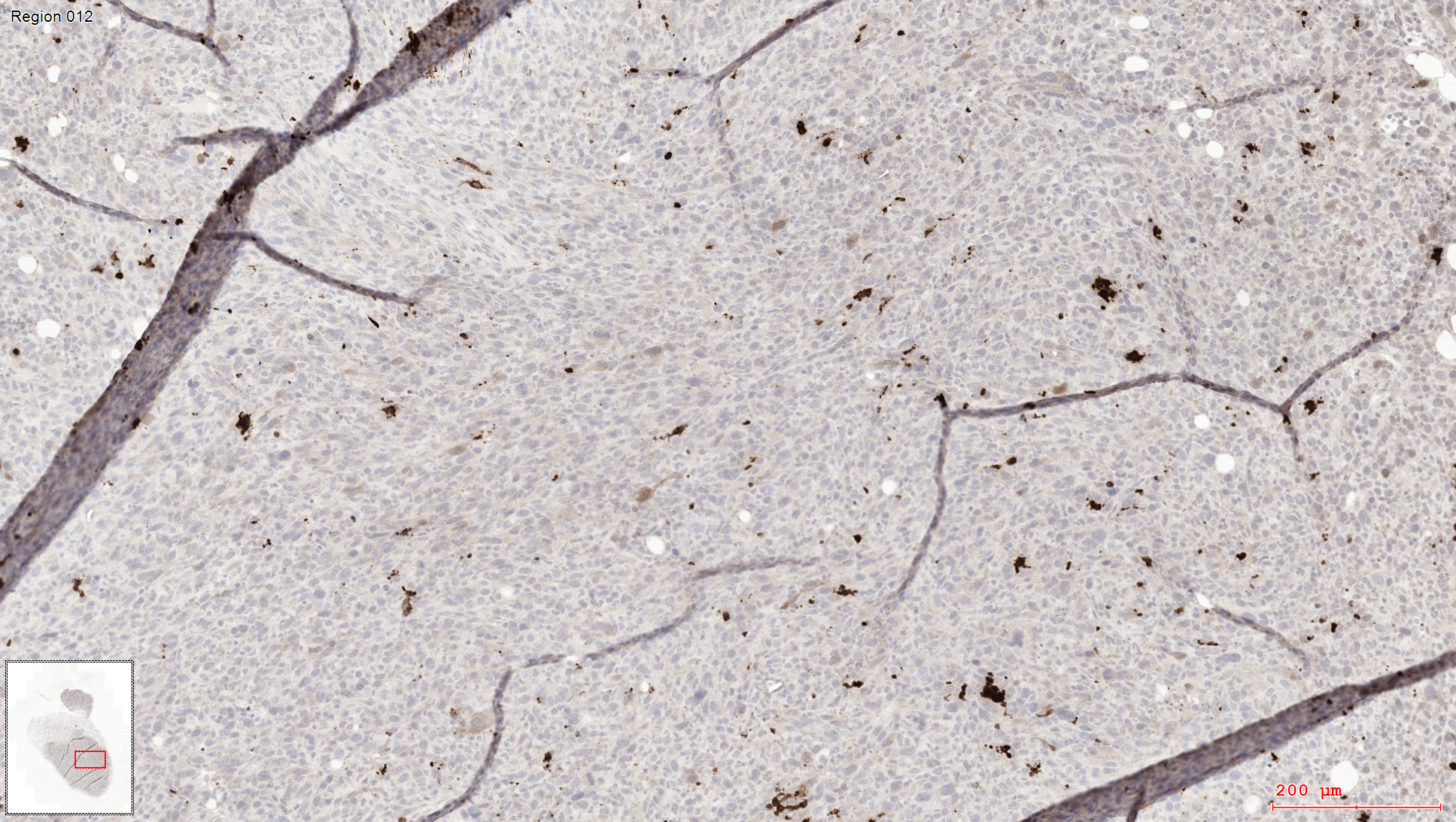

Supplement: Supplementary file 3 — Source Data for Expanded View and Appendix [file EMMM-12-e10941-s010.zip › Fig EV4/cMet Amp. Control. anti-Caspase3.TIFF]

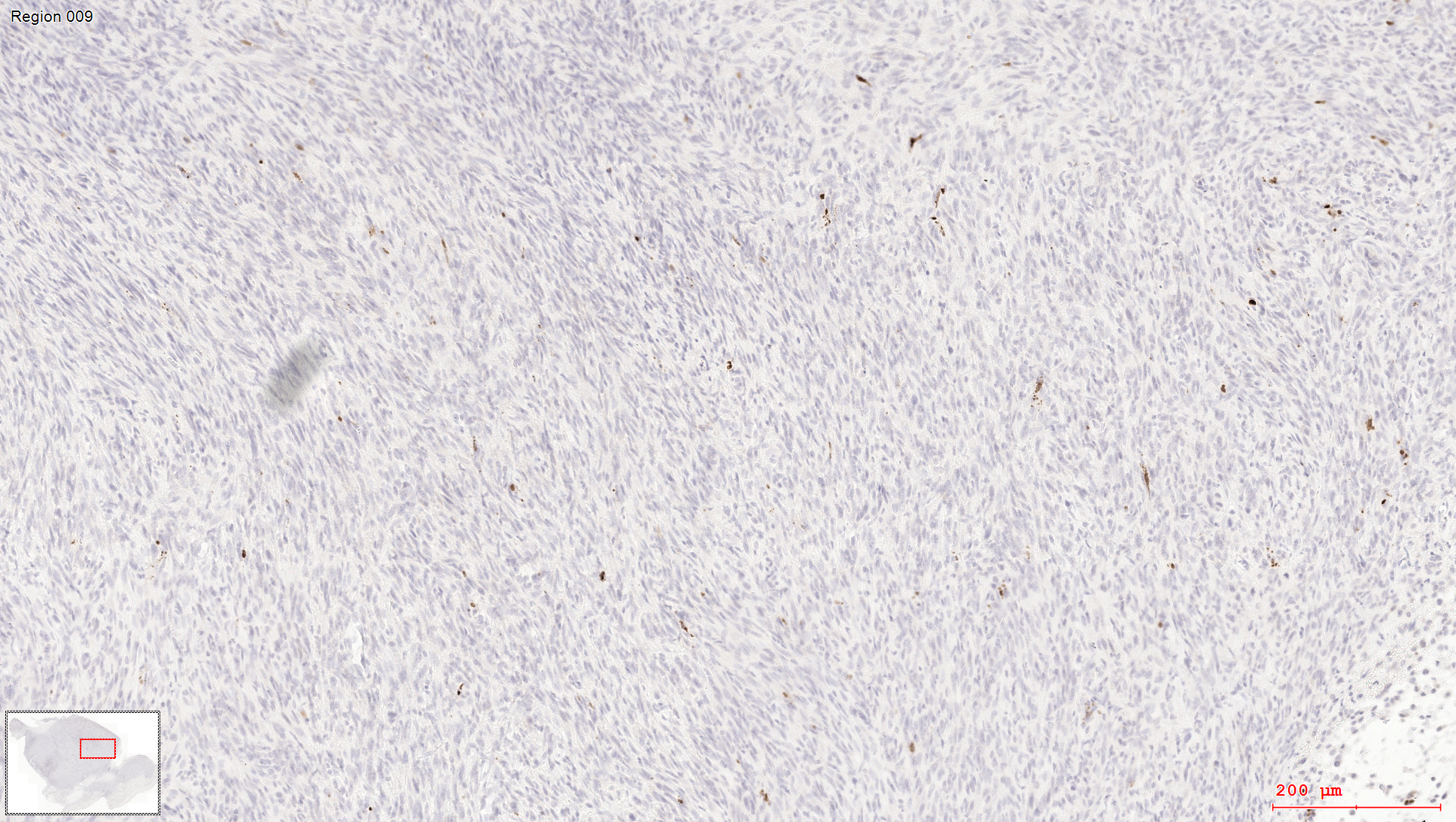

Supplement: Supplementary file 3 — Source Data for Expanded View and Appendix [file EMMM-12-e10941-s010.zip › Fig EV4/No cMet Amp Control. anti-Caspase3.TIFF]

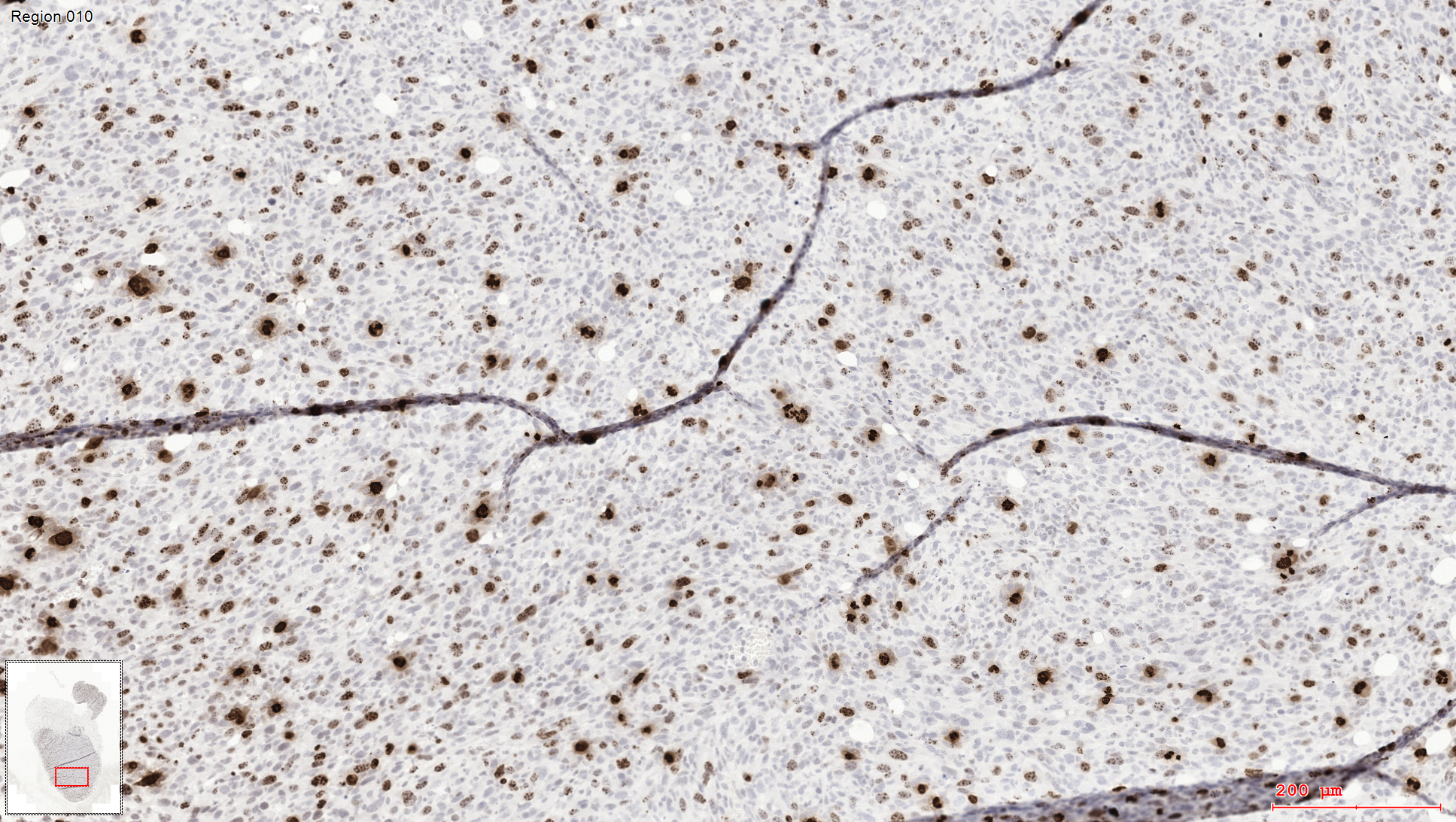

Supplement: Supplementary file 3 — Source Data for Expanded View and Appendix [file EMMM-12-e10941-s010.zip › Fig EV4/cMet Amp. Control. anti-pH3.TIFF]

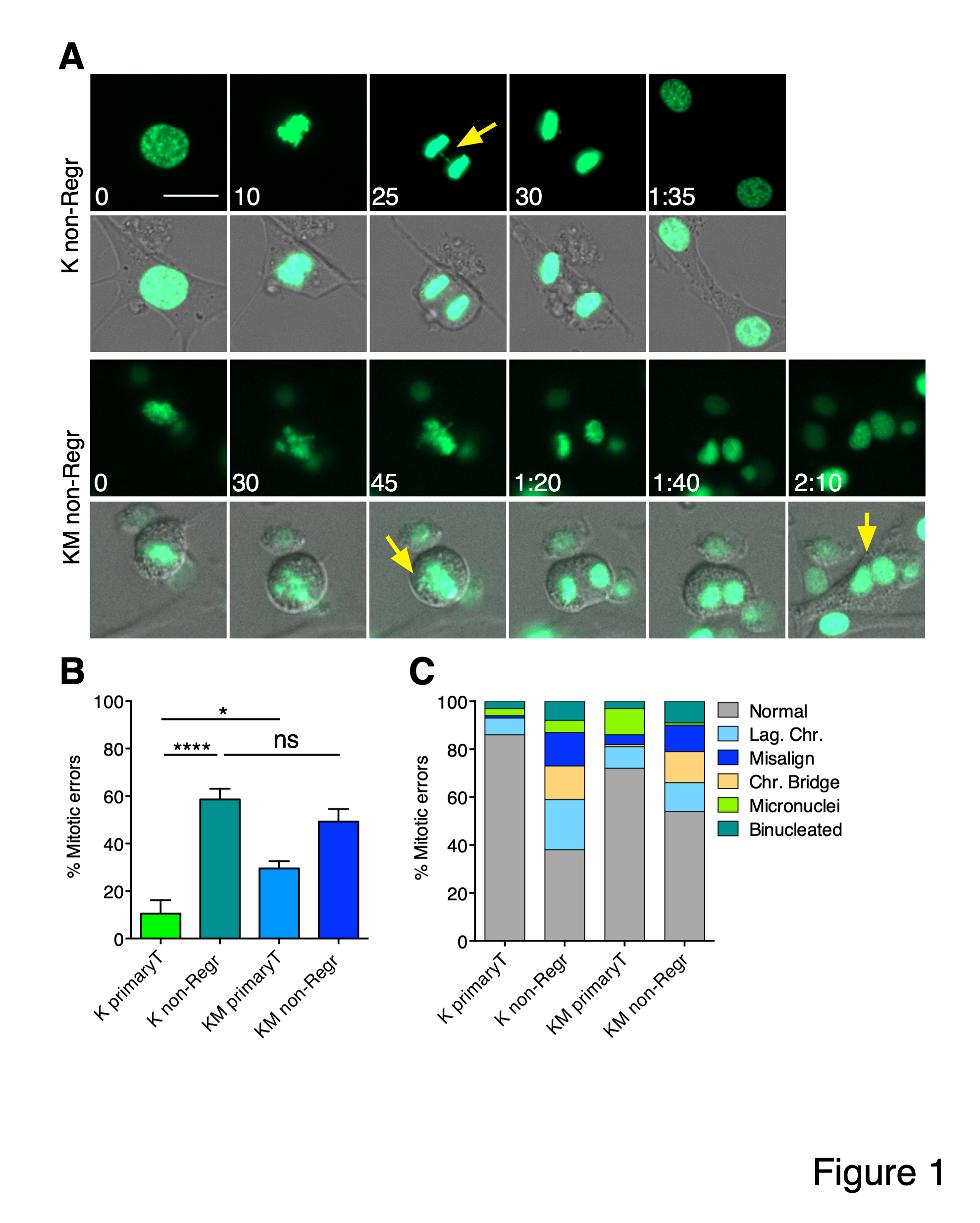

Supplement: Supplementary file 5 — Source Data for Figure 1 [file EMMM-12-e10941-s003.zip › Figure_1/Figure_1.tiff]

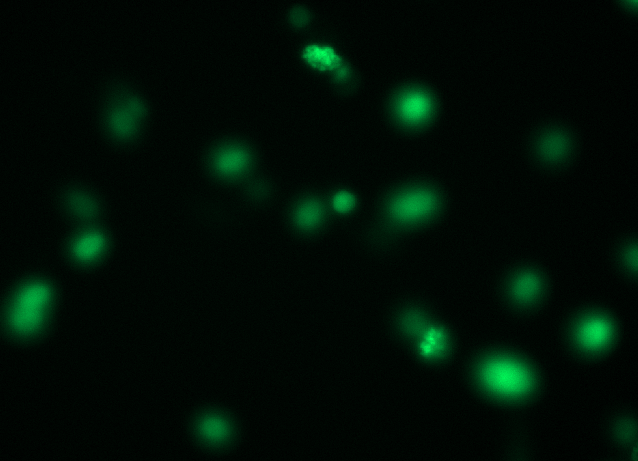

Supplement: Supplementary file 5 — Source Data for Figure 1 [file EMMM-12-e10941-s003.zip › Figure_1/KM_non-regr_T0.tiff]

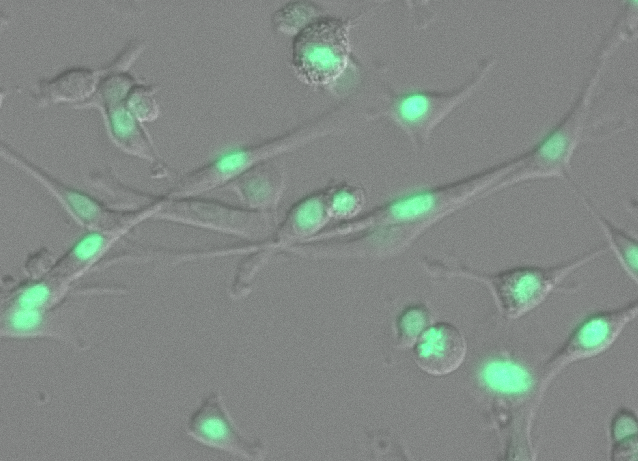

Supplement: Supplementary file 5 — Source Data for Figure 1 [file EMMM-12-e10941-s003.zip › Figure_1/KM_non-regr_T0_BF.tiff]

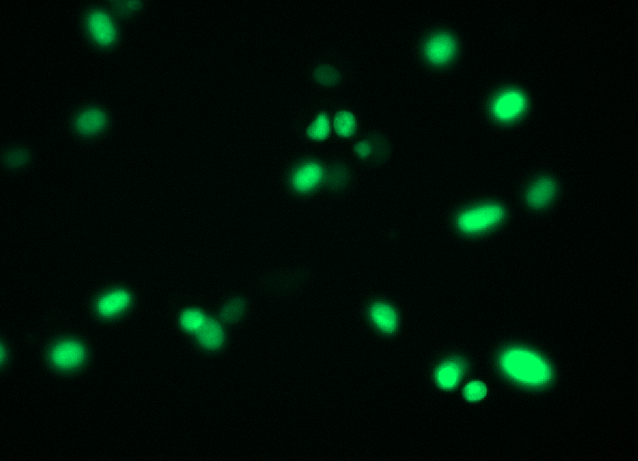

Supplement: Supplementary file 5 — Source Data for Figure 1 [file EMMM-12-e10941-s003.zip › Figure_1/KM_non-regr_T100.tiff]

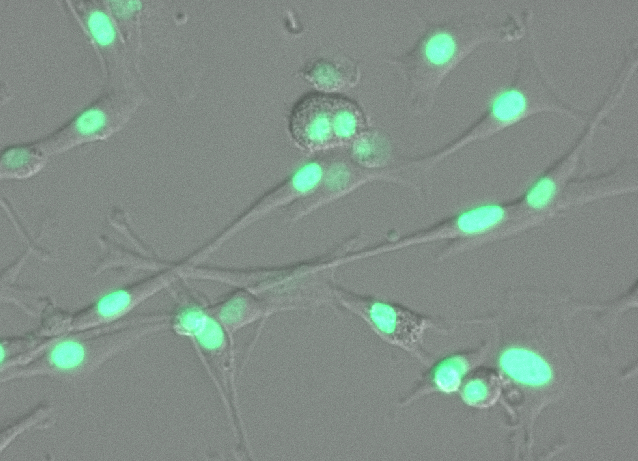

Supplement: Supplementary file 5 — Source Data for Figure 1 [file EMMM-12-e10941-s003.zip › Figure_1/KM_non-regr_T100_bf.tiff]

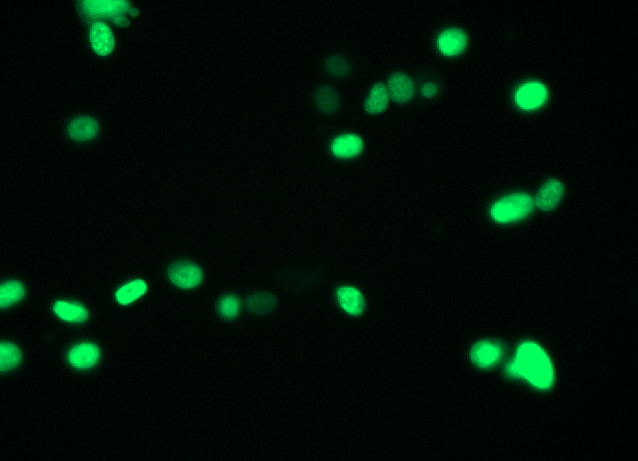

Supplement: Supplementary file 5 — Source Data for Figure 1 [file EMMM-12-e10941-s003.zip › Figure_1/KM_non-regr_T130.tiff]

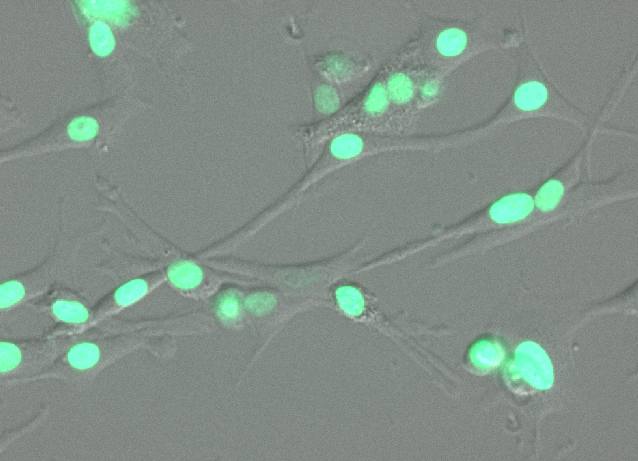

Supplement: Supplementary file 5 — Source Data for Figure 1 [file EMMM-12-e10941-s003.zip › Figure_1/KM_non-regr_T130_BF.tiff]

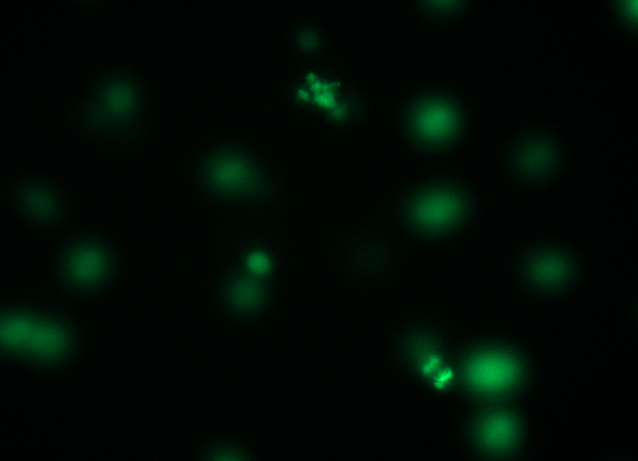

Supplement: Supplementary file 5 — Source Data for Figure 1 [file EMMM-12-e10941-s003.zip › Figure_1/KM_non-regr_T30.tiff]

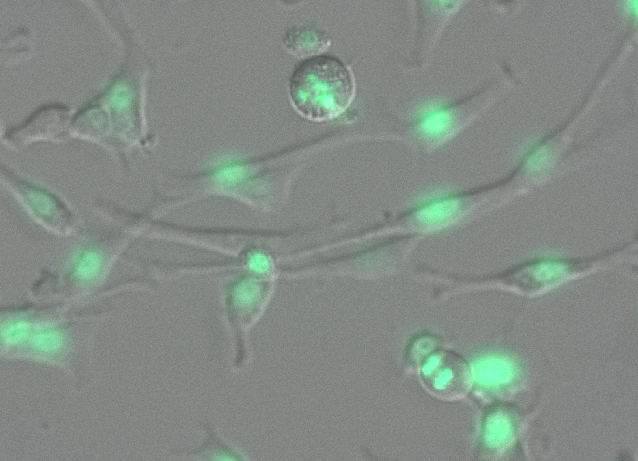

Supplement: Supplementary file 5 — Source Data for Figure 1 [file EMMM-12-e10941-s003.zip › Figure_1/KM_non-regr_T30_BF.tiff]

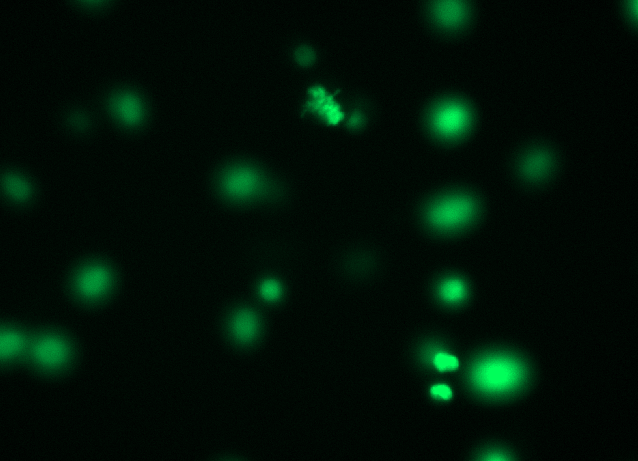

Supplement: Supplementary file 5 — Source Data for Figure 1 [file EMMM-12-e10941-s003.zip › Figure_1/KM_non-regr_T45.tiff]

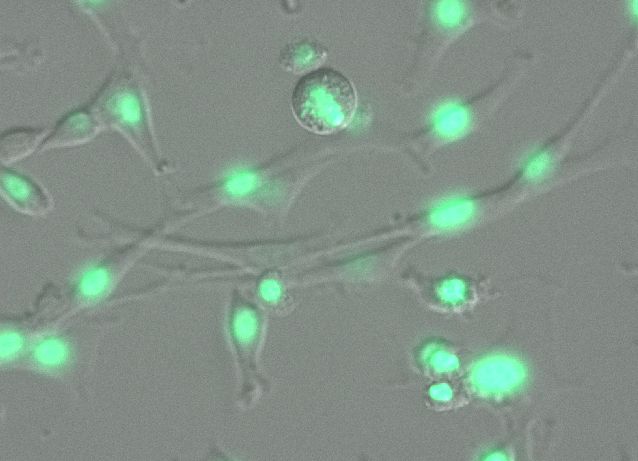

Supplement: Supplementary file 5 — Source Data for Figure 1 [file EMMM-12-e10941-s003.zip › Figure_1/KM_non-regr_T45_BF.tiff]

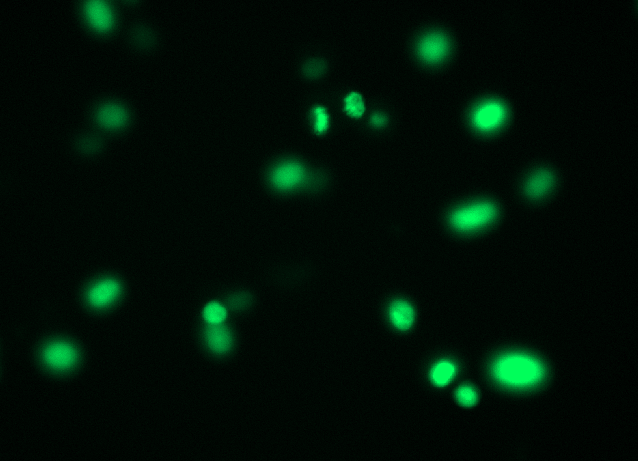

Supplement: Supplementary file 5 — Source Data for Figure 1 [file EMMM-12-e10941-s003.zip › Figure_1/KM_non-regr_T80.tiff]

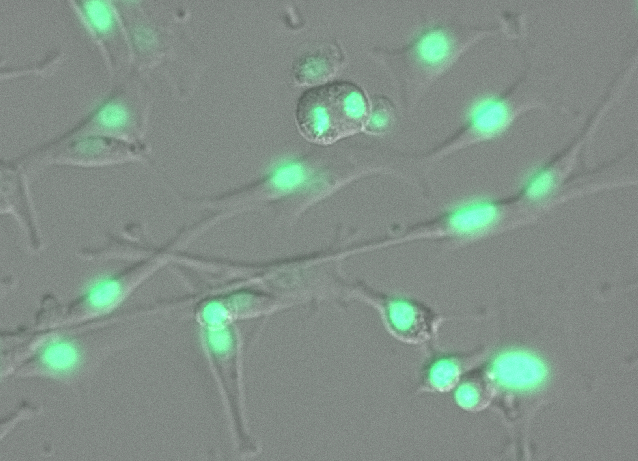

Supplement: Supplementary file 5 — Source Data for Figure 1 [file EMMM-12-e10941-s003.zip › Figure_1/KM_non-regr_T80_BF.tiff]

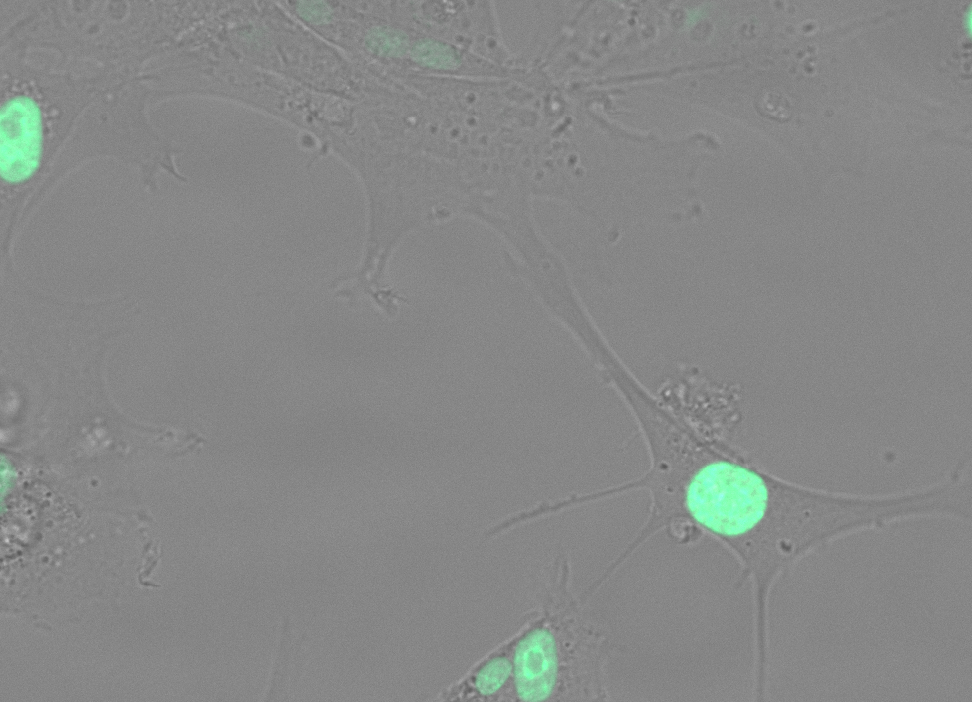

Supplement: Supplementary file 5 — Source Data for Figure 1 [file EMMM-12-e10941-s003.zip › Figure_1/K_non-Regr.T0_BF.tiff]

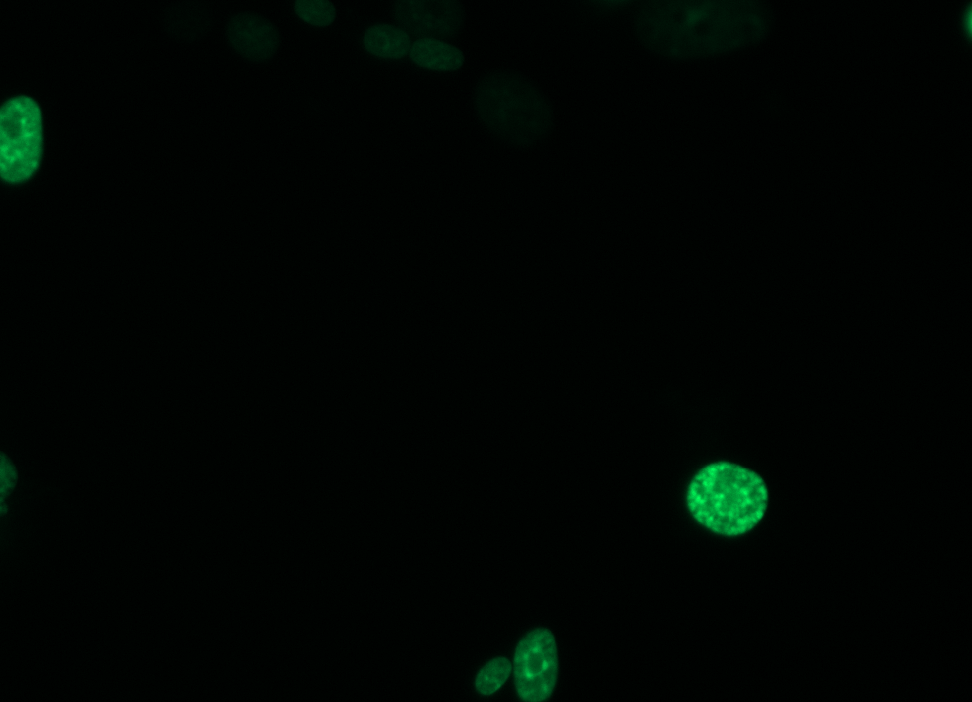

Supplement: Supplementary file 5 — Source Data for Figure 1 [file EMMM-12-e10941-s003.zip › Figure_1/K_non-Regr_T0.tiff]

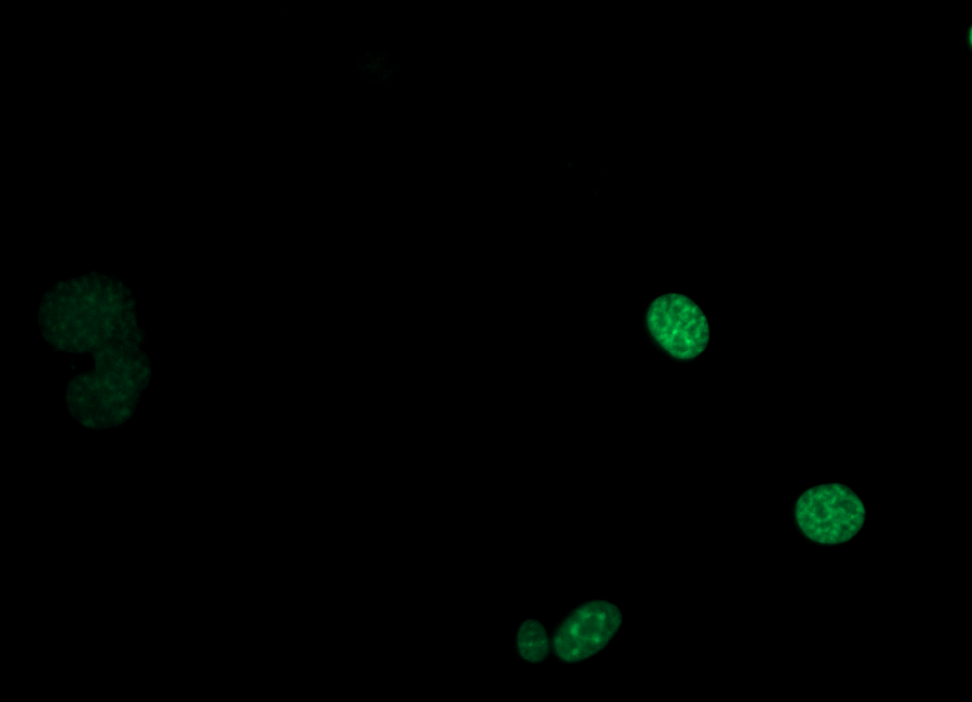

Supplement: Supplementary file 5 — Source Data for Figure 1 [file EMMM-12-e10941-s003.zip › Figure_1/K_non-Regr_T1.35_.tiff]

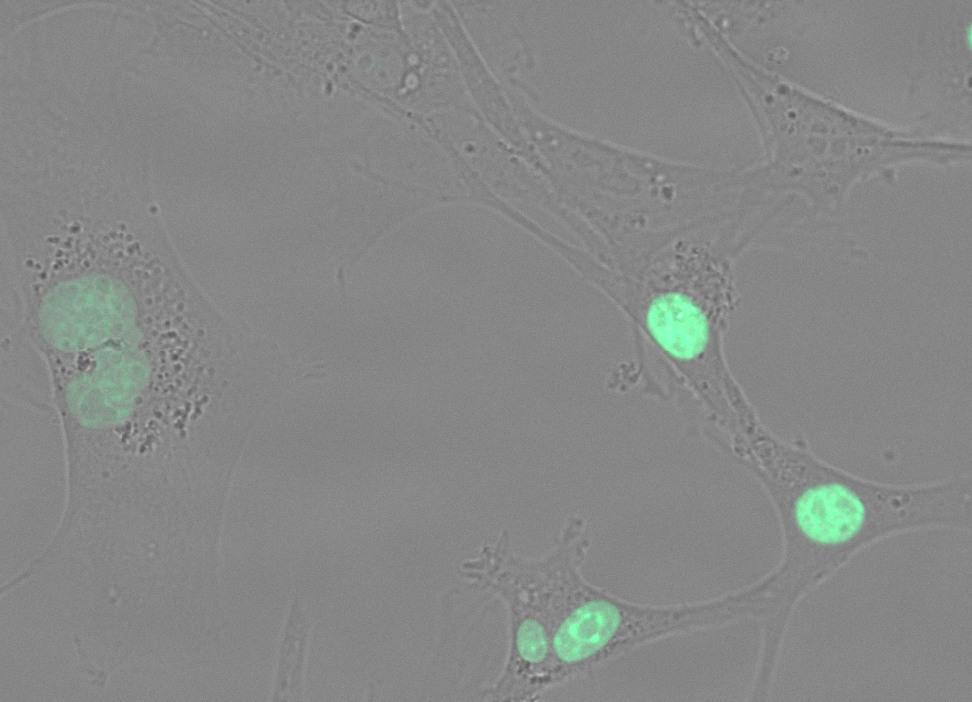

Supplement: Supplementary file 5 — Source Data for Figure 1 [file EMMM-12-e10941-s003.zip › Figure_1/K_non-Regr_T1.35__BF.tiff]

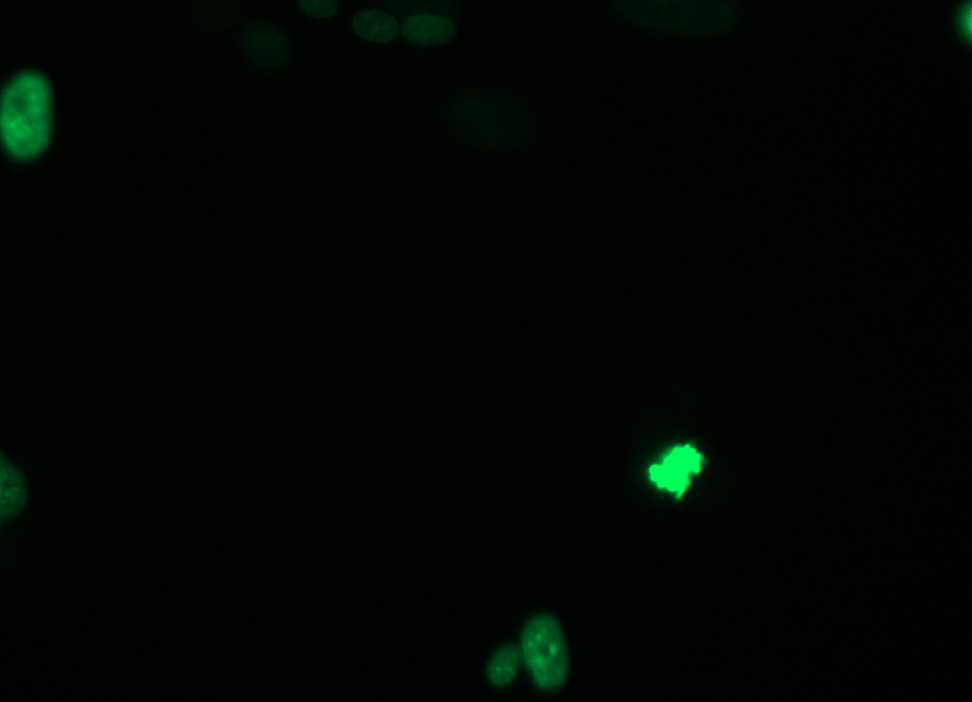

Supplement: Supplementary file 5 — Source Data for Figure 1 [file EMMM-12-e10941-s003.zip › Figure_1/K_non-Regr_T10.tiff]

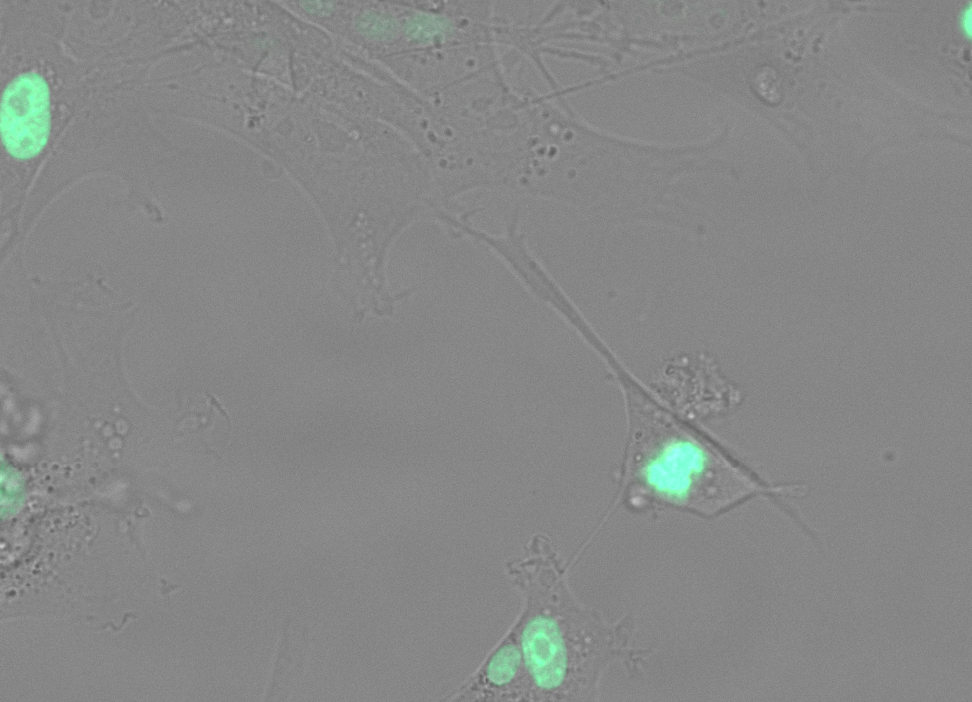

Supplement: Supplementary file 5 — Source Data for Figure 1 [file EMMM-12-e10941-s003.zip › Figure_1/K_non-Regr_T10_BF.tiff]

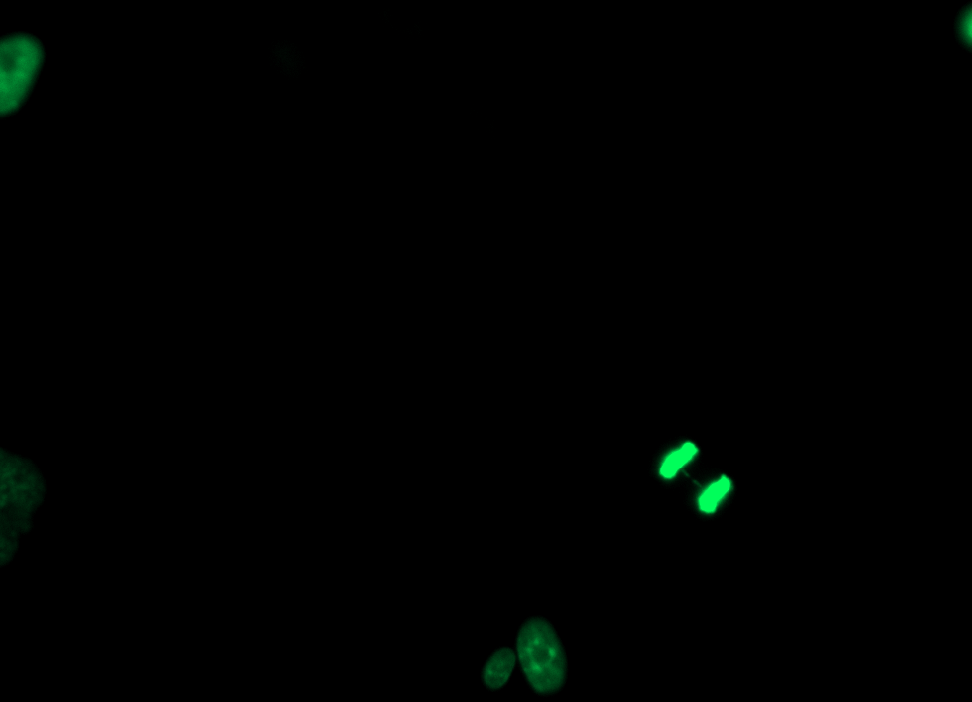

Supplement: Supplementary file 5 — Source Data for Figure 1 [file EMMM-12-e10941-s003.zip › Figure_1/K_non-Regr_T25.tiff]

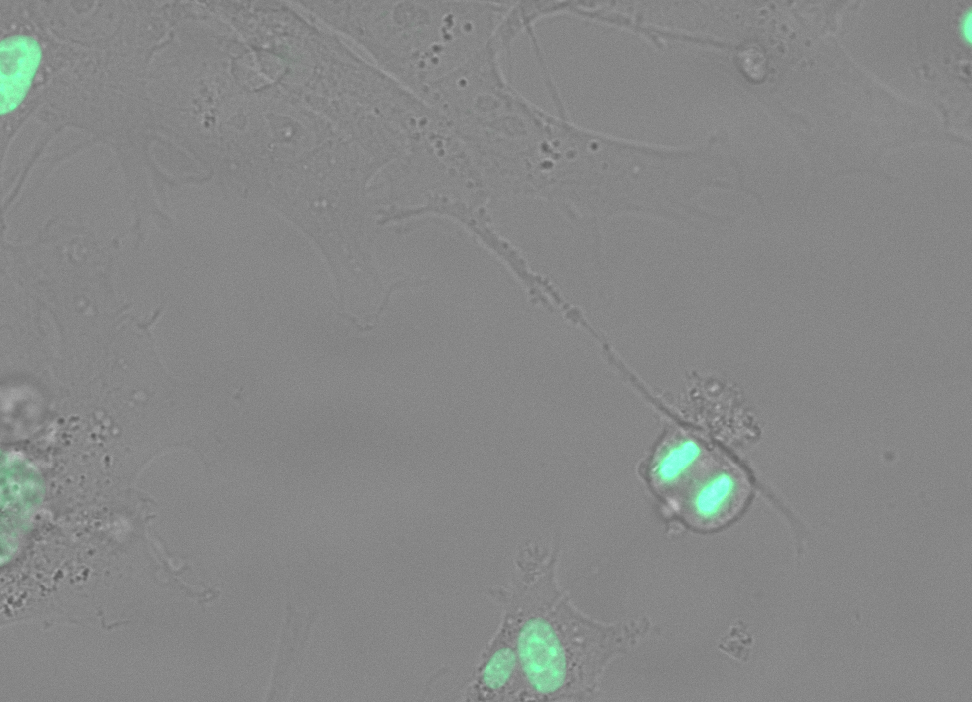

Supplement: Supplementary file 5 — Source Data for Figure 1 [file EMMM-12-e10941-s003.zip › Figure_1/K_non-Regr_T25_BF.tiff]

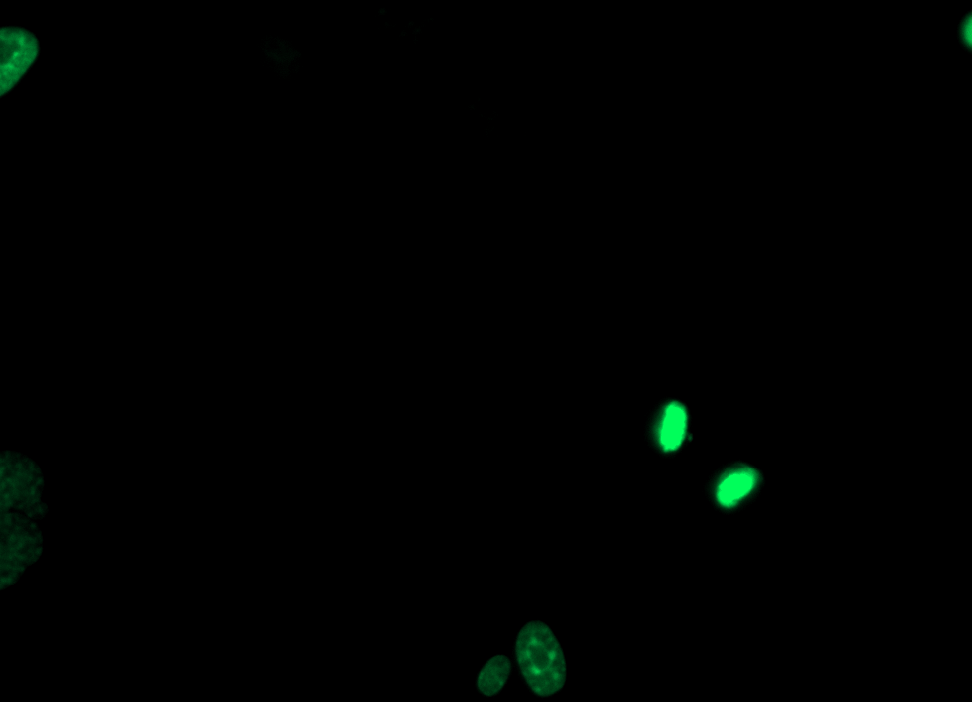

Supplement: Supplementary file 5 — Source Data for Figure 1 [file EMMM-12-e10941-s003.zip › Figure_1/K_non-Regr_T30_.tiff]

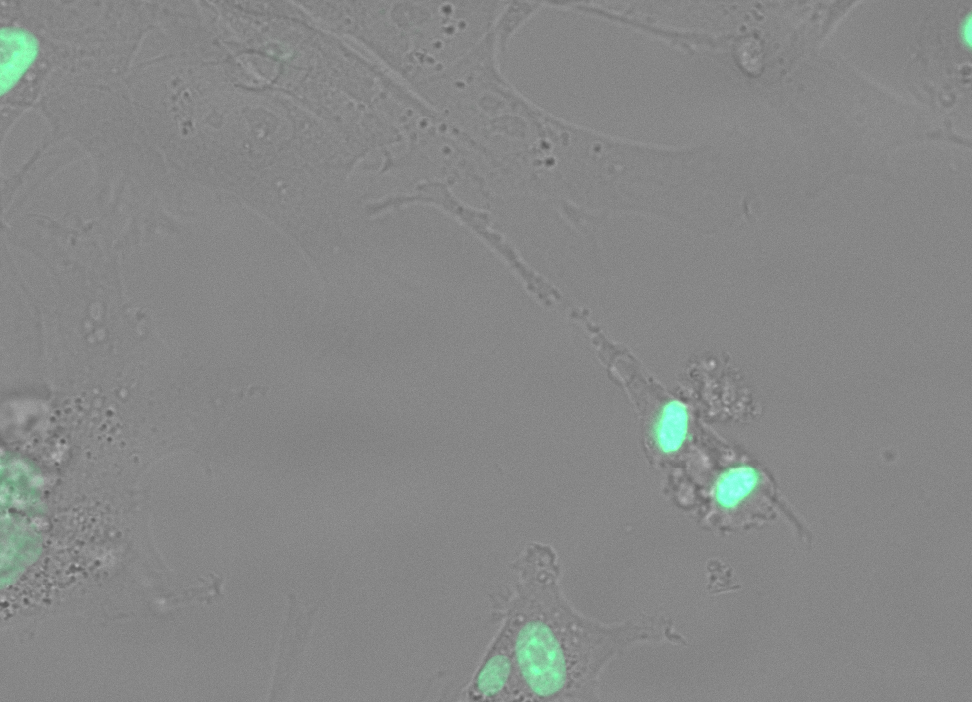

Supplement: Supplementary file 5 — Source Data for Figure 1 [file EMMM-12-e10941-s003.zip › Figure_1/K_non-Regr_T30_BF.tiff]

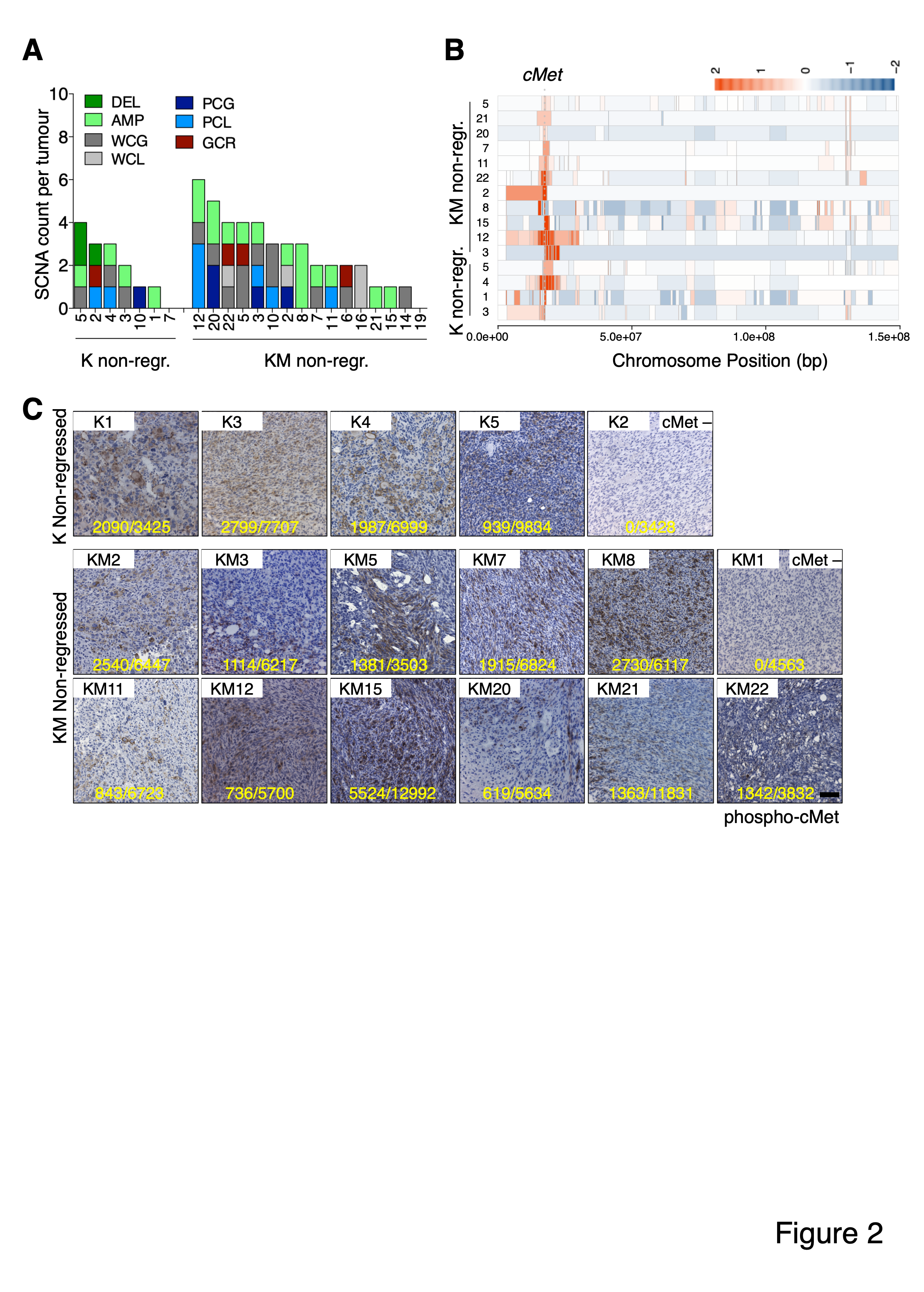

Supplement: Supplementary file 6 — Source Data for Figure 2 [file EMMM-12-e10941-s004.zip › Figure_2/Figure_2.tiff]

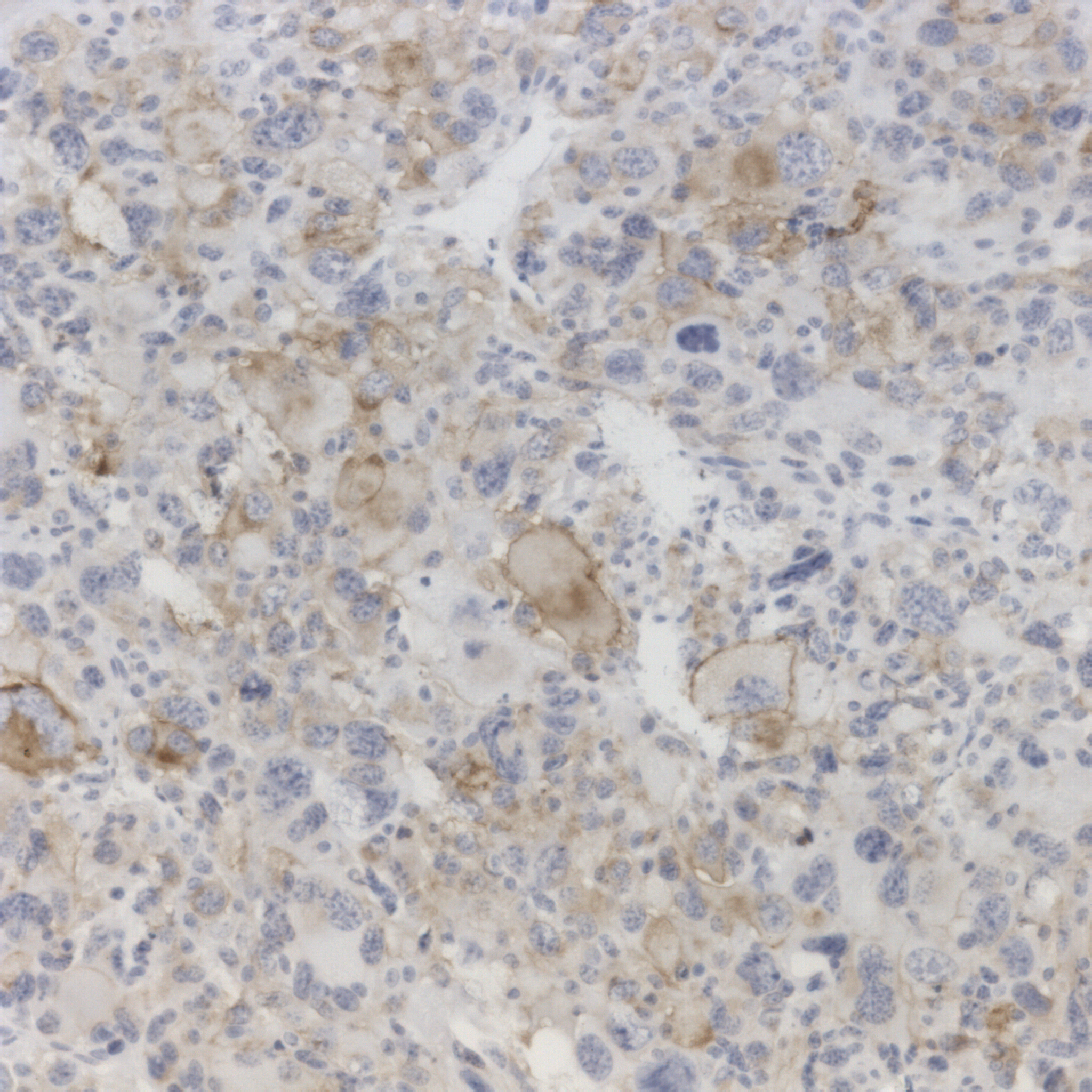

Supplement: Supplementary file 6 — Source Data for Figure 2 [file EMMM-12-e10941-s004.zip › Figure_2/K1.tif]

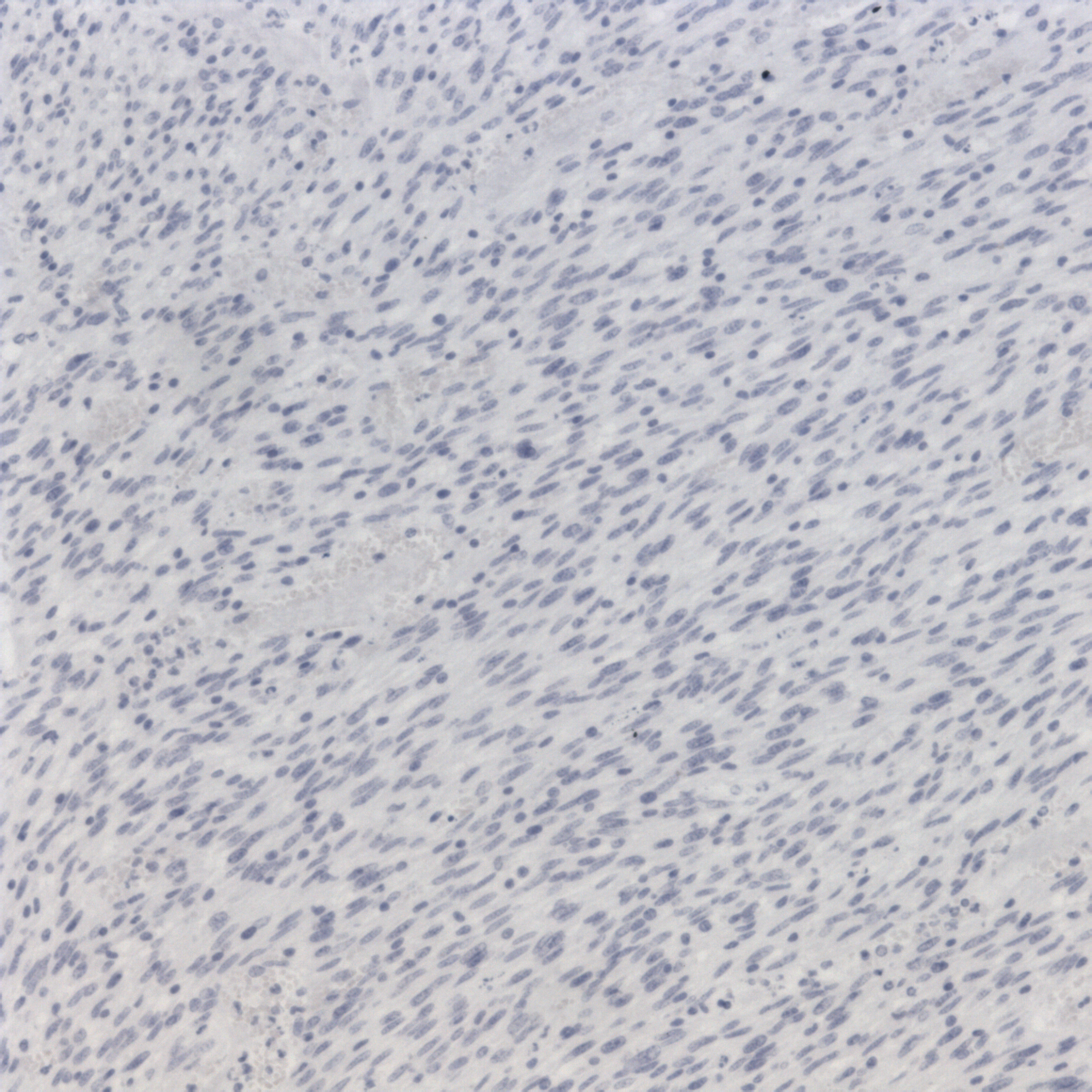

Supplement: Supplementary file 6 — Source Data for Figure 2 [file EMMM-12-e10941-s004.zip › Figure_2/K2.tif]

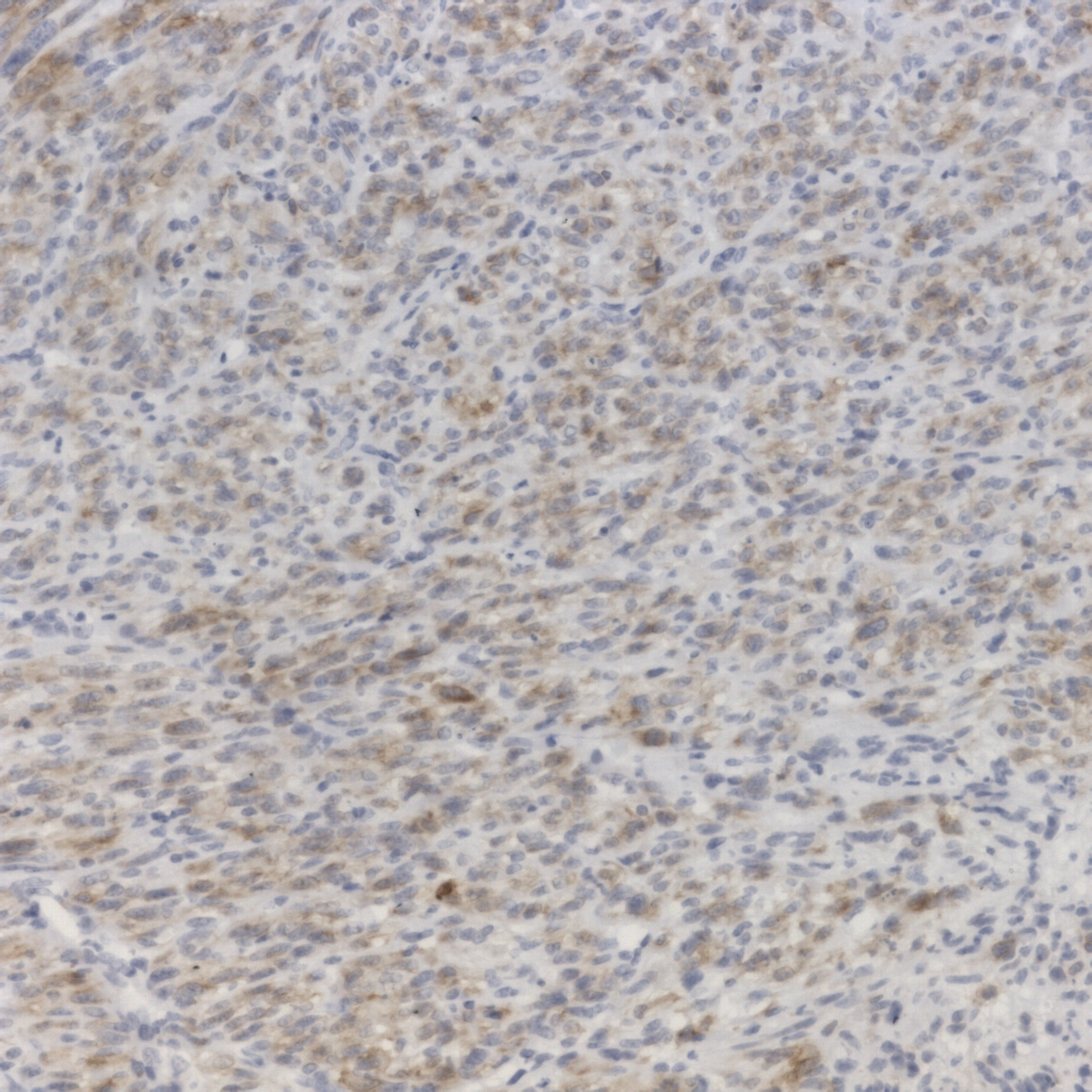

Supplement: Supplementary file 6 — Source Data for Figure 2 [file EMMM-12-e10941-s004.zip › Figure_2/K3.tif]

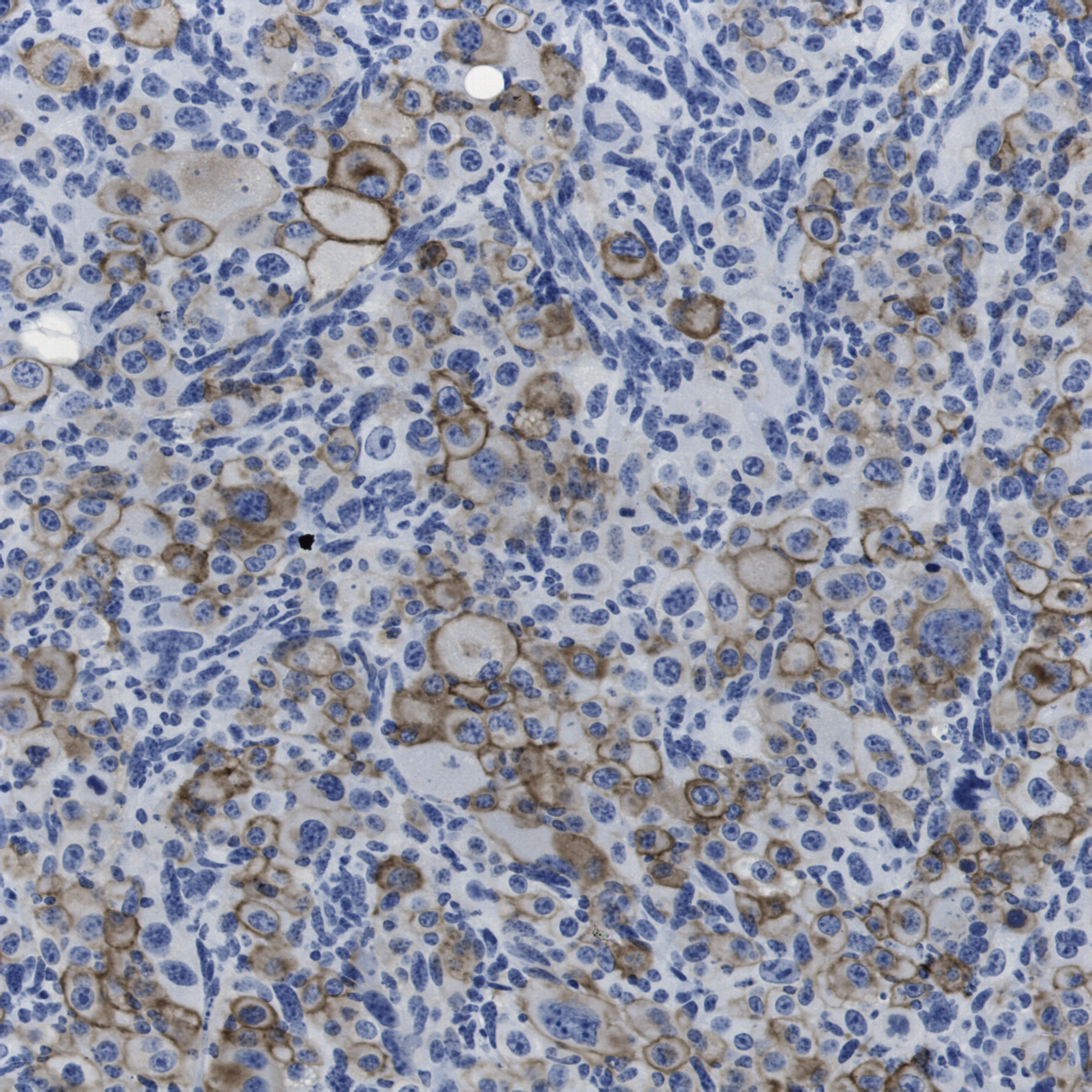

Supplement: Supplementary file 6 — Source Data for Figure 2 [file EMMM-12-e10941-s004.zip › Figure_2/K4.tif]

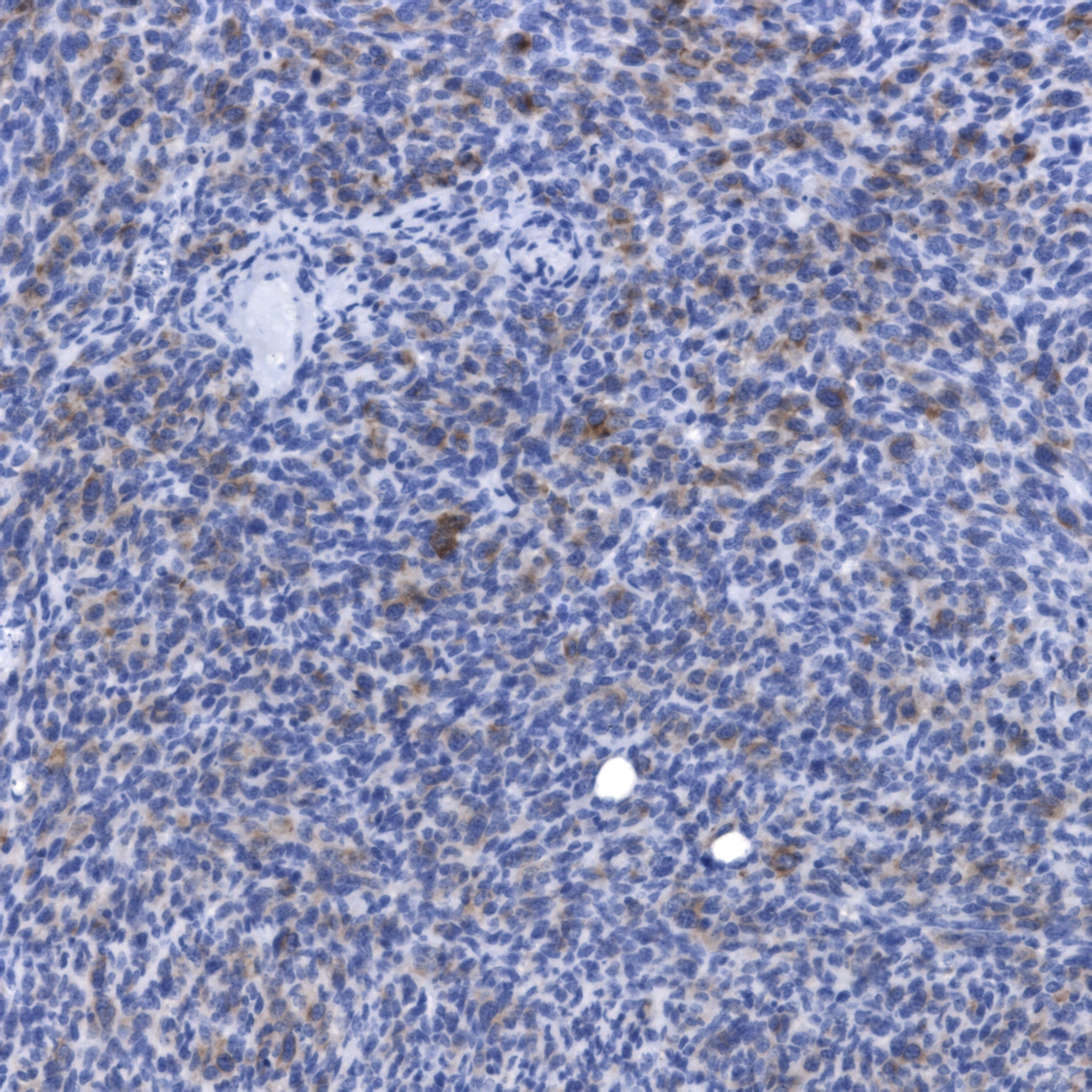

Supplement: Supplementary file 6 — Source Data for Figure 2 [file EMMM-12-e10941-s004.zip › Figure_2/K5.jpg]

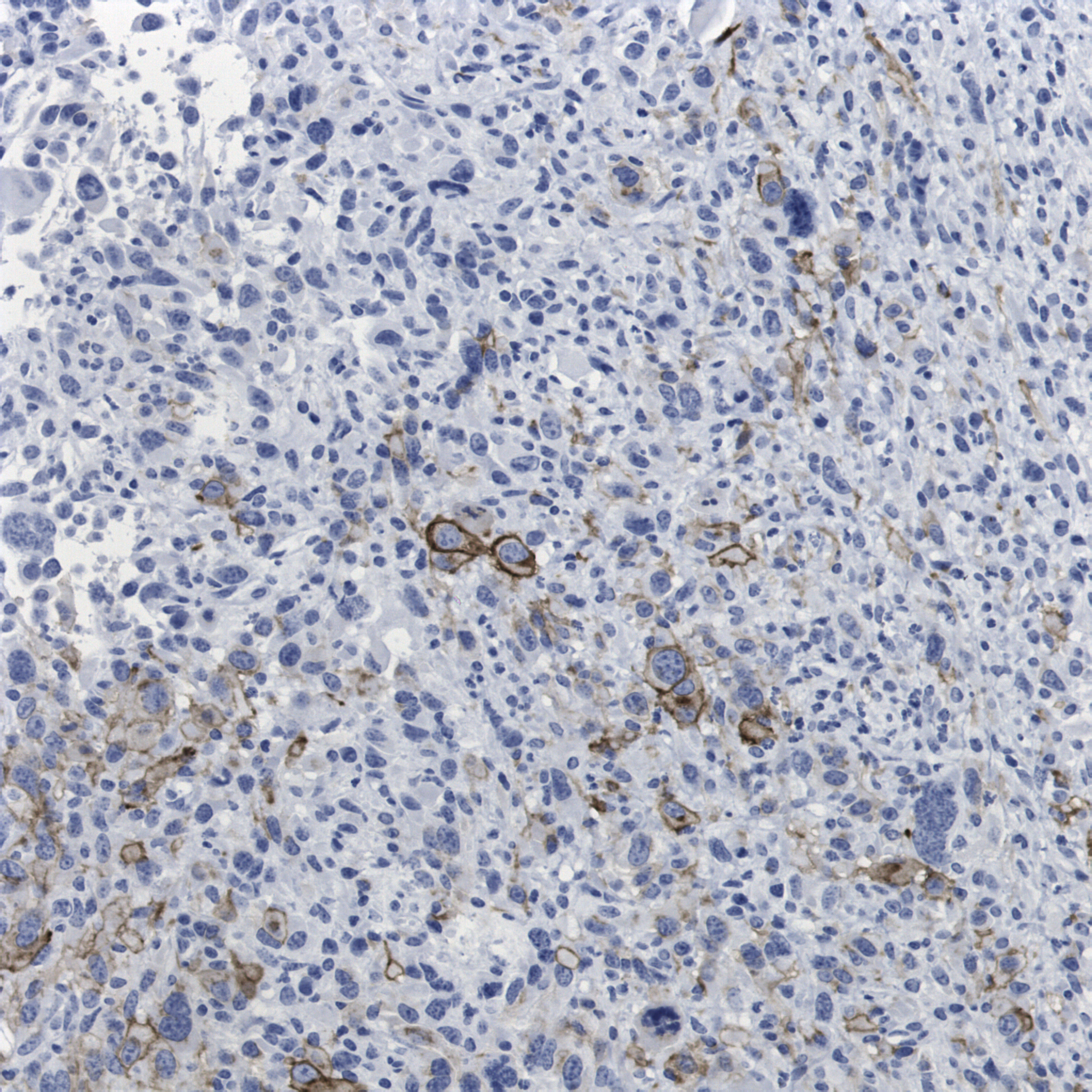

Supplement: Supplementary file 6 — Source Data for Figure 2 [file EMMM-12-e10941-s004.zip › Figure_2/KM11.tif]

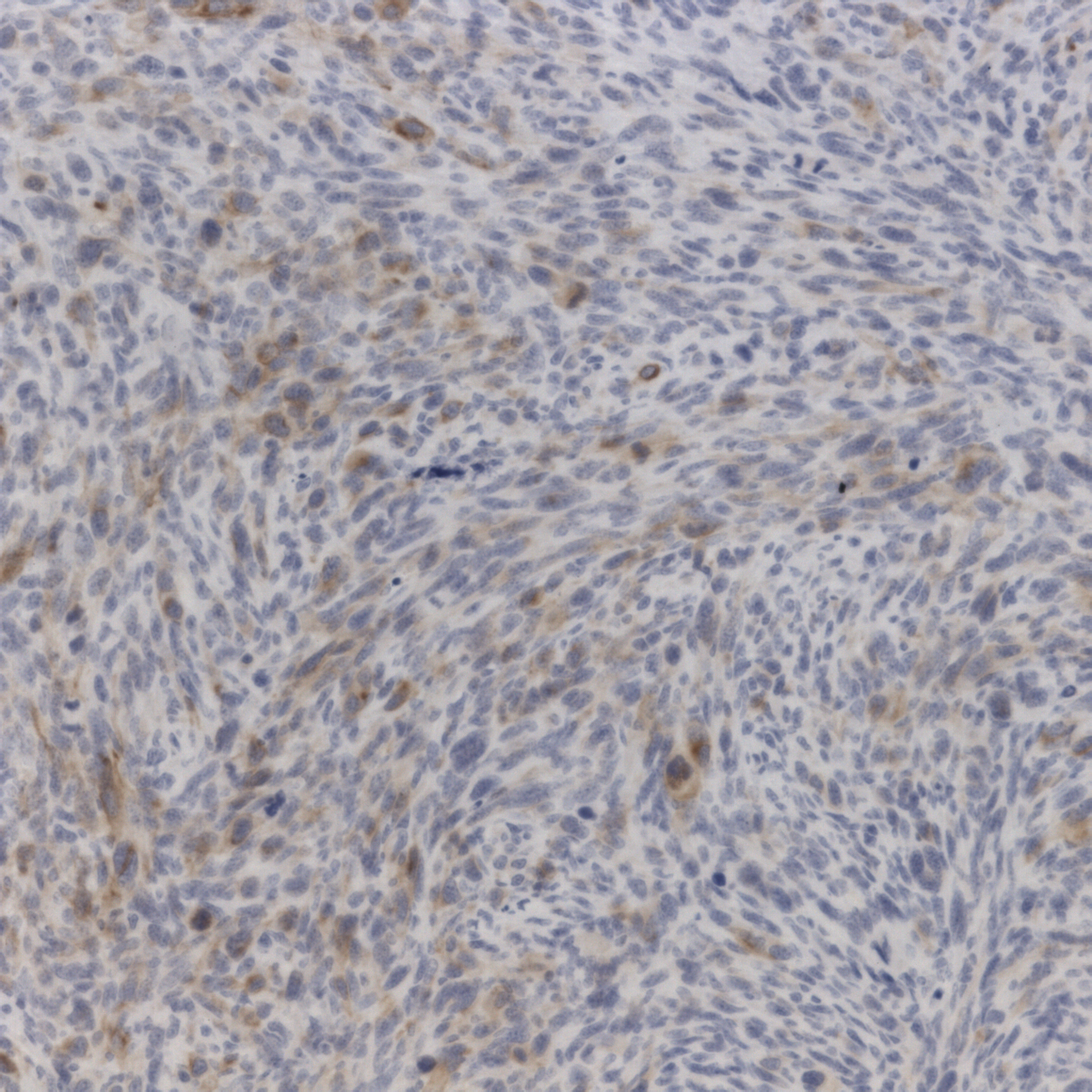

Supplement: Supplementary file 6 — Source Data for Figure 2 [file EMMM-12-e10941-s004.zip › Figure_2/KM12.tif]

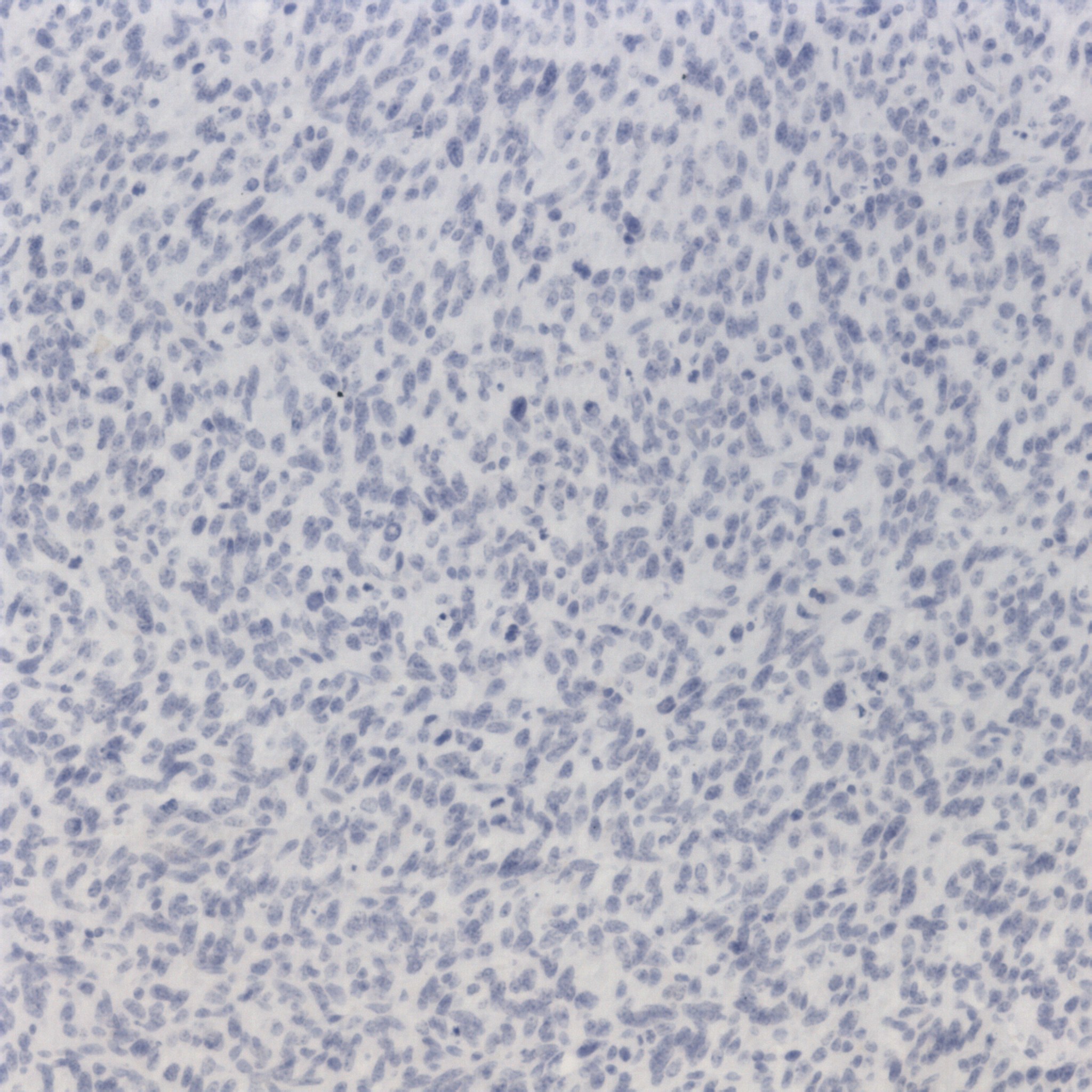

Supplement: Supplementary file 6 — Source Data for Figure 2 [file EMMM-12-e10941-s004.zip › Figure_2/KM1_cMet_-.tif]

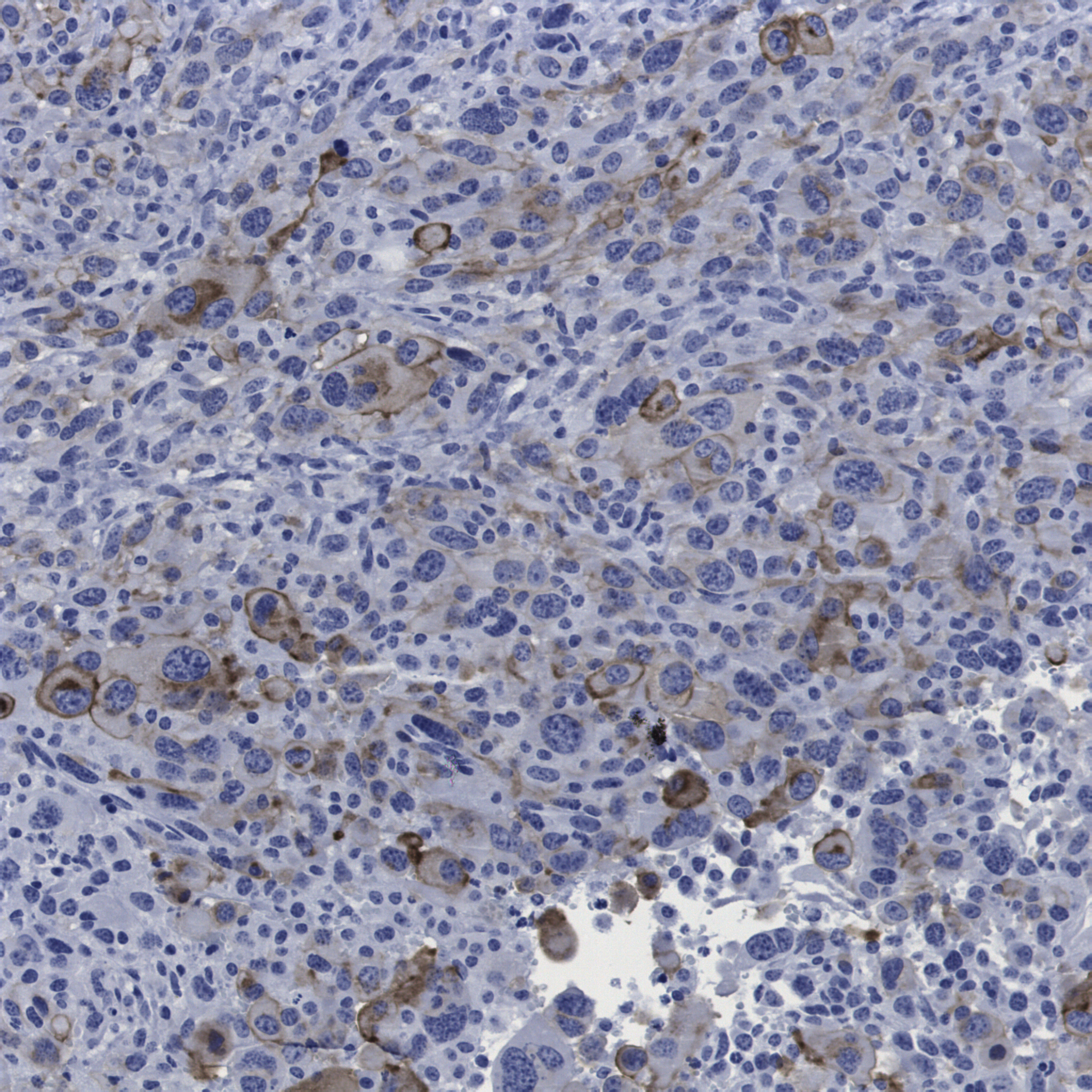

Supplement: Supplementary file 6 — Source Data for Figure 2 [file EMMM-12-e10941-s004.zip › Figure_2/KM2.tif]

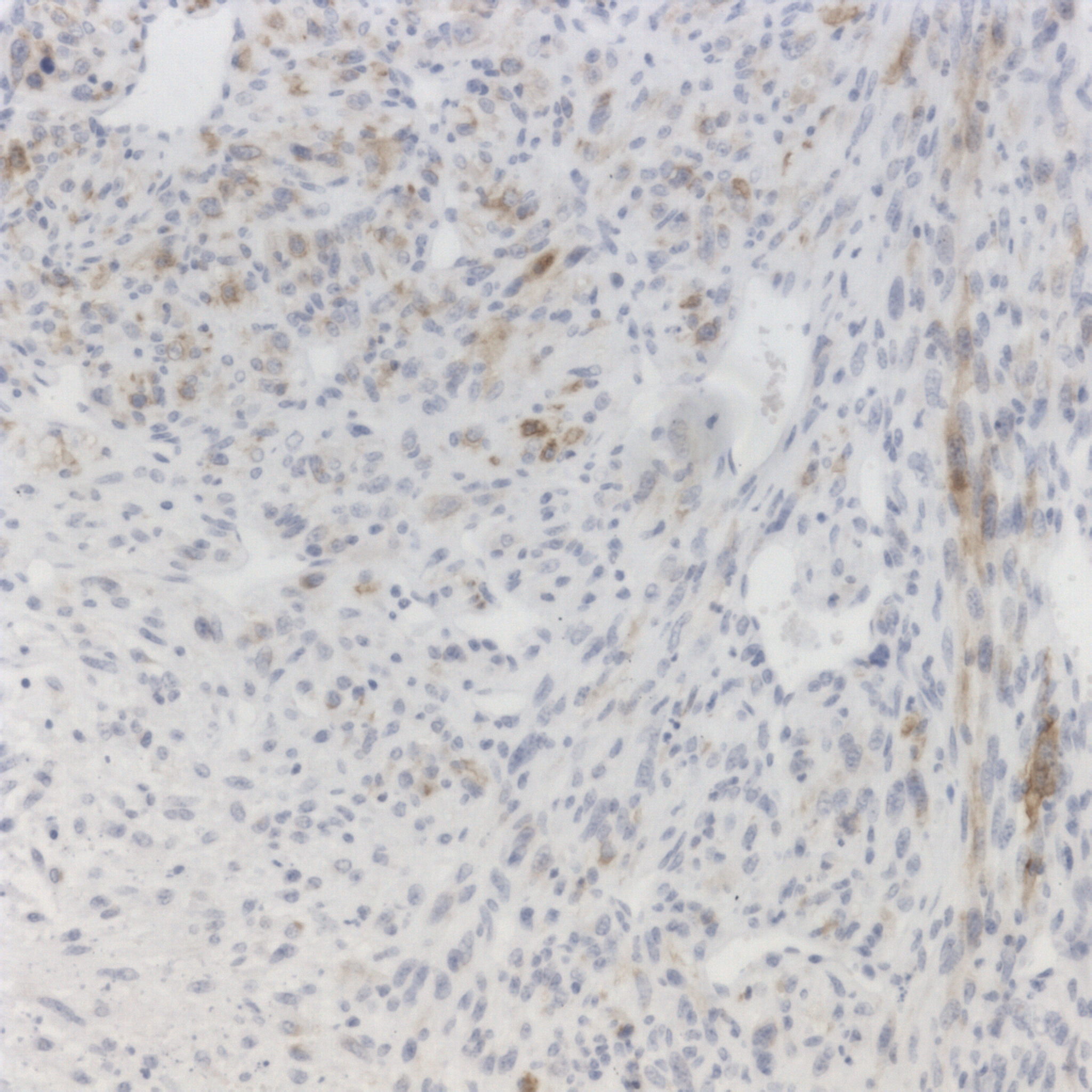

Supplement: Supplementary file 6 — Source Data for Figure 2 [file EMMM-12-e10941-s004.zip › Figure_2/KM20.tif]

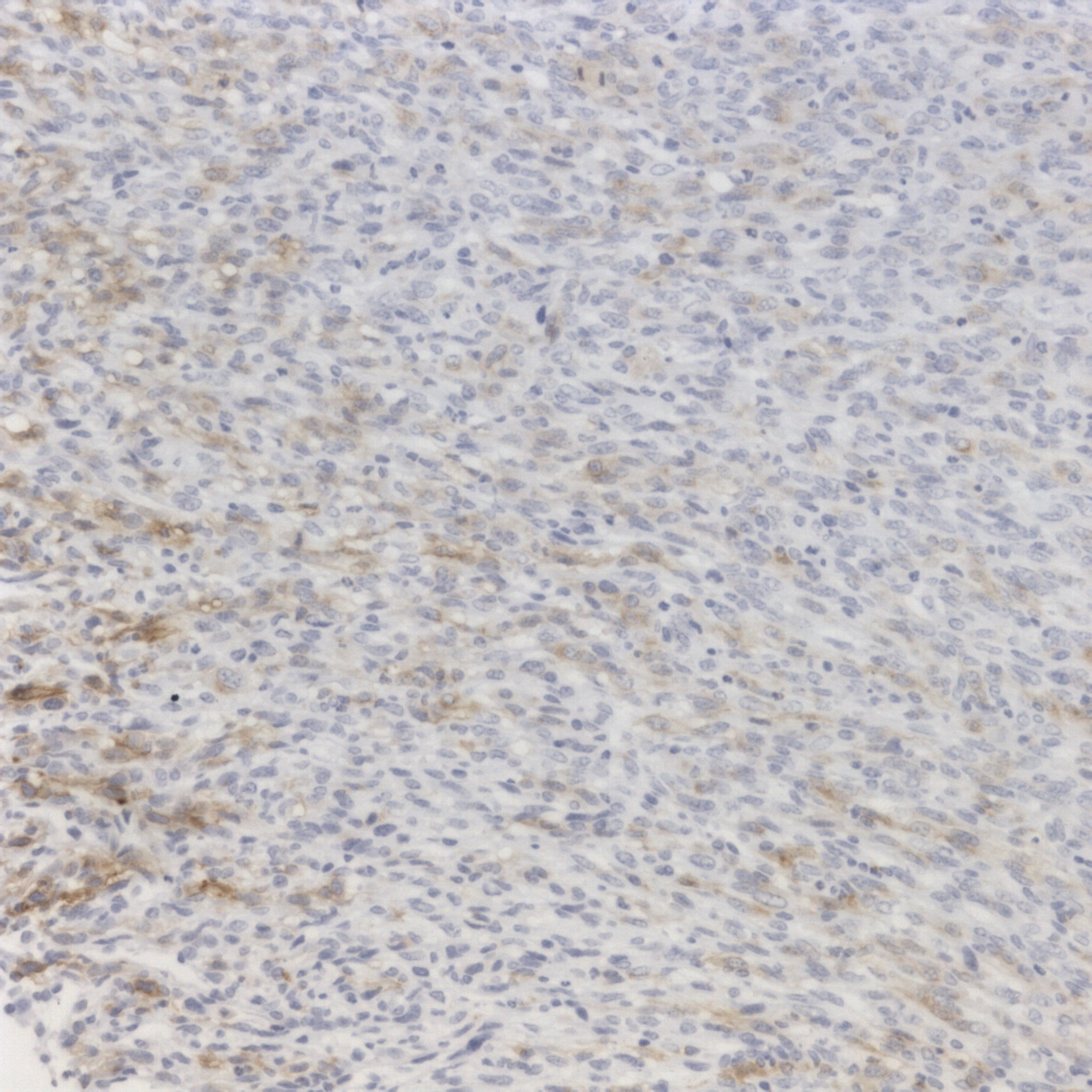

Supplement: Supplementary file 6 — Source Data for Figure 2 [file EMMM-12-e10941-s004.zip › Figure_2/KM21.tif]

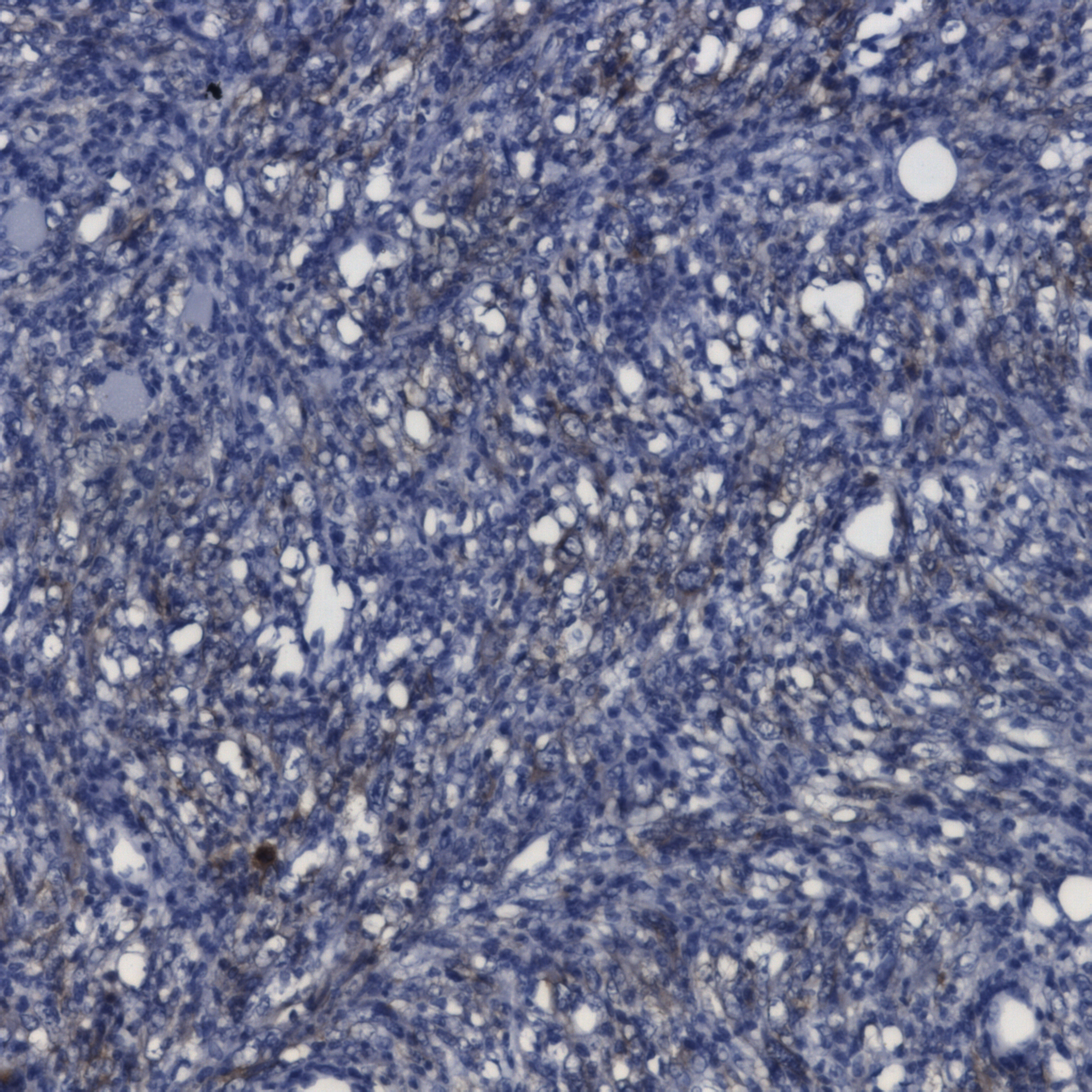

Supplement: Supplementary file 6 — Source Data for Figure 2 [file EMMM-12-e10941-s004.zip › Figure_2/KM22.tif]

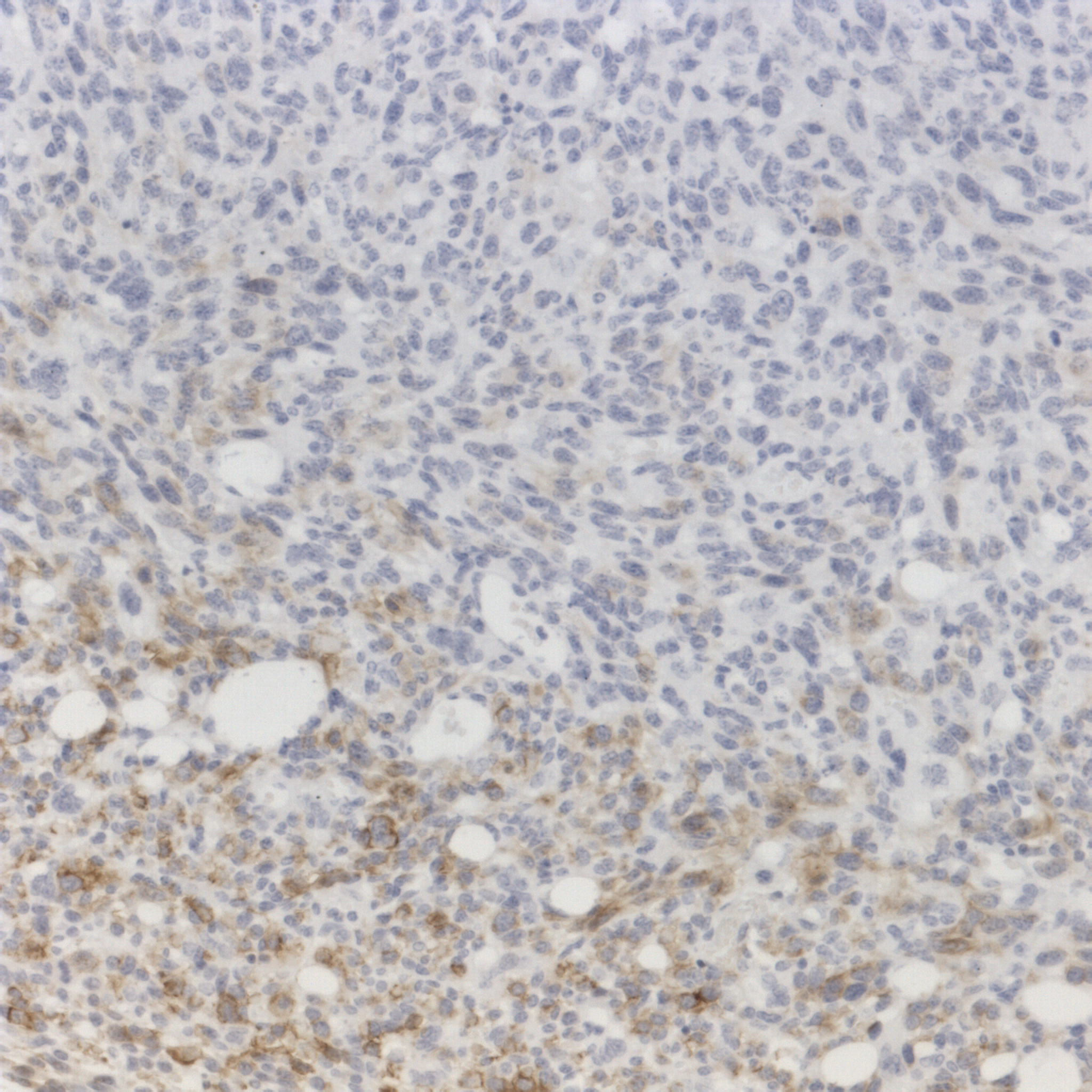

Supplement: Supplementary file 6 — Source Data for Figure 2 [file EMMM-12-e10941-s004.zip › Figure_2/KM3.tif]

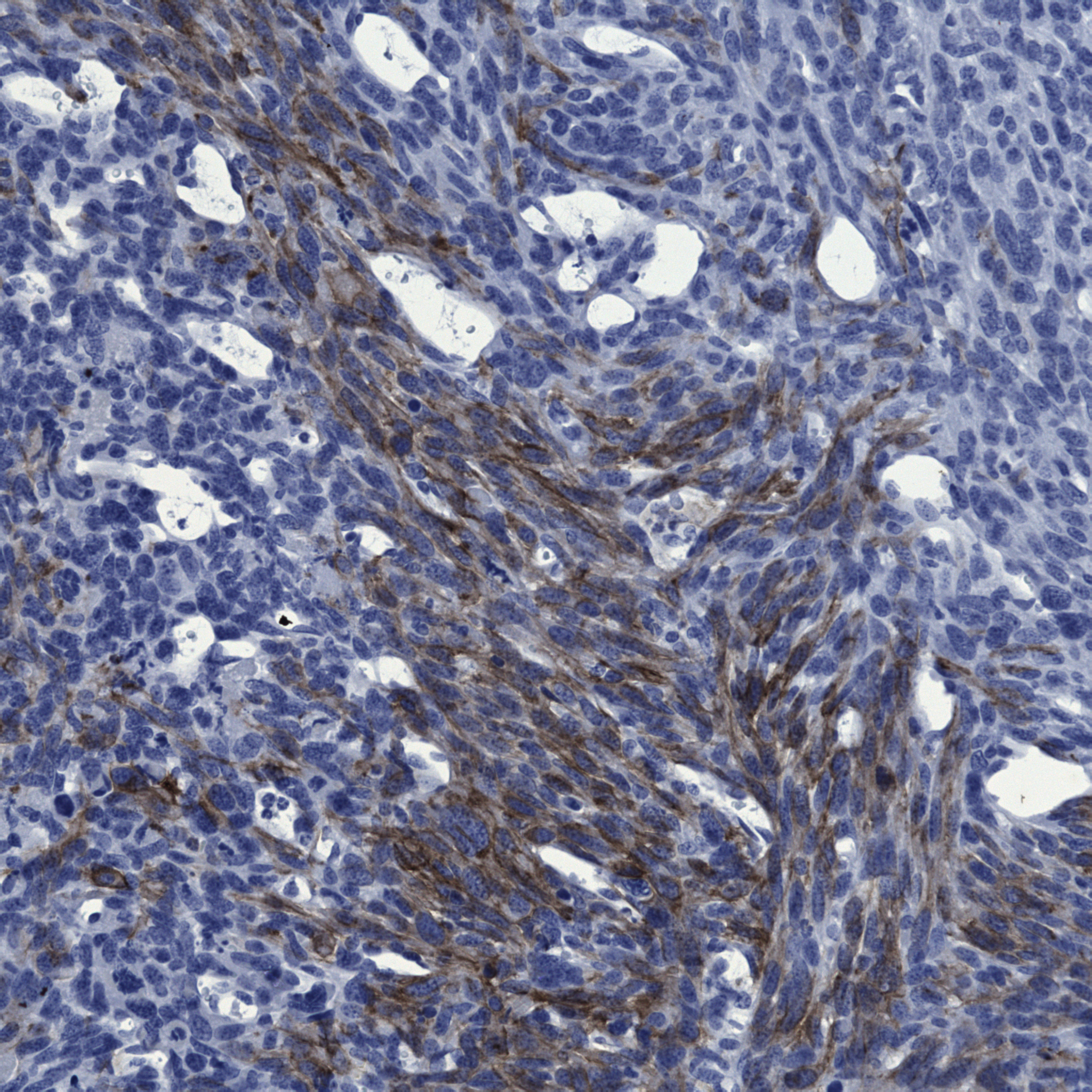

Supplement: Supplementary file 6 — Source Data for Figure 2 [file EMMM-12-e10941-s004.zip › Figure_2/KM5.tiff]

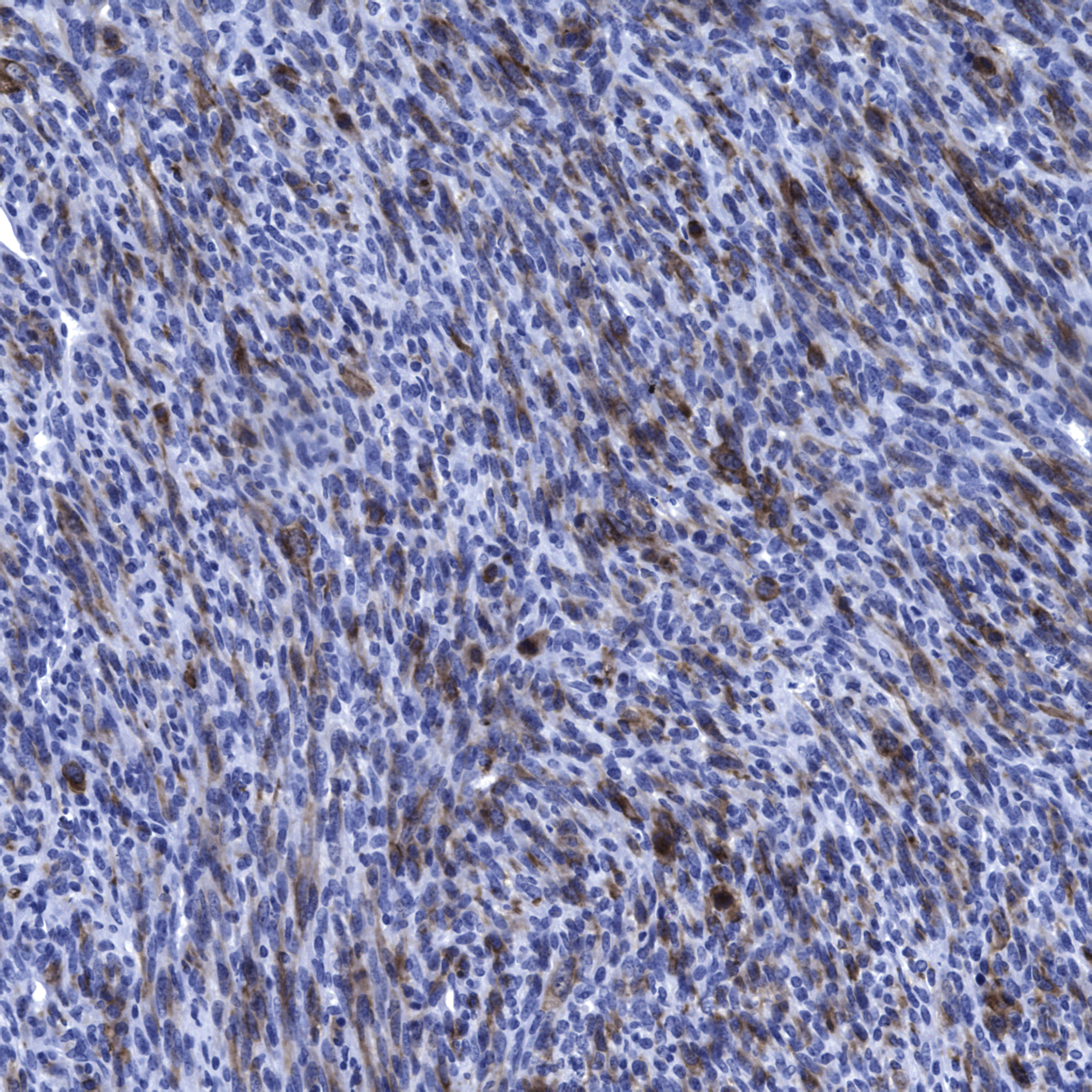

Supplement: Supplementary file 6 — Source Data for Figure 2 [file EMMM-12-e10941-s004.zip › Figure_2/KM7.tif]

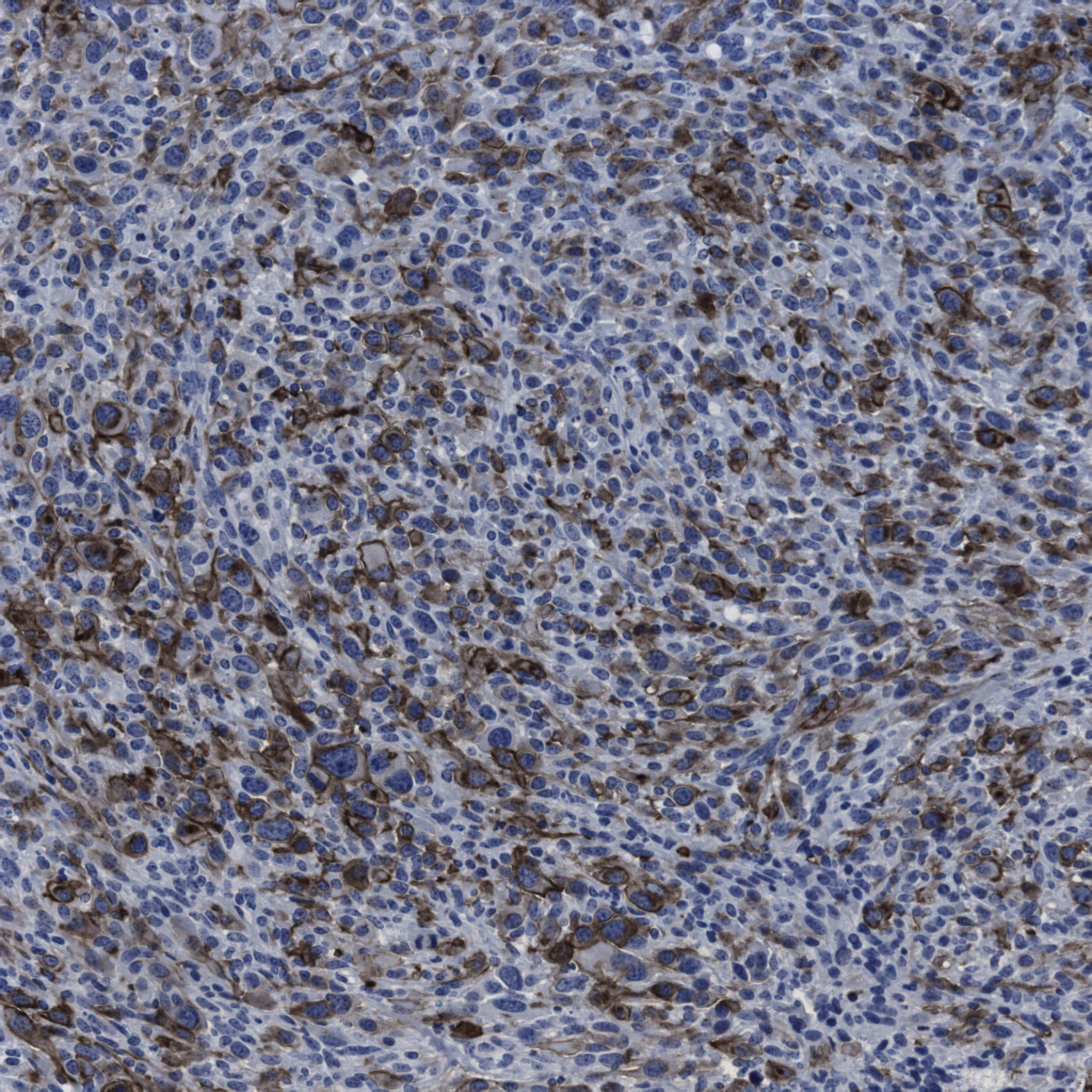

Supplement: Supplementary file 6 — Source Data for Figure 2 [file EMMM-12-e10941-s004.zip › Figure_2/KM8.tif]

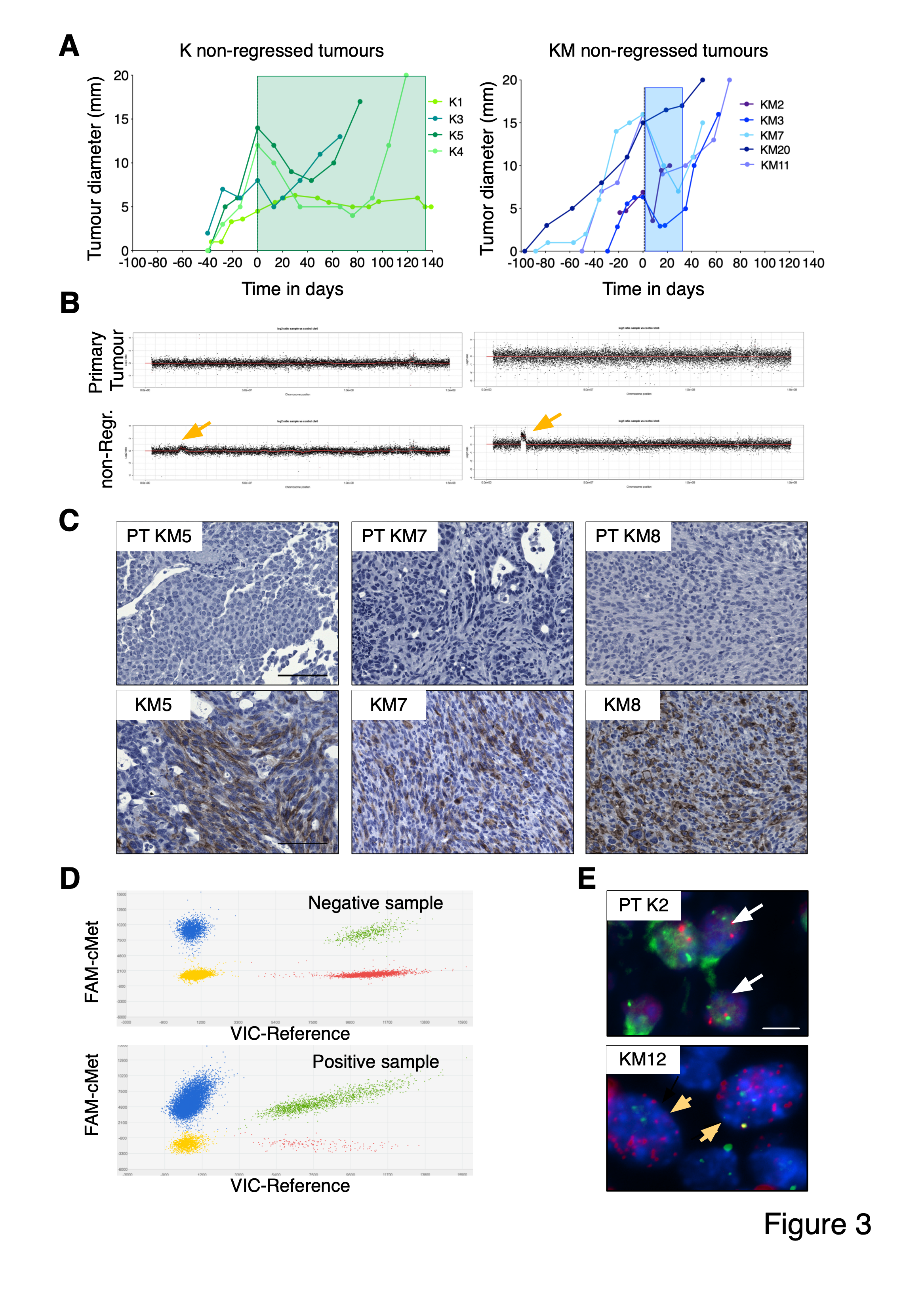

Supplement: Supplementary file 7 — Source Data for Figure 3 [file EMMM-12-e10941-s005.zip › Figure_3/Figure_3.tiff]

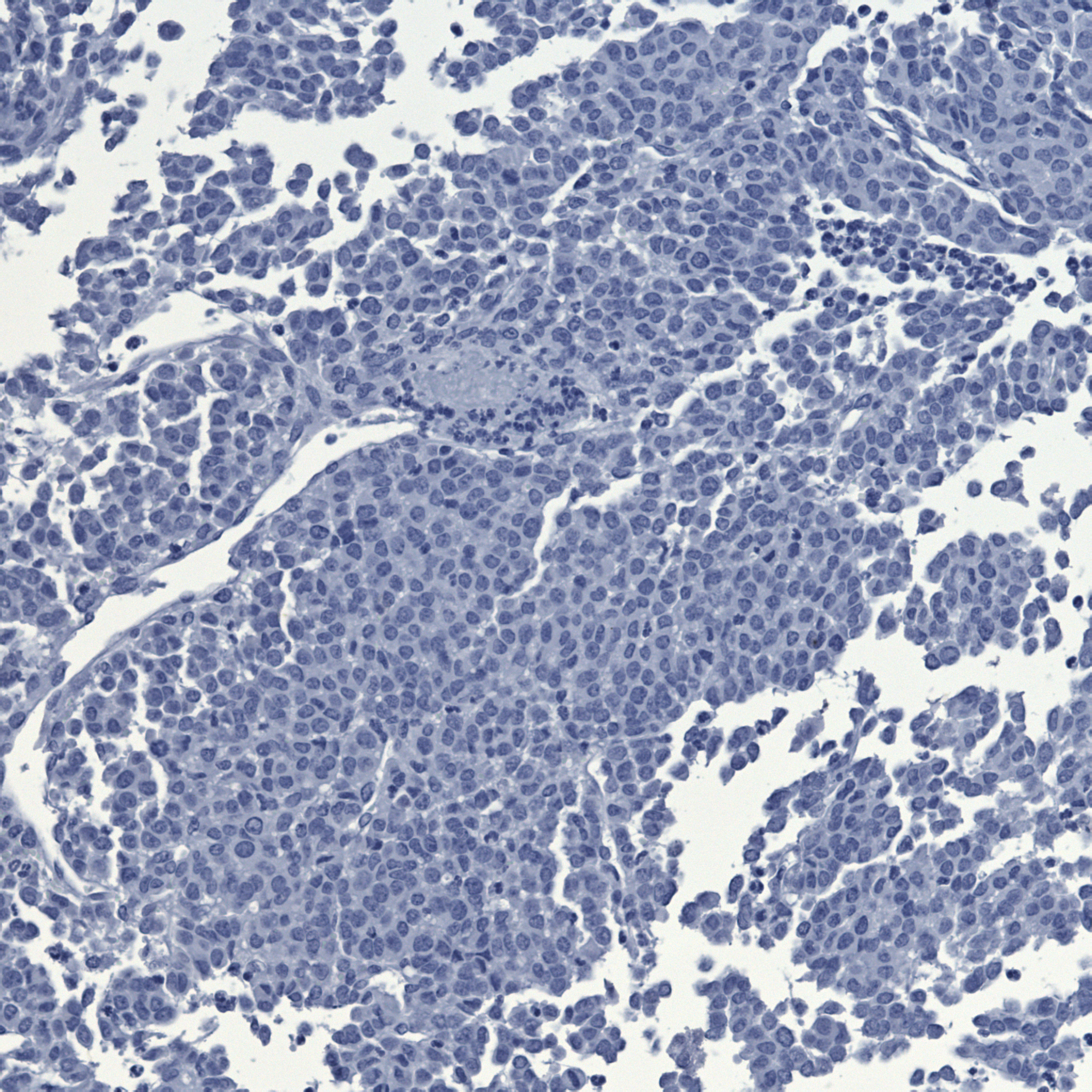

Supplement: Supplementary file 7 — Source Data for Figure 3 [file EMMM-12-e10941-s005.zip › Figure_3/Fig_3.C-PT_KM5.tiff]

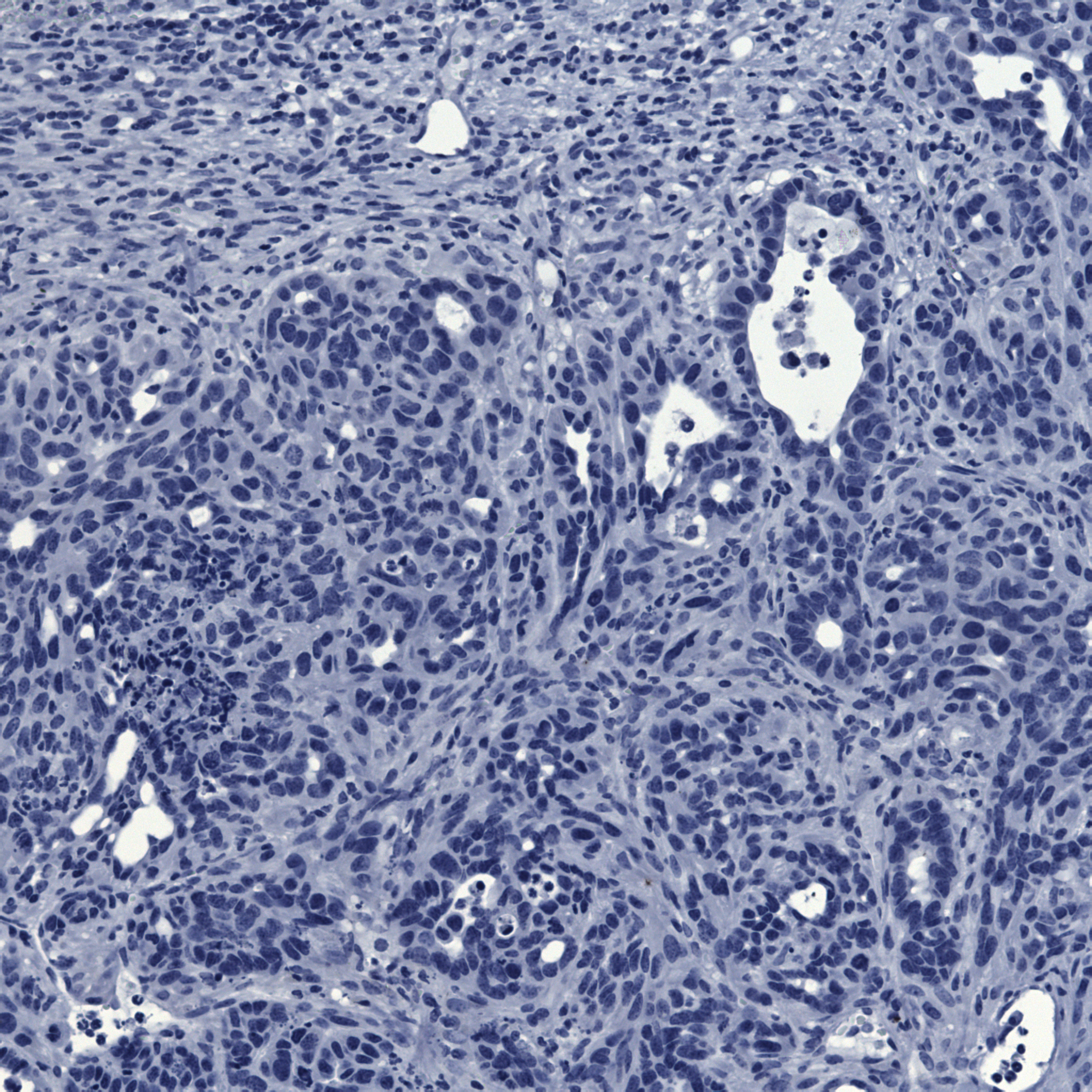

Supplement: Supplementary file 7 — Source Data for Figure 3 [file EMMM-12-e10941-s005.zip › Figure_3/Fig_3.C-PT_KM7.tiff]

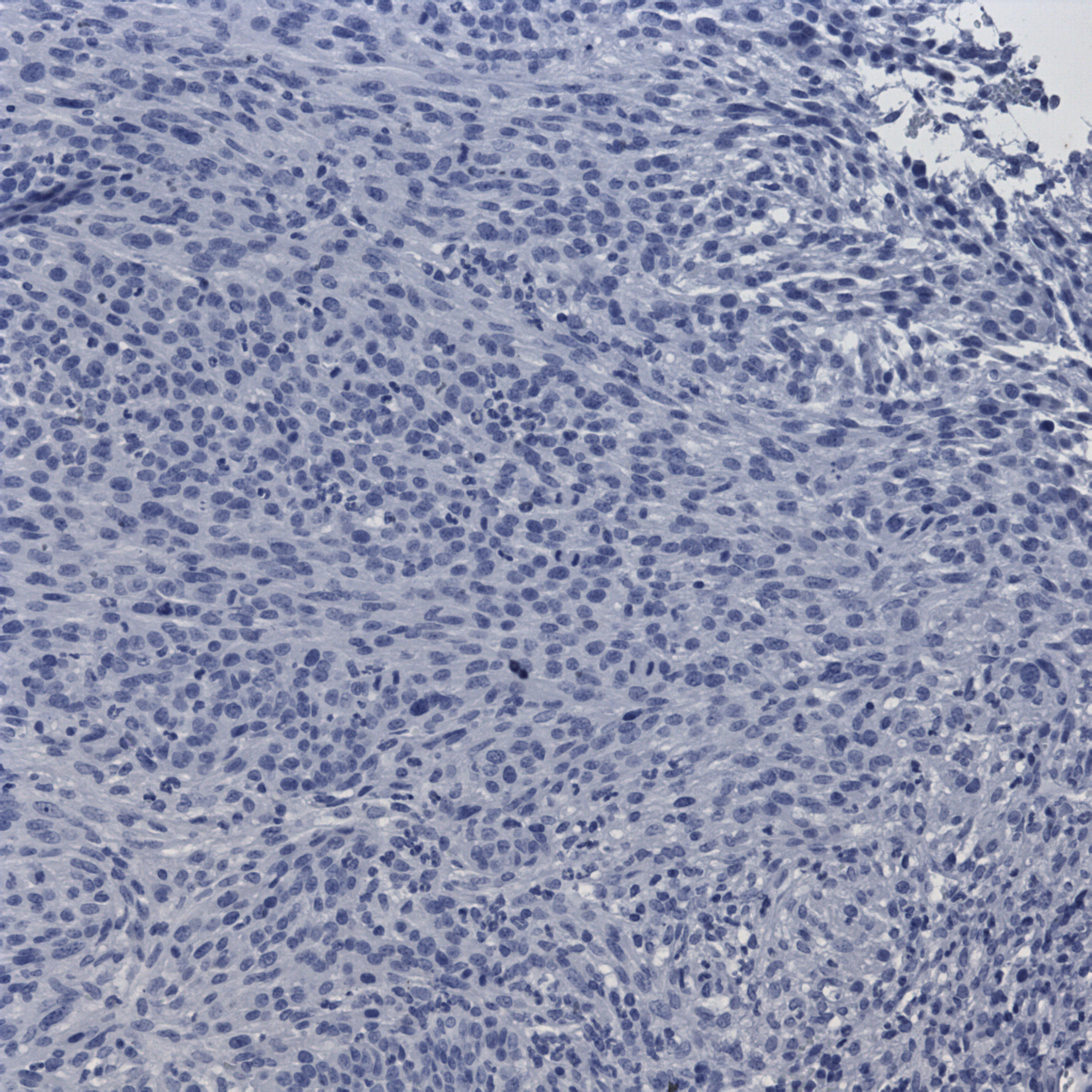

Supplement: Supplementary file 7 — Source Data for Figure 3 [file EMMM-12-e10941-s005.zip › Figure_3/Fig_3.C-PT_KM8.tif]

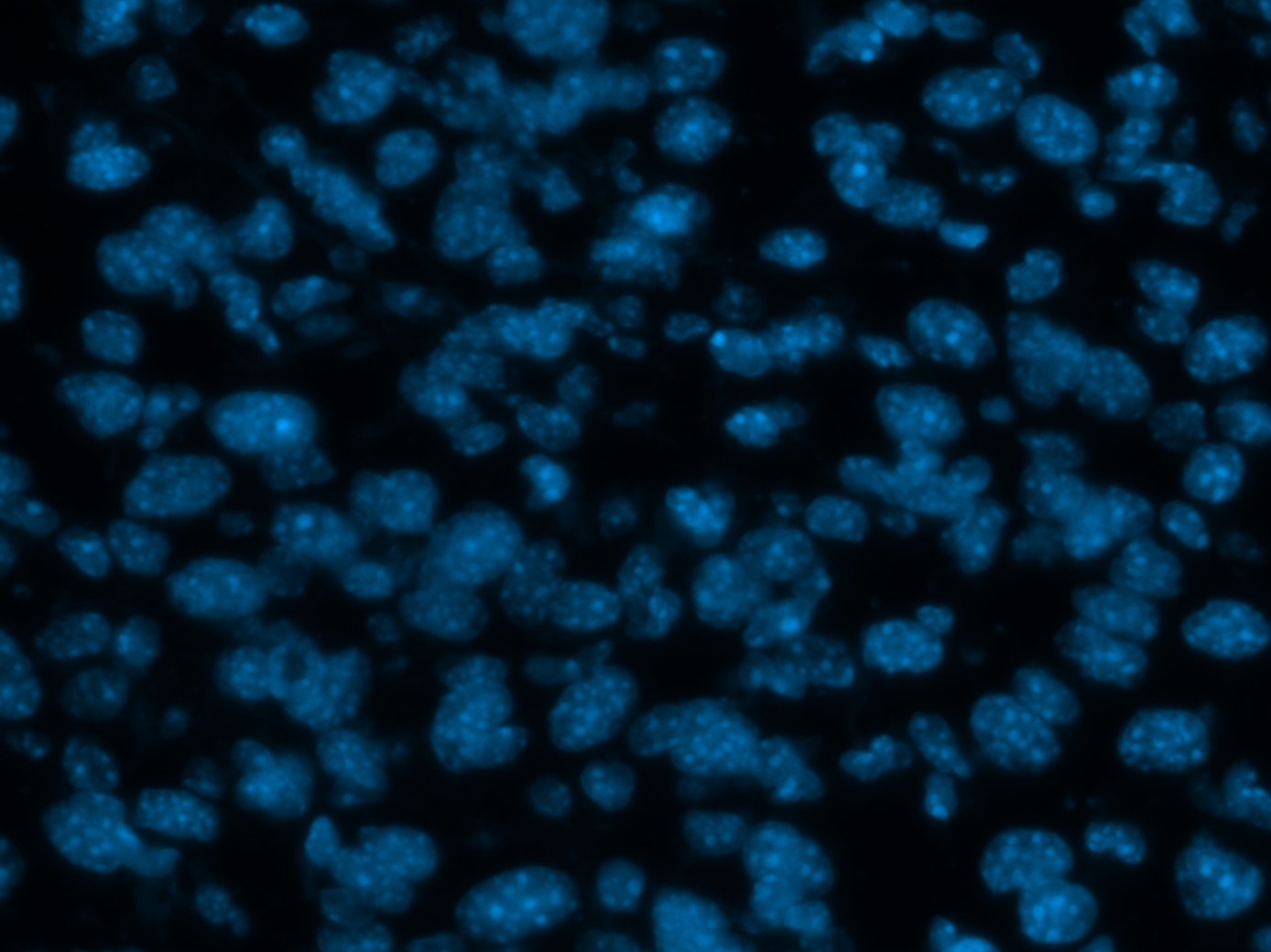

Supplement: Supplementary file 7 — Source Data for Figure 3 [file EMMM-12-e10941-s005.zip › Figure_3/Fig_3.E-KM12_Dapi.tif]

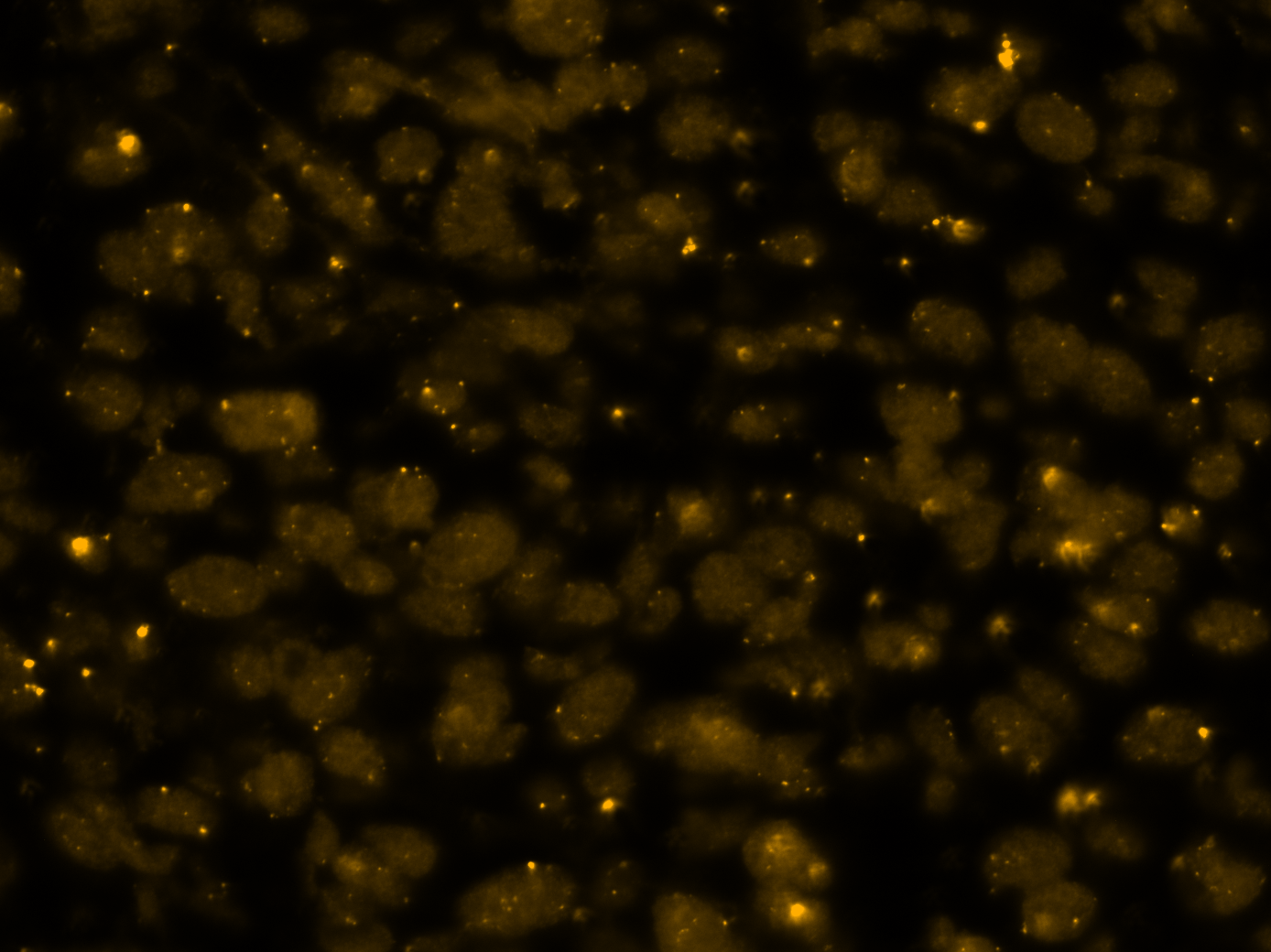

Supplement: Supplementary file 7 — Source Data for Figure 3 [file EMMM-12-e10941-s005.zip › Figure_3/Fig_3.E-KM12_Reference_probe.tif]

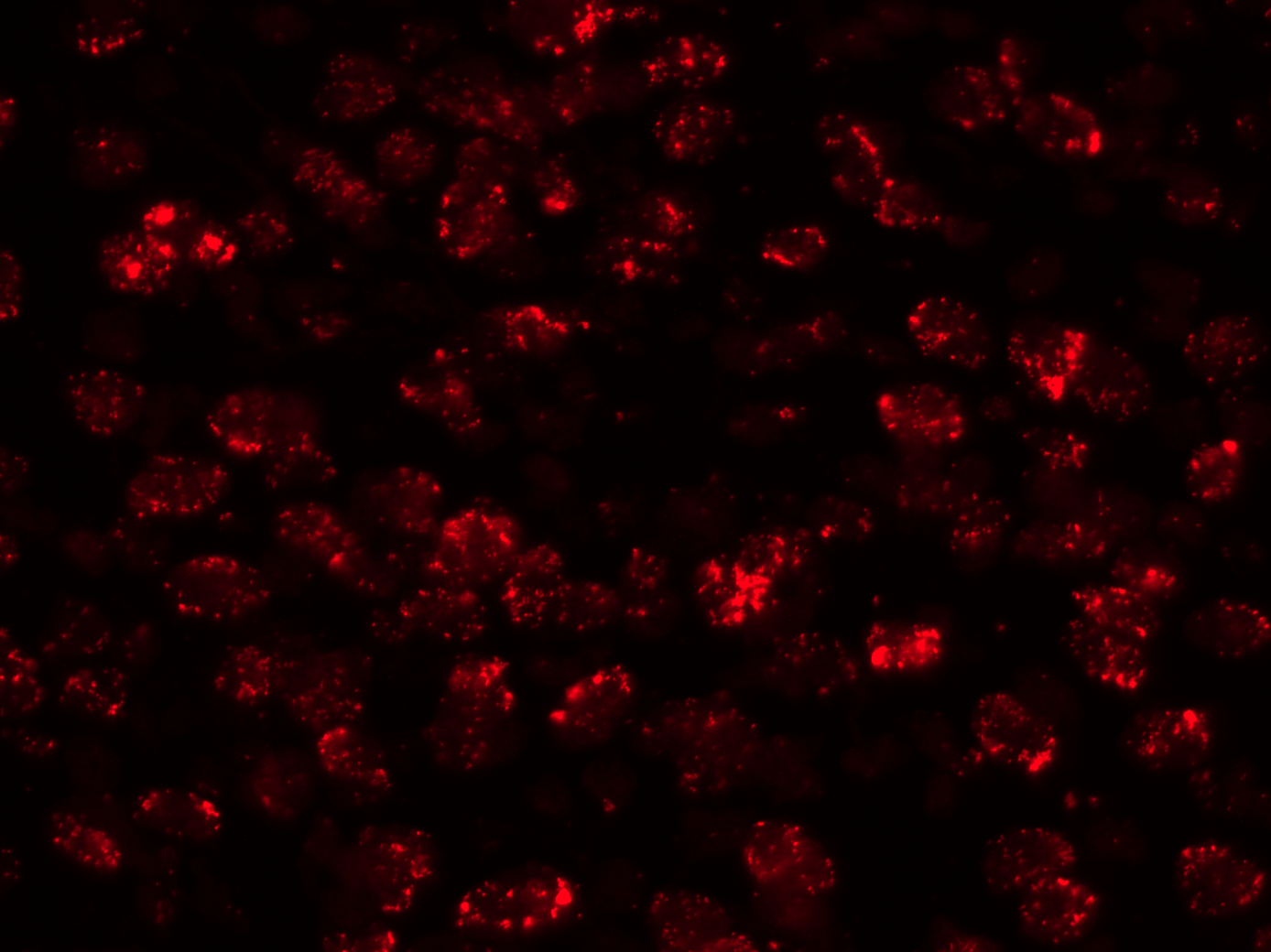

Supplement: Supplementary file 7 — Source Data for Figure 3 [file EMMM-12-e10941-s005.zip › Figure_3/Fig_3.E-KM_12_cMet_probe.tif]

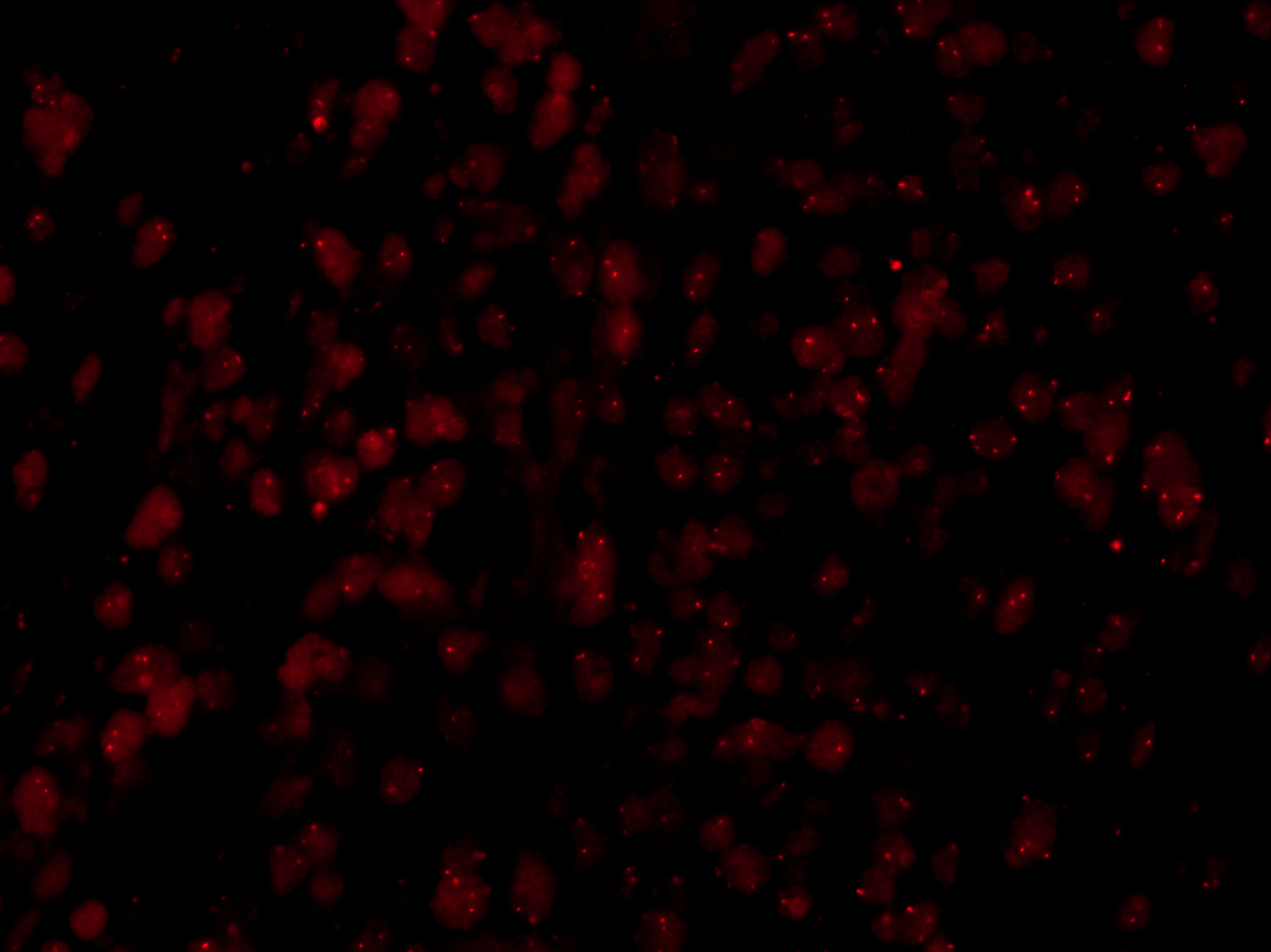

Supplement: Supplementary file 7 — Source Data for Figure 3 [file EMMM-12-e10941-s005.zip › Figure_3/Fig_3.E-_PT_K2_cMet_probe.tif]

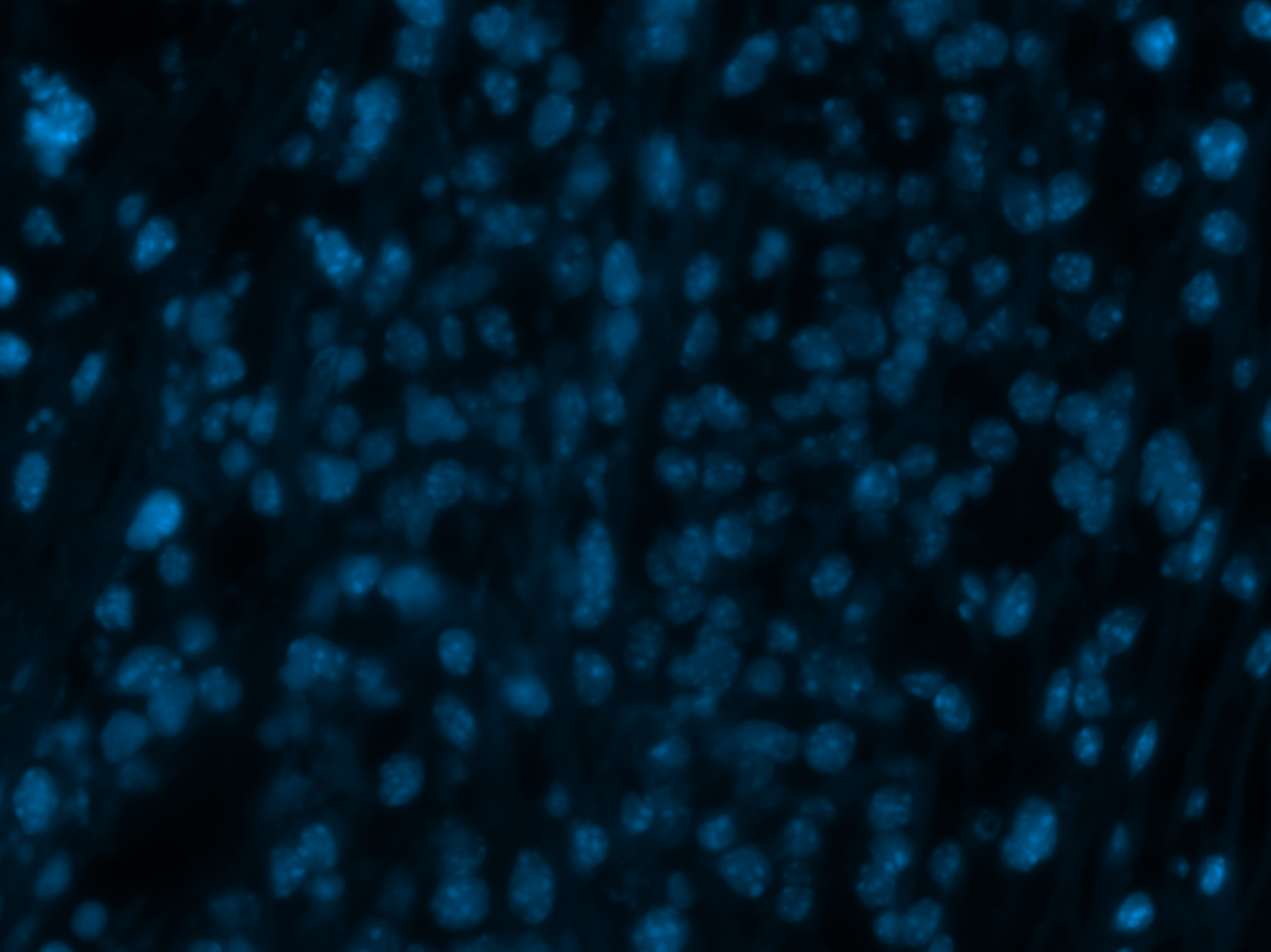

Supplement: Supplementary file 7 — Source Data for Figure 3 [file EMMM-12-e10941-s005.zip › Figure_3/Fig_3.E-_PT_K2_Dapi.tif]

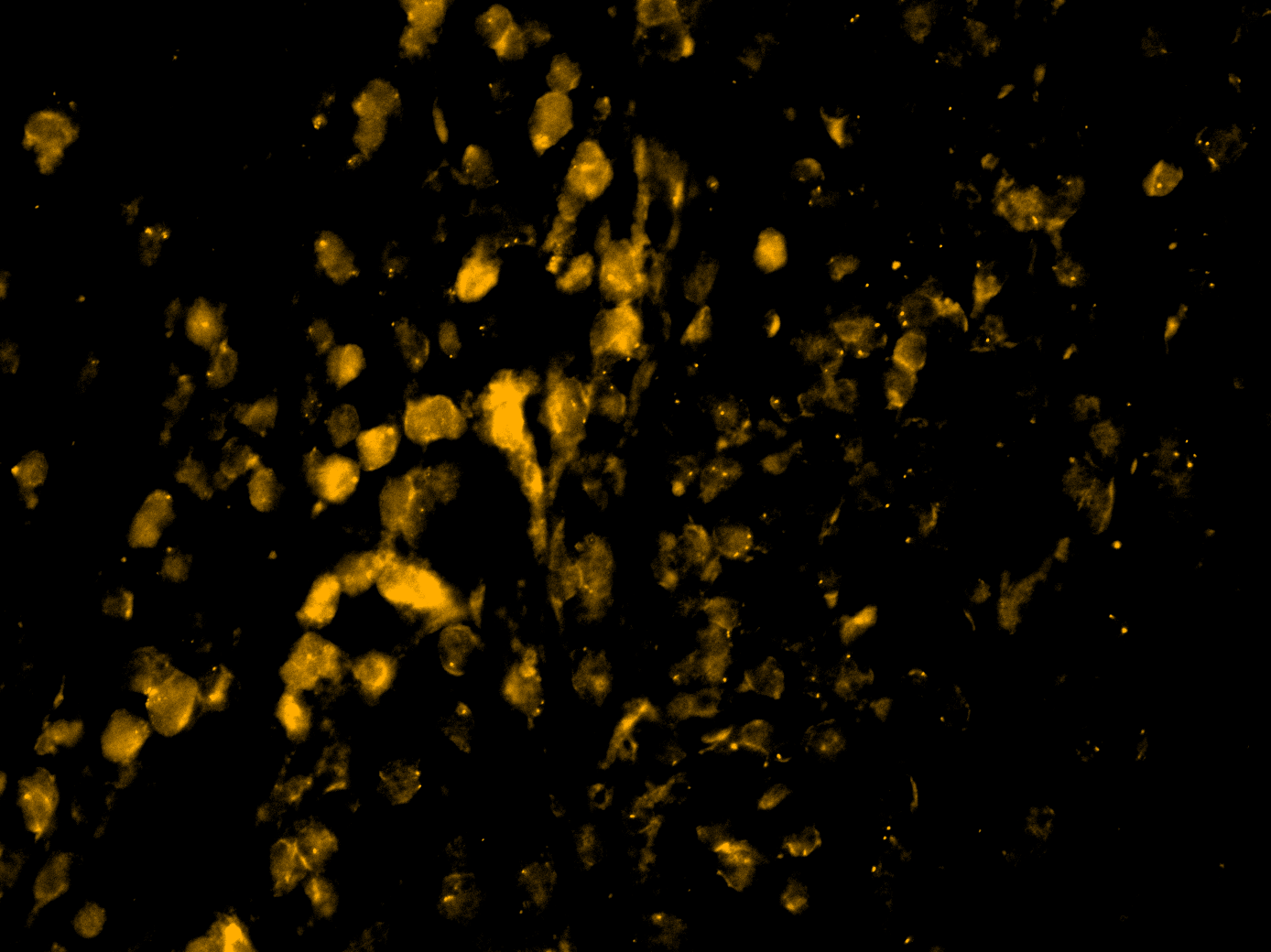

Supplement: Supplementary file 7 — Source Data for Figure 3 [file EMMM-12-e10941-s005.zip › Figure_3/Fig_3.E-_PT_K2_Reference_probe.tif]

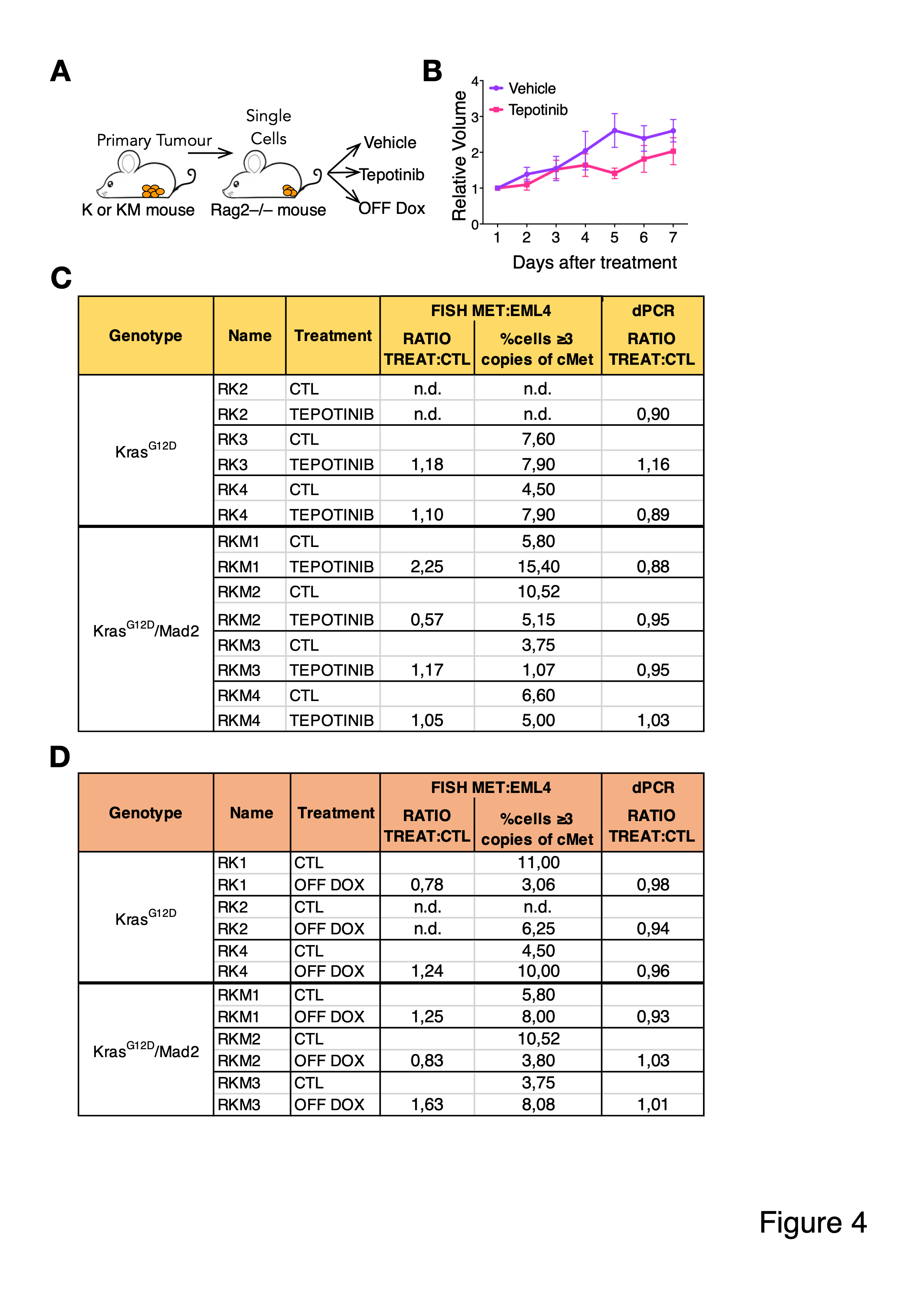

Supplement: Supplementary file 8 — Source Data for Figure 4 [file EMMM-12-e10941-s006.zip › Figure_4/Figure_4.tiff]

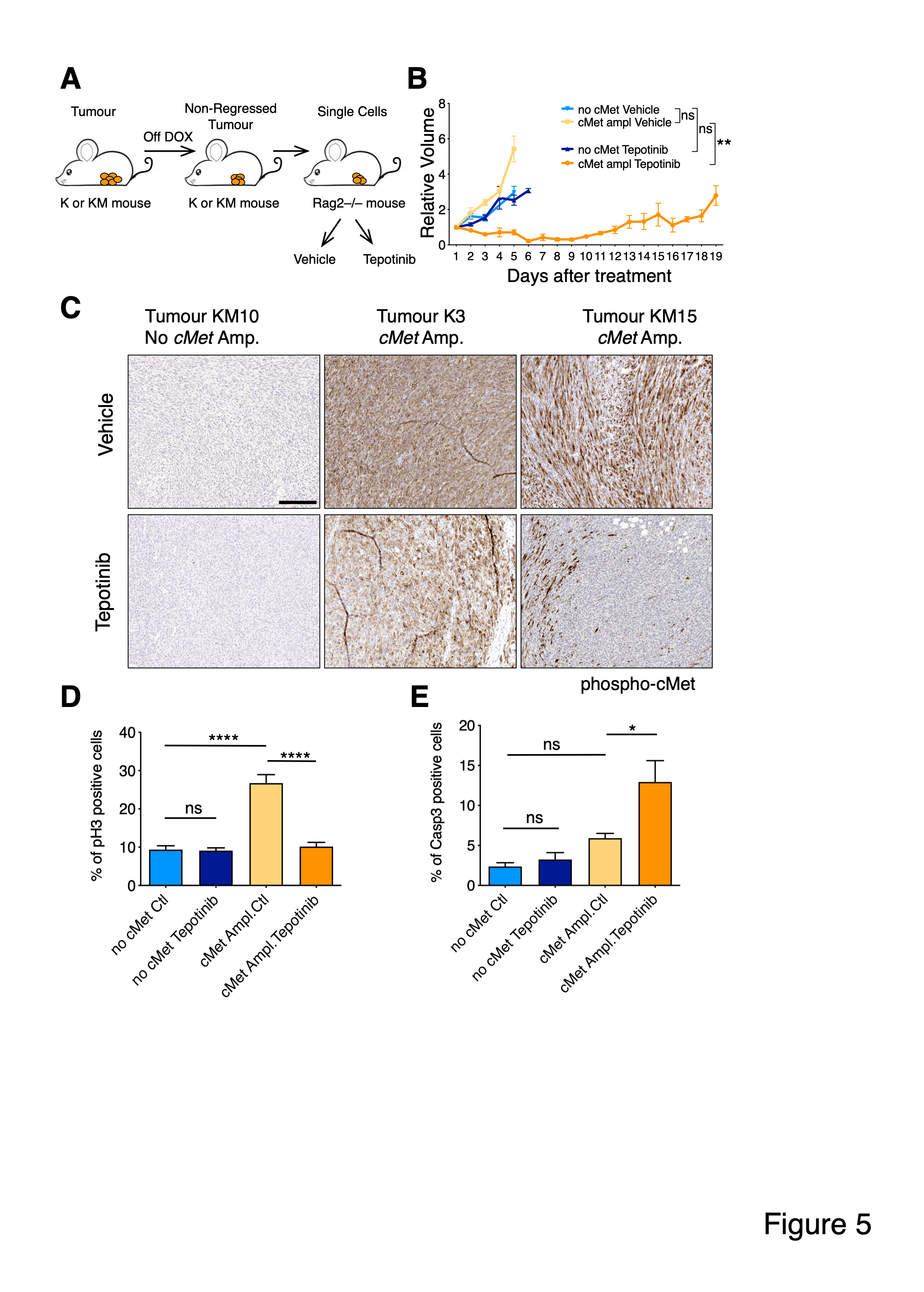

Supplement: Supplementary file 9 — Source Data for Figure 5 [file EMMM-12-e10941-s007.zip › Figure_5/Figure_5.tiff]

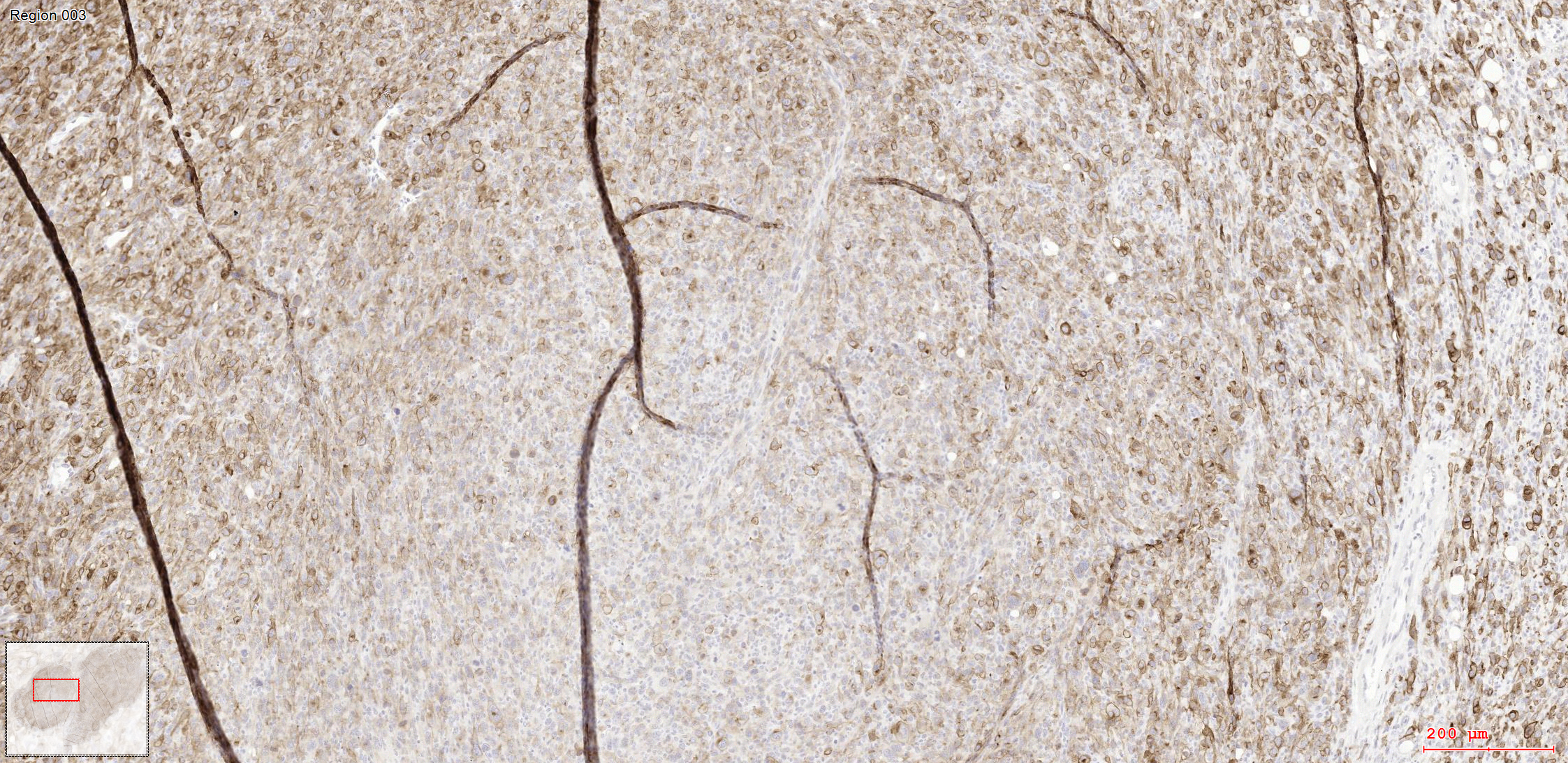

Supplement: Supplementary file 9 — Source Data for Figure 5 [file EMMM-12-e10941-s007.zip › Figure_5/phospho-cMet_K3._Tepotinib.TIFF]

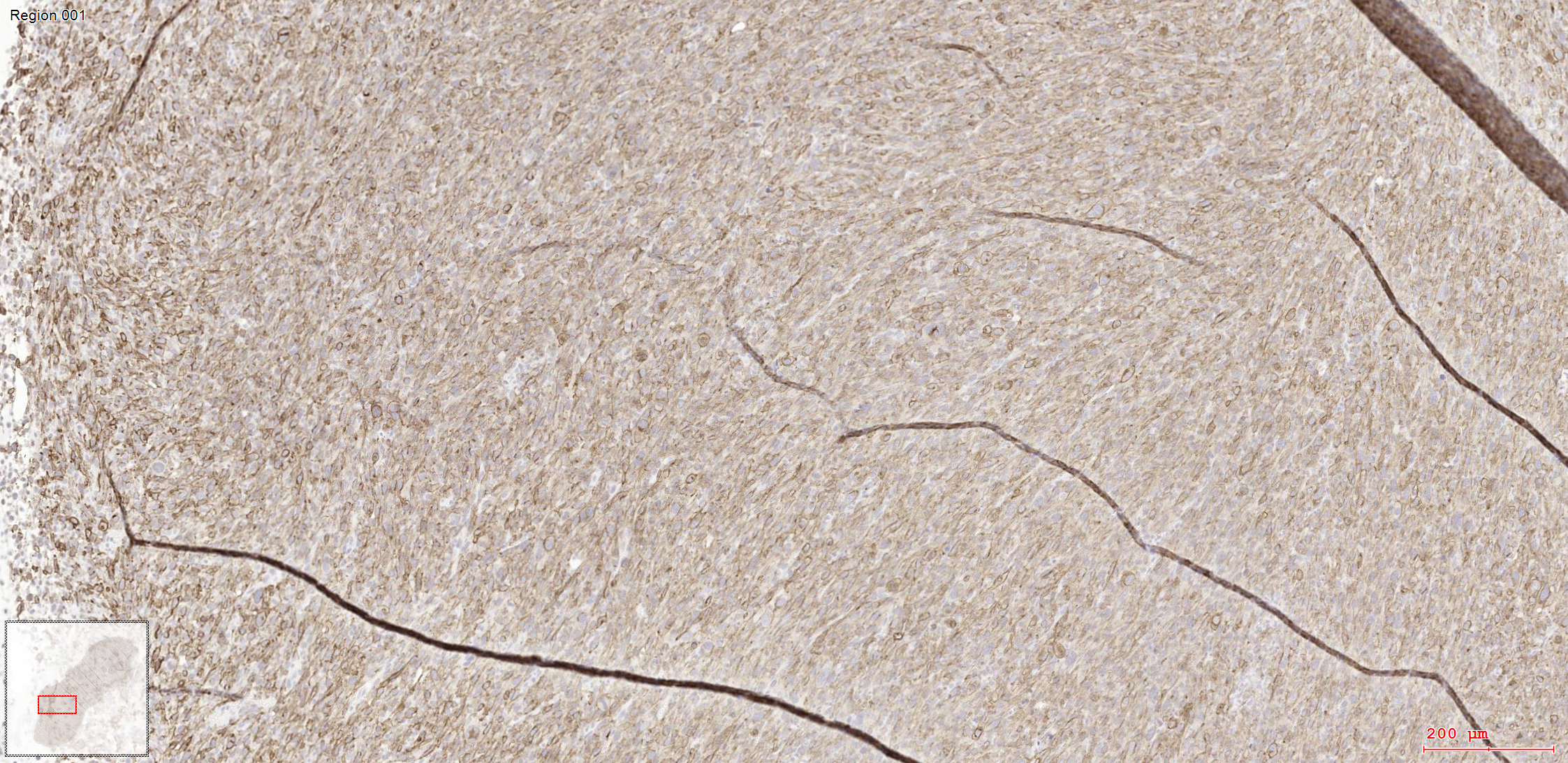

Supplement: Supplementary file 9 — Source Data for Figure 5 [file EMMM-12-e10941-s007.zip › Figure_5/phospho-cMet_K3._Vehicle.TIFF]

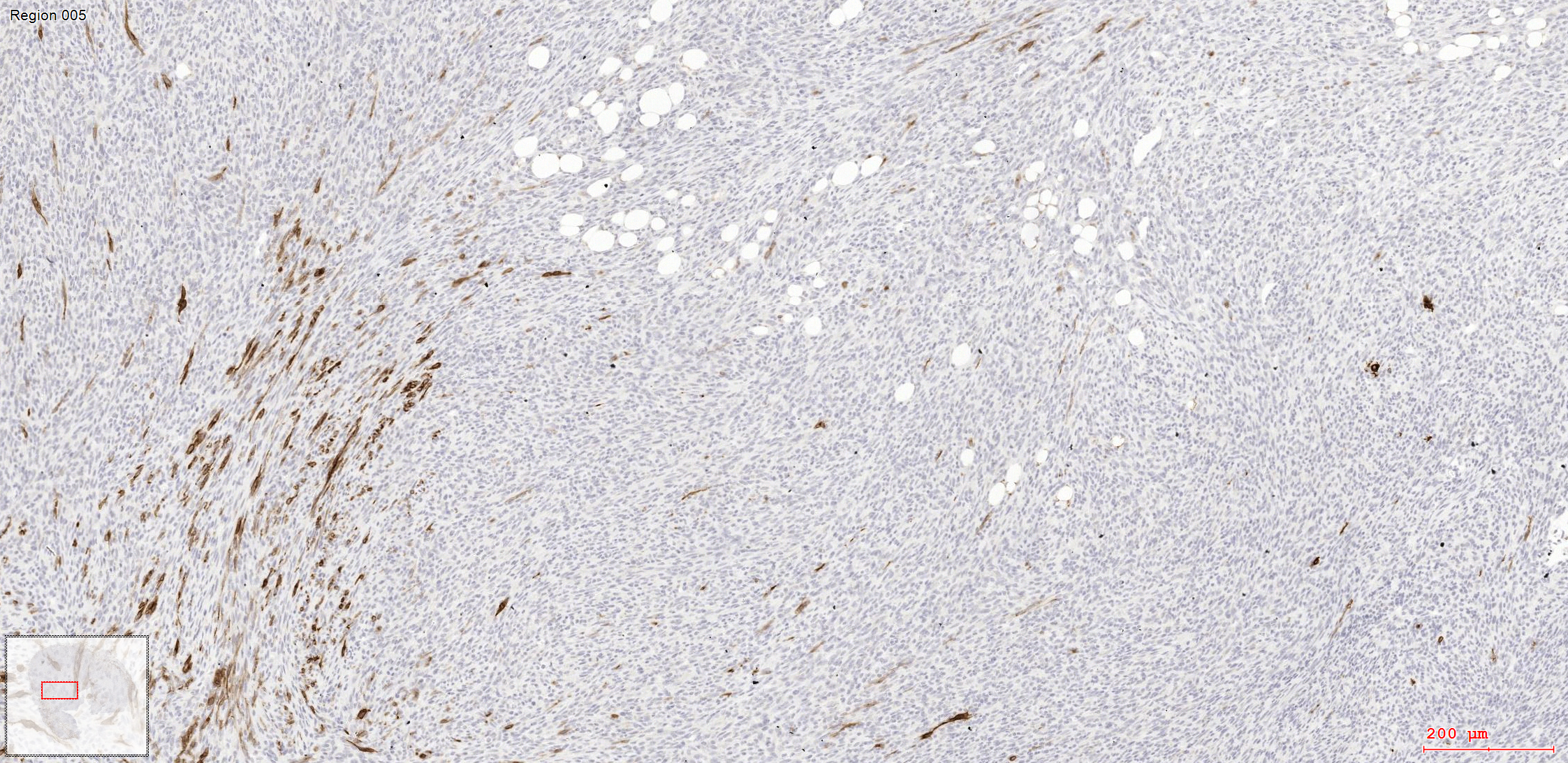

Supplement: Supplementary file 9 — Source Data for Figure 5 [file EMMM-12-e10941-s007.zip › Figure_5/phospho-cMet_KM15._Tepotinib.TIFF]
