# Supplementary figures and images for: Acquisition of chromosome instability is a mechanism to evade oncogene addiction (part 2 of 2)
Source: EMBO Mol Med. 2020 Feb 6;12(3):e10941. doi: 10.15252/emmm.201910941 (PMC7059010; doi:10.15252/emmm.201910941)

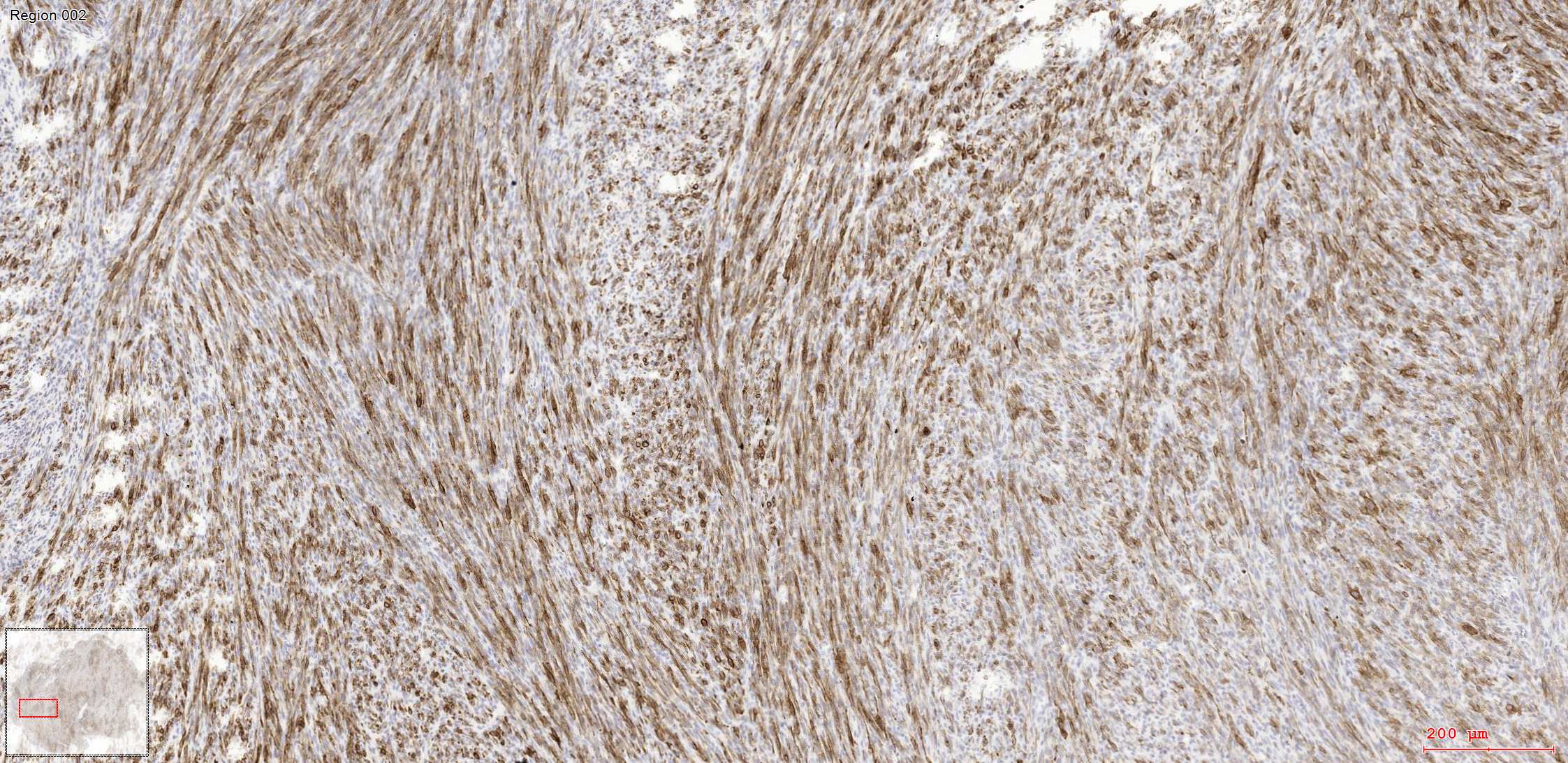

Supplement: Supplementary file 9 — Source Data for Figure 5 [file EMMM-12-e10941-s007.zip › Figure_5/phospho-cMet_KM15._Vehicle.TIFF]

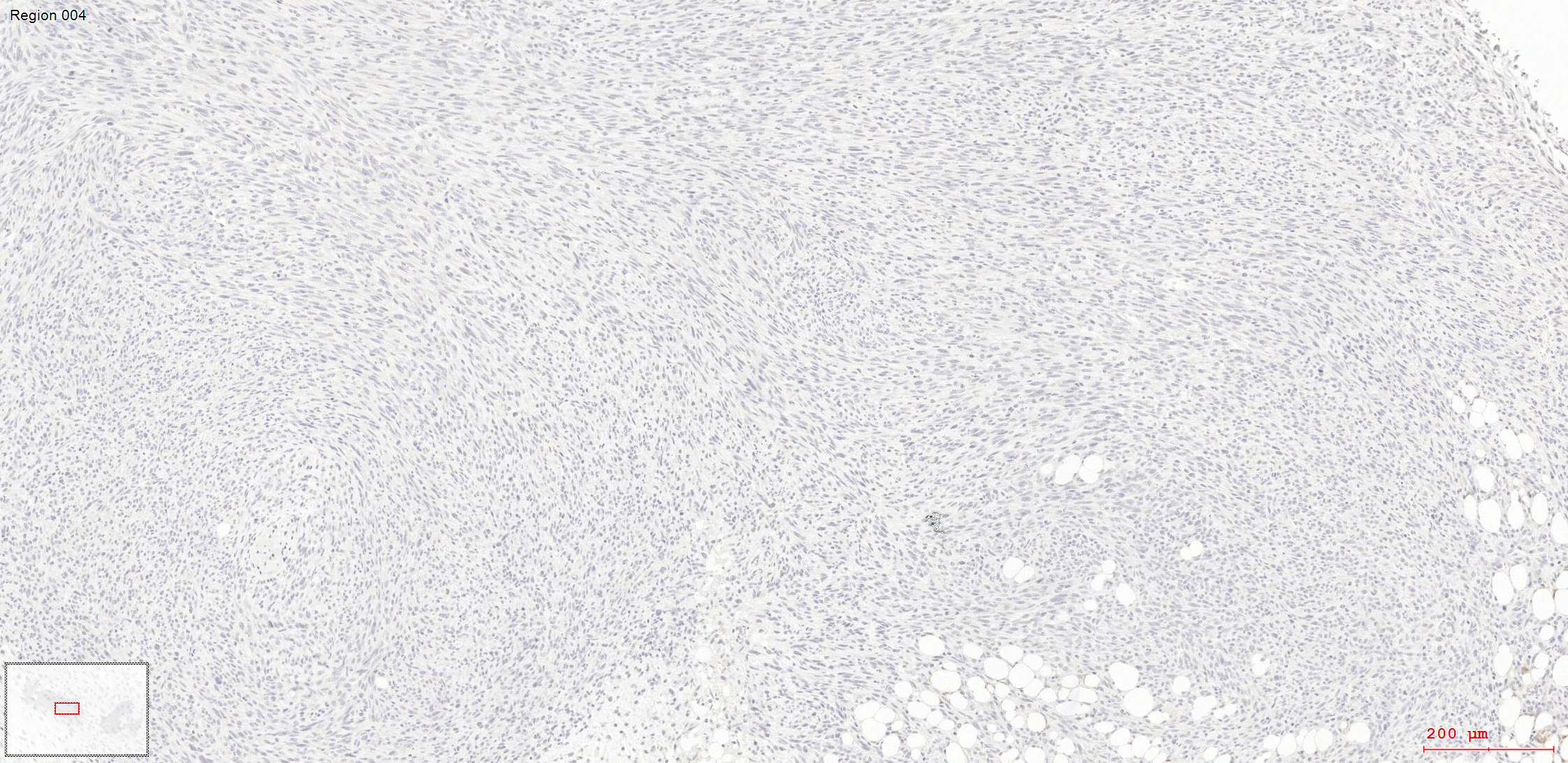

Supplement: Supplementary file 9 — Source Data for Figure 5 [file EMMM-12-e10941-s007.zip › Figure_5/phospho_c-Met._KM10._Vehicle.TIFF]

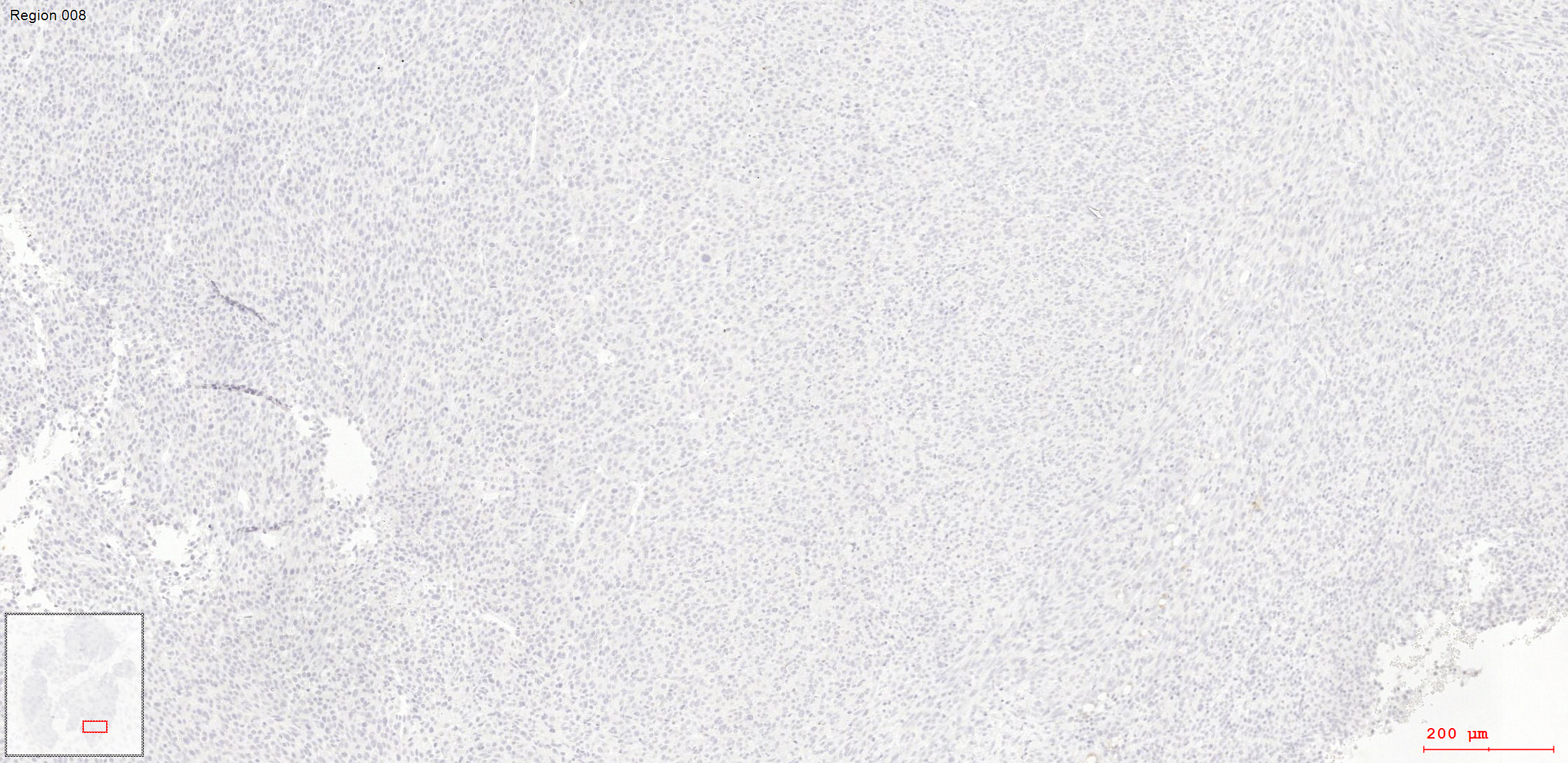

Supplement: Supplementary file 9 — Source Data for Figure 5 [file EMMM-12-e10941-s007.zip › Figure_5/phospho_cMet._KM10._Tepotinib.TIFF]
